# Supplementary material for: Common protocol for validation of the QCOVID algorithm across the four UK nations
Source: BMJ Open. 2022 Jun 14;12(6):e050994. doi: 10.1136/bmjopen-2021-050994 (PMC9198386; doi:10.1136/bmjopen-2021-050994)
Supplement: Supplementary data [file bmjopen-2021-050994supp001.pdf]

BMJ Publishing Group Limited (BMJ) disclaims all liability and responsibility

Supplemental material placed on this supplemental material which has been supplied by the autho[illegible] BMJ Open

Sheet3b - Chemotherapy lookup

final classification	map
Group A	1 & 2
Group B	3 & 4
Group C	5

Bench Mark Group	Group
TRASTUZUMAB	1
ZOLEDRONIC ACID	1
DENOSUMAB	1
PEMBROLIZUMAB	1
PERTUZUMAB + TRASTUZUMAB	1
ENZALUTAMIDE	1
ABIRATERONE	1
NIVOLUMAB	1
CETUXIMAB	1
ATEZOLIZUMAB	1
BCG	1
BEVACIZUMAB	1
AFATINIB	1
FULVESTRANT	1
IPILIMUMAB + NIVOLUMAB	1
GOSERELIN	1
METHOTREXATE INTRATHECAL	1
PANITUMUMAB	1
LANREOTIDE	1
DURVALUMAB	1
PERTUZUMAB	1
PAMIDRONATE	1
MITOMYCIN INTRAVESICULAR	1
OCTREOTIDE	1
IMMUNOGLOBULIN	1
ANAGRELIDE	1
LETROZOLE	1
AVELUMAB	1
TRIPLE INTRATHECAL	1
CETUXIMAB + RT	1
BICALUTAMIDE	1
TRIPTORELIN	1
IPILIMUMAB	1
PRISM TRIAL	1
CEMIPLIMAB	1
NERATINIB	1
ANASTROZOLE	1
VISMODEGIB	1
IBANDRONIC ACID	1
TAMOXIFEN	1
CYTARABINE INTRATHECAL	1
EXEMESTANE	1
CYPROTERONE	1
TALIMOGENE LAHERPAREPVEC	1
LEUPRORELIN	1
DEGARELIX	1
ELOQUENT2 TRIAL	1
SILTUXIMAB	1
ELOQUENT TRIAL	1
BICALUTAMIDE + LEUPRORELIN	1
CONFIRM TRIAL	1
FAKTION TRIAL	1
APALUTAMIDE	1
EPIRUBICIN INTRAVESICULAR	1
STILBOESTROL	1
DARBEPOETIN	1
FLUOROURACIL + PANITUMUMAB	1
AVAPRITINIB	1
FURVA TRIAL	1
PERM TRIAL	1
ENZARAD TRIAL	1
BICALUTAMIDE + GOSERELIN	1
NB 13 CIS-RA (ISOTRETINOIN)	1
VORINOSTAT	1
ABIRATERONE + ENZALUTAMIDE	1
PARAGON TRIAL	1
MEDROXYPROGESTERONE	1
KESTREL TRIAL	1
MEGESTROL	1
XILONIX	1
RAMUCIRUMAB	1
CAM-PLEX TRIAL	1
BICALUTAMIDE + TAMOXIFEN	1
LATITUDE TRIAL	1
IPILIMUMAB + PEMBROLIZUMAB	1
QUADRUPLE INTRATHECAL	1
GOSERELIN + LEUPRORELIN	1
MERCAPTOPURINE + VINCRISTINE	1
PROGESTERONE	1
CLODRONIC ACID	1
CYPROTERONE + GOSERELIN	1
GOSERELIN + LETROZOLE	1
CISPLATIN + GEMCITABINE	2
HYDROXYCARBAMIDE	2
CAPECITABINE	2
CISPLATIN + RT	2
PALBOCICLIB	2
FLUOROURACIL	2
CISPLATIN	2
CAPECITABINE + IRINOTECAN	2
CISPLATIN + PEMETREXED	2
DABRAFENIB + TRAMETINIB	2
CAPECITABINE + GEMCITABINE	2
LETROZOLE + PALBOCICLIB	2
PEMETREXED	2
VINORELBINE	2
CISPLATIN + FLUOROURACIL	2
EVEROLIMUS	2
PAZOPANIB	2
SUNITINIB	2
ABEMACICLIB + FULVESTRANT	2
CABOZANTINIB	2
HCX	2
NIRAPARIB	2
CAPECITABINE + RT	2
PEMBROLIZUMAB + PEMETREXED	2
METHOTREXATE	2
GEFITINIB	2
OSIMERTINIB	2
ERLOTINIB	2
RIBOCICLIB	2
FLUOROURACIL + MITOMYCIN + RT	2
OLAPARIB	2
SORAFENIB	2
VINBLASTINE	2
AXITINIB	2
ALECTINIB	2
CAPECITABINE + CISPLATIN + RT	2
CISPLATIN + FLUOROURACIL + RT	2
TIVOZANIB	2
CRIZOTINIB	2
CAPECITABINE + MITOMYCIN	2
CAPECITABINE + VINORELBINE	2
DABRAFENIB	2
ABEMACICLIB	2
LENVATINIB	2
LETROZOLE + RIBOCICLIB	2
CAPECITABINE + MITOMYCIN + RT	2
BEXAROTENE	2
MITOMYCIN	2
INTERFERON	2
PONATINIB	2
VINCRISTINE	2
BUSULPHAN	2
CERITINIB	2
CAPECITABINE + TRASTUZUMAB	2
FLUOROURACIL + MITOMYCIN	2
NINTEDANIB	2
FLUOROURACIL + RT	2
REGORAFENIB	2
ANASTROZOLE + PALBOCICLIB	2
CETUXIMAB + CISPLATIN + FU	2
EVEROLIMUS + LENVATINIB	2
BRIGATINIB	2
LORLATINIB	2
CETUXIMAB + MDG	2
DICE TRIAL	2
BEVACIZUMAB + CAPECITABINE	2
TRASTUZUMAB + VINORELBINE	2
Fulvestrant + Palbociclib	2
Abemaciclib + Letrozole	2
ACELARATE TRIAL	2
VANDETANIB	2
EXEMESTANE + PALBOCICLIB	2
CISPLATIN + TRASTUZUMAB	2
INTERIM TRIAL	2
TRAMETINIB	2
UNIRAD TRIAL	2
MITOXANTRONE	2
CAPECITABINE + LAPATINIB	2
ICON9 TRIAL	2
ATEZOLIZUMAB + BEVACIZUMAB	2
COSMIC TRIAL	2
STAR TRIAL	2
RE-AKT TRIAL	2
BEACON TRIAL	2
DACOMITINIB	2
COLUMBUS TRIAL	2
PACKER MAINTENANCE	2
CAPECITABINE + NERATINIB	2
ANAGRELIDE + HYDROXYCARBAMIDE	2
INTERFERON BETA	2
CISPLATIN + FLUOROURACIL + TRASTUZUMAB	2
QUIZARTINIB	2
ASCIMINIB	2
PAKT TRIAL	2
METHOTREXATE + PEGASPARGASE	2
POLEM TRIAL	2
CISPLATIN + FLUORO + STREPTOZOCIN	2
CAP + RT	2
COMBI-AD TRIAL	2
OLYMPIA TRIAL	2
SPIRIT TRIAL	2
CEDIRANIB	2
FLUOROURACIL + INTERFERON ALFA	2
MILLENNIUM C16021 TRIAL	2
ENCORAFENIB	2
TALAZOPARIB	2
EVEROLIMUS + EXEMESTANE	2
CALYPSO TRIAL	2
INTERFERON ALFA + ZIDOVUDINE	2
IVOSIDENIB	2
SOLO1 TRIAL	2
LU-177	2
MITOMYCIN + RALTITREXED	2
NIMRAD TRIAL	2
SIROLIMUS	2
METHOTREXATE + TRASTUZUMAB	2
PALLET TRIAL	2
TOPARP TRIAL	2
TIPIFARNIB	2
RITUXIMAB + VINBLASTINE	2
IMATINIB + HYDROXYCARBAMIDE	2
METHOTREXATE + VINCRISTINE	2
BELLE3 TRIAL	2
GOSERELIN + PALBOCICLIB	2
PAZ02 TRIAL	2
PIN TRIAL	2
LAPATINIB	2
CASPS TRIAL	2
CISPLATIN + IMATINIB	2
TEMSIROLIMUS	2
LURBINECTIDIN	2
ERDAFITINIB	2
TIMELY TRIAL	2
COBIMETINIB	2
MASITINIB	2
BINIMETINIB	2
CREATE TRIAL	2
IRINOTECAN + MDG	3
OXALIPLATIN + MDG	3
CAPECITABINE + OXALIPLATIN	3
CETUXIMAB + IRINOTECAN + MDG	3
FLUOROURACIL + IRINOTECAN + OXALIPLATIN	3
CVD	3
RITUXIMAB	3
BORTEZOMIB + THALIDOMIDE	3
DARATUMUMAB	3
LENALIDOMIDE	3
IXAZOMIB + LENALIDOMIDE	3
BORTEZOMIB + DARATUMUMAB	3
IMATINIB	3
IBRUTINIB	3
CVP R	3
BORTEZOMIB	3
CISPLATIN + VINORELBINE	3
CABAZITAXEL	3
GEMCITABINE	3
CISPLATIN + ETOPOSIDE	3
MPV	3
TEMOZOLOMIDE	3
TRASTUZUMAB EMTANSINE	3
CETUXIMAB + OXALIPLATIN + MDG	3
CTD	3
OXALIPLATIN + MDG + PANITUMUMAB	3
ECX	3
RUXOLITINIB	3
CARFILZOMIB	3
IRINOTECAN + MDG + PANITUMUMAB	3
POMALIDOMIDE	3
IRINOTECAN + PANITUMUMAB + MDG	3
NILOTINIB	3
CAPECITABINE + CISPLATIN	3
CARDAMON TRIAL	3
BORTEZOMIB + PANOBINOSTAT	3
CYCLOPHOSPHAMIDE + LENALIDOMIDE	3
CVP + OBINUTUZUMAB	3
DASATINIB	3
TEMOZOLOMIDE + RT	3
MUK 9 TRIAL	3
IRINOTECAN	3
CYCLOPHOSPHAMIDE + RITUXIMAB + VINCRISTINE	3
VENETOCLAX	3
CYCLOPHOSPHAMIDE + RITUXIMAB	3
CARBO + FLUOROURACIL	3
CISPLATIN + GEMCITABINE + RITUXIMAB	3
OXALIPLATIN + RALTITREXED	3
CISPLATIN + PEMBROLIZUMAB + PEMETREXED	3
PEG INTERFERON	3
BOSUTINIB	3
OBINUTUZUMAB	3
CAPECITABINE + CETUXIMAB + IRINOTECAN	3
MPT	3
CISPLATIN + ETOPOSIDE + RT	3
IXAZOMIB	3
THALIDOMIDE	3
ETOPOSIDE	3
CAPECITABINE + TEMOZOLOMIDE	3
IRINOTECAN + TEMOZOLOMIDE	3
MERCAPTOPURINE + METHOTREXATE	3
BINIMETINIB + ENCORAFENIB	3
GEMCITABINE + OXALIPLATIN	3
BLINATUMOMAB	3
CMF	3
BEVACIZUMAB + OXALIPLATIN + MdG	3
BEVACIZUMAB + IRINOTECAN + MdG	3
CONCEPT TRIAL	3
OLARATUMAB	3
RALTITREXED	3
CETUXIMAB + CISPLATIN + FLUOROURACIL	3
GEMCITABINE + RT	3
IDELALISIB	3
BEVACIZUMAB + IRINOTECAN	3
BEVACIZUMAB + IRINOTECAN + MDG	3
IRINOTECAN + TEMOZOLOMIDE + VINCRISTINE	3
BOSTON TRIAL	3
CAPE + OXALIPLATIN + TRASTUZUMAB	3
VEMURAFENIB	3
IPM	3
CYCLOPHOSPHAMIDE + ETOPOSIDE	3
PENTOSTATIN	3
IPO	3
CYCLOPHOSPHAMIDE + MTX	3
PANOBINOSTAT	3
OXALIPLATIN	3
IRINOTECAN + BEVACIZUMAB	3
MITOTANE	3
FOCUS4 TRIAL	3
CYCLOPHOSPHAMIDE + VINCRISTINE	3
CAPECITABINE + CETUXIMAB + OXALIPATIN	3
CETUXIMAB + IRINOTECAN	3
COMPLEMENT TRIAL	3
AFLIBERCEPT + FU + IRINOTECAN	3
BEVACIZUMAB + CAPE + OXALIPLATIN	3
CVD + THALIDOMIDE	3
BEVACIZUMAB + PACLITAXEL	3
IRINOTECAN + RALTITREXED	3
IRINOTECAN + VINCRISTINE	3
CARFILZOMIB + CYCLOPHOSPHAMIDE	3
CLARITY TRIAL	3
BEVACIZUMAB + IRINOTECAN + OXALIPLATIN + MDG	3
MMM	3
LENALIDOMIDE + RITUXIMAB	3
CAPECITABINE + OXALIPLATIN + RT	3
PACLITAXEL + BEVACIZUMAB	3
BEVACIZUMAB + CAPE + IRINOTECAN	3
SHAMASH LBCMVD-56	3
PRIMUS 002 TRIAL	3
CYCLO + RITUXIMAB + VINCRISTINE	3
FLUOROURACIL + IRINOTECAN	3
MERCAPTOPURINE	3
PLATO TRIAL	3
CAPITAL TRIAL	3
BALLAD TRIAL	3
PACLITAXEL + RAMUCIRUMAB	3
DARATUMUMAB + LENALIDOMIDE	3
NEPTUNE TRIAL	3
FLUOROURACIL + OXALIPLATIN + TRASTUZUMAB	3
CAPECITABINE + CISPLATIN + TRASTUZUMAB	3
CYCLOPHOSPHAMIDE + FLUOROURACIL + METHOTREXATE	3
METHOTREXATE + RITUXIMAB	3
PREDNISOLONE + VINBLASTINE + RITUXIMAB	3
COMPLEMENT-1 TRIAL	3
BEVACIZUMAB + OXALIPLATIN	3
CAPECITABINE + IRINOTECAN + PANITUMUMAB	3
IBRUTINIB + VENETOCLAX	3
CCISV	3
SOPHIA TRIAL	3
BORTEZOMIB + VENETOCLAX	3
TREOSULFAN	3
DEPATUXIZUMAB MAFODOTIN + TEMOZOLOMIDE	3
DARATUMUMAB + POMALIDOMIDE	3
RCD TRIAL	3
OFATUMUMAB	3
CAPECITABINE + CETUXIMAB + OXALIPLATIN	3
CAPECITABINE + OXALIPLATIN + PANITUMUMAB	3
CISPLATIN + TEYSUNO	3
GEMCITABINE + OXALIPLATIN + PACLITAXEL	3
BEVACIZUMAB + TEMOZOLOMIDE	3
BORTEZOMIB + LENALIDOMIDE	3
TEYSUNO	3
ELOTUZUMAB + LENALIDOMIDE	3
BORTEZOMIB + PREDNISOLONE + MELPHALAN	3
ALEMTUZUMAB + CYCLOPHOSPHAMIDE + METHOTREXATE	3
MERCAPTOPURINE + METHOTREXATE + TRETINOIN	3
METHOTREXATE + PROCARBAZINE	3
CNS EPEN2007	3
CNS SIOP 2009	3
IRINOTECAN + MITOMYCIN	3
CYCLOPHOSPHAMIDE + GEMCITABINE + VINCRISTINE	3
LENALIDOMIDE + DARATUMUMAB	3
CAPE + CETUXIMAB + OXALIPLATIN	3
Cisplatin + Irinotecan	3
IXAZOMIB + THALIDOMIDE	3
CARFILZOMIB + POMALIDOMIDE	3
CYCLOPHOSHAMIDE + RITUXIMAB	3
ALEMTUZUMAB + ETOPOSIDE	3
SELINEXOR	3
STREPTOZOCIN	3
MCX	3
BENDAMUSTINE + BORTEZOMIB	3
GEMCITABINE + TEGAFUR	3
PATHOS TRIAL	3
CAPECITABINE + OXALIPLATIN + TRASTUZUMAB	3
CETUXIMAB + IRINOTECAN + RALTITREXED	3
IRINOTECAN + OXALIPLATIN + PACLITAXEL	3
PROMISE-MESO TRIAL	3
SCOPE 1 TRIAL	3
CYCLOPHOSPHAMIDE + RITUXIMAB + VINBLASTINE	3
MERCAPTOPURINE + VINBLASTINE	3
BEVACIZUMAB + IRINOTECAN + TEMOZOLOMIDE	3
CETUXIMAB + OXALIPLATIN	3
ACALABRUTINIB	3
BORTEZOMIB + RITUXIMAB	3
CYCLET	3
IBRUTINIB + RITUXIMAB	3
ABC-08 TRIAL	3
CNS HR MBL Cyclo/Vinc	3
FLUOROURACIL + GEMCITABINE	3
MANTA TRIAL	3
CETUXIMAB + OXALIPLATIN + RALTITREXED	3
VIM TRIAL	3
BEVACIZUMAB + OXALIPLATIN + MDG	3
CYCLOPHOSPHAMIDE + THALIDOMIDE + Dexamethasone	3
IRINOTECAN + OXALIPLATIN + PACLITAXEL	3
IRINOTECAN + PANITUMUMAB + RALTITREXED	3
IRINOTECAN + TALAZOPARIB	3
GELOX	3
PACRITINIB	3
PHITT D3 Vincristine / irinotecan	3
MELPHALAN + THALIDOMIDE	3
MM	3
SHINE TRIAL	3
VE	3
AFLIBERCEPT + CAPECITABINE + IRINOTECAN	3
CLL210 TRIAL	3
CISPLATIN + CYCLOPHOSPHAMIDE + ETOPOSIDE	3
CISPLATIN + RALTITREXED + TRASTUZUMAB	3
AFLIBERCEPT + FLUOROURACIL + IRINOTECAN	3
CISPLATIN + CYCLOPHOSPHAMIDE + VINCRISTINE	3
CYCLOPHOSPHAMIDE + TEMOZOLOMIDE	3
IRINOTECAN + TEMOZOLOMIDE + DINUTUXIMAB BETA	3
CARFILZOMIB + CYCLOPHOSPHAMIDE + LENALIDOMIDE	3
PACIFICO TRIAL	3
CYCLOPHOSPHAMIDE + IXAZOMIB	3
LIPOSOMAL DOXORUBICIN + PACLITAXEL	3
Ofatumumab	3
RITUXIMAB + TEMOZOLOMIDE	3
PACLITAXEL	4
CARBOPLATIN + PACLITAXEL	4
CHOP R	4
DOCETAXEL	4
GEMCARBO	4
CARBOPLATIN + ETOPOSIDE	4
EC	4
UKALL2011	4
DOCETAXEL + PERTUZUMAB + TRASTUZUMAB	4
ABVD	4
FEC + DOCETAXEL	4
BENDAMUSTINE + RITUXIMAB	4
CARBOPLATIN	4
CARBOPLATIN + PEMETREXED	4
DOCETAXEL + FLUOROURACIL + OXALIPLATIN	4
ERIBULIN	4
FEC 100	4
CARBOPLATIN + LIPOSOMAL DOX	4
CARBOPLATIN + DOCETAXEL + PERTUZUMAB + TRASTUZUMAB	4
CARBOPLATIN + VINORELBINE	4
EOX	4
CARBOPLATIN + PEMBROLIZUMAB + PEMETREXED	4
FEC	4
FEC 60 OR 75	4
GEMCITABINE + NAB-PACLITAXEL	4
FEC 100 + DOCETAXEL	4
CYCLOPHOSPHAMIDE + DOCETAXEL	4
CHLORAMBUCIL + OBINUTUZUMAB	4
BEVACIZUMAB + CARBOPLATIN + PACLITAXEL	4
CHOP	4
PACLITAXEL + TRASTUZUMAB	4
CAPECITABINE + EPIRUBICIN + OXALIPLATIN	4
PARTNER TRIAL	4
EC + PACLITAXEL	4
CARBOPLATIN + PACLITAXEL + RT	4
PCV	4
CISPLATIN + VINORELBINE + RT	4
BEVACIZUMAB + CARBO + PACLITAXEL	4
FEC + DOCETAXEL + PERTUZUMAB + TRASTUZUMAB	4
R-EPOCH	4
EPIRUBICIN	4
BENDAMUSTINE + OBINUTUZUMAB	4
STAMPEDE TRIAL	4
CYCLOPHOSPHAMIDE + DOCETAXEL + EPIRUBICIN	4
LIPOSOMAL DOXORUBICIN	4
CAV	4
EC + DOCETAXEL	4
CARBOPLATIN + DOCETAXEL	4
CYCLO + DOXORUBICIN + PACLITAXEL	4
ROSCO TRIAL	4
BEACOPP	4
CAPECITABINE + CARBOPLATIN	4
DOCETAXEL + NINTEDANIB	4
CARBOPLATIN + DOCETAXEL + TRASTUZUMAB	4
CISPLATIN + DOCETAXEL + FLUOROURACIL	4
MELPHALAN	4
CHLORAMBUCIL + RITUXIMAB	4
NAB-PACLITAXEL	4
MYELOMA XI TRIAL	4
DOXORUBICIN + IFOSFAMIDE	4
DTPACE	4
DOXORUBICIN + OLARATUMAB	4
ICON8B TRIAL	4
ICE-R	4
FEC + DOCETAXEL + TRASTUZUMAB	4
ESHAP	4
BRENTUXIMAB	4
ESCALATED BEACOP DAC	4
CHOP + OBINUTUZUMAB	4
TIP	4
DOCETAXEL + GEMCITABINE	4
TRABECTEDIN	4
CYCLOPHOSPHAMIDE + DOCETAXEL + DOXORUBICIN	4
EC + DOCETAXEL + PERTUZUMAB	4
RCEOP	4
ECF	4
DOXORUBICIN	4
VDT-PACE	4
TOPOTECAN	4
CHLVPP	4
BENDAMUSTINE	4
AML MYECHILD	4
CYCLOPHOSPHAMIDE + GEMCITABINE + RITUXIMAB + VINCRISTINE	4
CYCLOPHOSPHAMIDE + DOCETAXEL + TRASTUZUMAB	4
GDP	4
OEPA	4
CARBOPLATIN + CYCLOPHOSPHAMIDE + DOXORUBICIN + PACLITAX	4
ETOPOSIDE + IFOSFAMIDE	4
FEC + TRASTUZUMAB	4
PACLITAXEL + PERTUZUMAB + TRASTUZUMAB	4
GEMCITABINE + OXALIPLATIN + RITUXIMAB	4
DOCETAXEL + TRASTUZUMAB	4
EURO-EWING	4
CHLORAMBUCIL	4
ENRICH TRIAL	4
ATEZOLIZUMAB + BEVACIZUMAB + CARBOPLATIN + PACLITAXEL	4
AC	4
CHOEP	4
CARBOPLATIN + RT	4
CYCLOPHOSPHAMIDE + DOCETAXEL + DOXORUBICIN + PERTUZUM	4
CYCLOPHOSPHAMIDE + TOPOTECAN	4
VIDE	4
LOMUSTINE	4
ICE	4
CYCLOPHOSPHAMIDE + DOXORUBICIN + PERTUZUMAB + TRASTUZU	4
ESHAP R	4
CYCLOPHOSHAMIDE	4
IVE	4
GEMCITABINE + PACLITAXEL	4
RITUXIMAB + VENETOCLAX	4
ECARBOX	4
RMS 2005 TRIAL	4
CARBOPLATIN + VINORELBINE + RT	4
CYCLOPHOSPHAMIDE + ETOPOSIDE + PROCARBAZINE	4
CLADRIBINE	4
BENDAMUSTINE + CYTARABINE + RITUXIMAB	4
PVD	4
IDELALISIB + RITUXIMAB	4
IFOSFAMIDE	4
SCALOP-2 TRIAL	4
FEC + PACLITAXEL	4
CARBOPLATIN + CYCLOPHOSPHAMIDE + EPIRUBICIN + PACLITAXEL	4
CAPECITABINE + CISPLATIN + EPIRUBICIN	4
VIP	4
MIFAMURTIDE	4
ATEZOLIZUMAB + NAB PACLITAXEL	4
DHAP - R	4
IVA	4
ATEZOLIZUMAB + CARBOPLATIN + ETOPOSIDE	4
CISPLATIN + DOXORUBICIN	4
FEC 100 + TRASTUZUMAB	4
MAP	4
VEPEMB	4
IGEV	4
CARBOPLATIN + CYCLOPHOSPHAMIDE + EPIRUBICIN + PACLITAXEL	4
EP	4
BENDAMUSTINE + THALIDOMIDE	4
BORTEZOMIB + PANOBINOSTAT + THALIDOMIDE	4
FEC 100 + DOCETAXEL + TRASTUZUMAB	4
CARBOPLATIN + VINCRISTINE	4
FCARBOST	4
CYTARABINE + METHOTREXATE + RITUXIMAB + THIOTEPA	4
CAP	4
DACARBAZINE + DOXORUBICIN + VINBLASTINE	4
CARBOPLATIN + CETUXIMAB + FLUOROURACIL	4
CYCLO + DOXORUBICIN + VINCRISTINE	4
LIPOSOMAL DAUNORUBICIN + CYTARABINE (Vyxeos)	4
EMA/CO	4
CYCLO + DOCETAXEL + TRASTUZUMAB	4
PMITCEBO-R	4
CARBOPLATIN + EPIRUBICIN + PACLITAXEL	4
GDP R	4
CISPLATIN + PACLITAXEL	4
CYCLOPHOSPHAMIDE + DOCETAXEL + PERTUZUMAB + TRASTUZUMA	4
CVD R	4
EOF	4
NHL 2003	4
CAPECITABINE + STREPTOZOCIN	4
CYCLOPHOSPHAMIDE + ETOPOSIDE + RITUXIMAB + VINCRISTINE	4
ACTICCA-1 TRIAL	4
DACTINOMYCIN + ETOPOSIDE	4
CVP	4
INTERLACE TRIAL	4
MVAC	4
ICON TRIAL	4
IVE-R	4
PLATFORM TRIAL	4
MUK8 TRIAL	4
CARBO + DOCETAXEL + TRASTUZUMAB	4
DACARBAZINE	4
LOMUSTINE + PROCARBAZINE + VINCRISTINE	4
EDP	4
VAI	4
CAPECITABINE + CARBOPLATIN + EPIRUBICIN	4
DOXORUBICIN EMBOLISATION	4
LUTETIUM-177	4
DHAP	4
BEACOP DAC	4
CARBOPLATIN + CETUXIMAB + FU	4
CHLORAMBUCIL + ETOPOSIDE + LOMUSTINE	4
R-CHOEP	4
METHOTREXATE + VINBLASTINE	4
CNS EPENDYMOMA II	4
COMPARE TRIAL	4
CYCLOPHOSPHAMIDE + DOXORUBICIN	4
POMB	4
CARBOPLATIN + NAB-PACLITAXEL	4
CYCLOPHOSPHAMIDE + DOCETAXEL + DOXORUBICIN + TRASTUZUM	4
CISPLATIN + FU + TRASTUZUMAB	4
ESPAC TRIAL	4
EDP + MITOTANE	4
PROCAID TRIAL	4
TAC	4
CYCLOPHOSPHAMIDE + ETOPOSIDE + LOMUSTINE	4
SCOPE2 TRIAL	4
IVAC	4
ABVD R	4
CISPLATIN + DOX + ETOPOSIDE	4
ACCEPT TRIAL	4
VEDEX	4
IVADO	4
CISPLATIN + ETOPOSIDE + IFOSFAMIDE	4
CEV	4
CISPLATIN + IFOSFAMIDE + PACLITAXEL	4
INTERAACT TRIAL	4
CVP R + GEMCITABINE	4
CYCLOPHOSPHAMIDE + CYTARABINE + MERCAPTOPURINE	4
CYCLOPHOSPHAMIDE + EPIRUBICIN + FLUOROURACIL + PERTUZUM	4
GEMCITABINE + TIP	4
CISPLATIN + LOMUSTINE + VINCRISTINE	4
CAPECITABINE + CARBOPLATIN + TRASTUZUMAB	4
BORTEZOMIB + MELPHALAN	4
LOMUSTINE + PROCARBAZINE	4
GEMCITABINE + IFOSFAMIDE + VINORELBINE	4
CARMUSTINE + THIOTEPA	4
R-GEM-P	4
DACTINOMYCIN + VINCRISTINE	4
SPIRE TRIAL	4
DACARBAZINE + GEMCITABINE	4
CYCLOPHOSPHAMIDE + ETOPOSIDE + RITUXIMAB	4
HD EuroNet-PHL-C2 DECOPDAC	4
ALEMTUZUMAB + CYCLOPHOSPHAMIDE	4
DINUTUXIMAB BETA	4
PHITT TRIAL	4
CISPLATIN + ETOPOSIDE + PACLITAXEL	4
CISPLATIN + TOPOTECAN	4
PIXANTRONE	4
CYCLOPHOSPHAMIDE + VINORELBINE	4
DACTINOMYCIN	4
ETOPOSIDE + IFOSFAMIDE + RITUXIMAB	4
IGEV R	4
EURAMOS TRIAL	4
PAD	4
FOXFIRE TRIAL	4
CAPECITABINE + CARBOPLATIN + RT	4
CHLORAMBUCIL + OFATUMUMAB	4
GEMCITABINE + TREOSULPHAN	4
ROMICAR TRIAL	4
NEO-AEGIS TRIAL	4
STREPTOZOCIN + MDG	4
CARBOPLATIN + TOPOTECAN	4
CAPECITABINE + CISPLATIN + STREPTOZOCIN	4
COPP + DACARBAZINE	4
FEC 60 OR 75 + DOCETAXEL	4
CISPLATIN + EPIRUBICIN + FLUOROURACIL	4
CMV	4
CYTARABINE + OXALIPLATIN + RITUXIMAB	4
CYCLO + ETOPOSIDE + VINCRISTINE	4
HD EURONET-PHL	4
FCIST	4
MUK7 TRIAL	4
BENDAMUSTINE + GEMCITABINE + VINORELBINE	4
RATHL TRIAL	4
METHOTREXATE + VINORELBINE	4
R-CHOP / R-DHAP	4
HEPATOBLASTOMA	4
CARBO + LIPOSOMAL DOX + PACLITAXEL	4
CLADRIBINE + RITUXIMAB	4
BLEOMYCIN	4
VDC	4
CHLORAMBUCIL + DEXAMETHASONE + ETOPOSIDE	4
CISPLATIN + EPIRUBICIN	4
EC + PACLITAXEL + PERTUZUMAB	4
EP/EMA	4
CARBOPLATIN + DOCETAXEL + FLUOROURACIL	4
GEMCITABINE + VINORELBINE	4
MUK 12 TRIAL	4
BENDAMUSTINE + BRENTUXIMAB	4
DACARBAZINE + DOXORUBICIN + VINCRISTINE	4
CYCLOPHOSPHAMIDE + DOXORUBICIN + RITUXIMAB	4
CYCLOPHOSPHAMIDE + DOXORUBICIN + ETOPOSIDE + VINCRISTINE	4
ECARBOF	4
CHLORAMBUCIL + LOMUSTINE	4
EPIRUBICIN + FLUOROURACIL + OXALIPLATIN	4
CYCLO + ETOPOSIDE + NELARABINE	4
PEI	4
WILMS REL ICE	4
CISPLATIN + IFOSFAMIDE + MESNA + VINBLASTINE	4
CARBOPLATIN + EPIRUBICIN + FLUOROURACIL	4
CYCLOPHOSPHAMIDE + ETOPOSIDE + PROCARBAZINE + RITUXIMAB	4
BORTEZOMIB + CISPLATIN + CYCLOPHOSPHAMIDE + DOXORUBICIN	4
PENTOSTATIN + RITUXIMAB	4
CISPLATIN + FLUOROURACIL + STREPTOZOCIN	4
BEVACIZUMAB + CISPLATIN + PACLITAXEL	4
CHLVPP/EVA	4
CARBOPLATIN + DOXORUBICIN	4
PMITCEBO	4
HD EuroNet-PHL-C2 COPDAC	4
CHOP + INTERFERON ALFA + ZIDOVUDINE	4
CISPLATIN + DOCETAXEL	4
GEMCITABINE + RITUXIMAB	4
CVD (NEUROENDOCRINE)	4
CYTARABINE + ETOPOSIDE + TRETINOIN	4
DACARBAZINE + DOXORUBICIN	4
BLEOMYCIN + CISPLATIN + VINCRISTINE	4
FEC + PACLITAXEL + TRASTUZUMAB	4
PROCARBAZINE	4
FLUDARABINE	4
BORTEZOMIB + DOXORUBICIN	4
BLEOMYCIN + CISPLATIN + VINBLASTINE	4
CYCLOPHOSPHAMIDE + DACARBAZINE + VINCRISTINE	4
ETOPOSIDE + RITUXIMAB	4
HD EuroNet-PHL-C2 OEPA	4
CARFILZOMIB + LENALIDOMIDE	4
CISPLATIN + LIPOSOMAL DOXORUBICIN	4
EPSSG	4
CARBOPLATIN + PACLITAXEL + TRASTUZUMAB	4
SELPAC TRIAL	4
CHLORAMBUCIL + VINBLASTINE	4
CARBO + ETOPOSIDE + VINCRISTINE	4
NEOBLADE TRIAL	4
BOP	4
CISPLATIN + DACARBAZINE + VINBLASTINE	4
CYCLOPHOSPHAMIDE + DACTINOMYCIN + VINCRISTINE	4
ETOPOSIDE + LOMUSTINE + VINCRISTINE	4
CYCLO + FLUDARABINE + RITUXIMAB	4
INCA TRIAL	4
METAPHER TRIAL	4
CARBOPLATIN + EPIRUBICIN	4
CARBO + GEMCITABINE + RITUXIMAB	4
CAPE + CARBOPLATIN + CETUXIMAB	4
CARBO + THIOTEPA + TOPOTECAN	4
CARBOPLATIN + FLUOROURACIL + STREPTOZOCIN	4
BEVACIZUMAB + CARBO + GEMCITABINE	4
CARBOPLATIN + ETOPOSIDE + IFOSFAMIDE	4
CISPLATIN + DOXORUBICIN + ETOPOSIDE	4
CARBOPLATIN + CYCLOPHOSPHAMIDE + ETOPOSIDE	4
DACTINOMYCIN + ETOPOSIDE + METHOTREXATE	4
DACTINOMYCIN + CYCLO + VINCRISTINE	4
MUK6 TRIAL	4
POUT TRIAL	4
Bortezomib + DA-EPOCH-R	4
CISPLATIN + CYCLO + DOXORUBICIN	4
DA-EPOCH + BORTEZOMIB	4
RPACE-BOM	4
CYTARABINE + ETOPOSIDE	4
CAPECITABINE + DOCETAXEL	4
EPIRUBICIN + OXALIPLATIN	4
CHOP R + BORTEZOMIB	4
CYCLOPHOSPHAMIDE + CYTARABINE + MERCAPTOPURINE + METHO	4
CARBOPLATIN + NAB-PACLITAXEL + TRASTUZUMAB	4
EC + DOCETAXEL + TRASTUZUMAB	4
IFOSFAMIDE + PACLITAXEL	4
NAB-PACLITAXEL + TRASTUZUMAB	4
AC + DOCETAXEL + TRASTUZUMAB	4
GEMCITABINE + RITUXIMAB + VINORELBINE	4
CARBOPLATIN + EPIRUBICIN + ETOPOSIDE + MELPHALAN	4
DACTINOMYCIN + DOXORUBICIN + VINCRISTINE	4
LOMUSTINE + TEMOZOLOMIDE	4
CARBOPLATIN + ETOPOSIDE + VINCRISTINE	4
CYCLOPHOSPHAMIDE + PACLITAXEL	4
mEOC TRIAL	4
CHOP-R + CYTARABINE	4
CISPLATIN + DOXORUBICIN + METHOTREXATE	4
CISPLATIN + DOX + ETOPOSIDE + MITOTANE	4
OPTIMAL TRIAL	4
CARBOPLATIN + IFOSFAMIDE	4
CYCLOPHOSPHAMIDE + DOXORUBICIN + ETOPOSIDE	4
CYCLOPHOSPHAMIDE + FLUOROURACIL + METHOTREXATE + Trastuz	4
PETREA TRIAL	4
G-CVP	4
CISPLATIN + DACTINOMYCIN + ETOPOSIDE + METHOTREXATE	4
CARBOPLATIN + TRASTUZUMAB	4
CVAD	4
CYCLOPHOSPHAMIDE + DOXORUBICIN + METHOTREXATE + VINCRIS	4
CYCLOPHOSPHAMIDE + FLUDARABINE + THIOTEPA	4
LOPP	4
BUSULPHAN + CYCLO	4
CHLORAMBUCIL + DOXORUBICIN + ETOPOSIDE + PROCARBAZINE +	4
ETOPOSIDE + IFOSFAMIDE + RITUXIMAB + THIOTEPA	4
TIDE	4
MIC	4
PANTHER TRIAL	4
MELPHALAN + TOPOTECAN	4
CARBOPLATIN + ETOPOSIDE + PACLITAXEL	4
CYCLOPHOSPHAMIDE + DOXORUBICIN + PROCARBAZINE + VINCRIST	4
MITOXANTRONE + TRETINOIN	4
RETINOBLASTOMA	4
WT Relapse Carbo/Cyclo/Etop	4
NEMO TRIAL	4
BEVACIZUMAB + LOMUSTINE	4
CISPLATIN + NAB-PACLITAXEL	4
CARBOPLATIN + RALTITREXED	4
QUASAR 2 TRIAL	4
CISPLATIN + GEMCITABINE + PACLITAXEL	4
CYCLOPHOSPHAMIDE + METHOTREXATE	4
Z-DEX	4
PACKER INDUCTION VINCRISTINE + RT	4
BEVACIZUMAB + CARBOPLATIN + NAB-PACLITAXEL	4
DOXORUBICIN + GEMCITABINE	4
OCTOPUS TRIAL	4
ARISTOTLE TRIAL	4
CARBOPLATIN + EPIRUBICIN + METHOTREXATE + VINBLASTINE	4
CARBOPLATIN + FLUOROURACIL + TRASTUZUMAB	4
CISPLATIN + CYCLOPHOSPHAMIDE + DOXORUBICIN	4
DOXORUBICIN + STEPTOZOTOCIN	4
CYCLOPHOSPHAMIDE + ETOPOSIDE + LOMUSTINE + RITUXIMAB	4
RIALTO TRIAL	4
STS Non- Rhabdo EpSSG Vinblastine/MTX	4
ICE TRIAL	4
MVCARBO	4
CABAZITAXEL + CARBOPLATIN	4
CHOP + BORTEZOMIB	4
GDCVP	4
EPIRUBICIN + FLUOROURACIL	4
IDARUBICIN + MERCAPTOPURINE + METHOTREXATE + VINCRISTINE	4
PMITCEBO R	4
CYCLOPHOSPHAMIDE + DOXORUBICIN + PACLITAXEL	4
ARIEL 4 TRIAL	4
CISPLATIN + DACARBAZINE	4
BLEOMYCIN + OXALIPLATIN + VINCRISTINE	4
CNS Germ Cell Tumour II - Non Germinomatous - Standard PEI	4
ETOPOSIDE + IFOSFAMIDE + METHOTREXATE	4
METHOTREXATE + PROCARBAZINE + RITUXIMAB	4
CARBOPLATIN + DACARBAZINE	4
CNS Medbl St Risk <3yrs induction	4
CYCLOPHOSPHAMIDE + IDARUBICIN	4
ReBEL TRIAL	4
ACE	4
BLEOMYCIN + VINCRISTINE	4
CHLVPP/PABLOE	4
CYTARABINE + OXALIPLATIN	4
PHITT D Carboplatin/Doxorubicin	4
BLEOMYCIN + CYCLOPHOSPHAMIDE + ETOPOSIDE + GEMCITABINE +	4
BLEOMYCIN + CYCLOPHOSPHAMIDE + ETOPOSIDE + METHOTREXAT	4
DOXORUBICIN + PEGASPARGASE + VINCRISTINE	4
PAC-E	4
SIOP	4
BENDAMUSTINE + GEMCITABINE + RITUXIMAB + VINORELBINE	4
CYCLOPHOSPHAMIDE + CYTARABINE + POMALIDOMIDE	4
PHITT C CDDP-M	4
ADOC	4
CARBOPLATIN + DOXORUBICIN + MITOMYCIN	4
CARBOPLATIN + ETOPOSIDE + METHOTREXATE	4
CARBOPLATIN + THIOTEPA + TOPOTECAN	4
CYCLOPHOSPHAMIDE + VINBLASTINE	4
HERBY TRIAL	4
HLH Induction W3-8 Etoposide/Dexamethasone/Ciclosporin	4
CARBOPLATIN + ETOPOSIDE + THIOTEPA	4
ERIBULIN + TRASTUZUMAB	4
ALEMTUZUMAB + ETOPOSIDE + METHOTREXATE	4
PHITT D2 Carboplatin / etoposide	4
VIOLA TRIAL	4
BEVACIZUMAB + CARBO + DOCETAXEL	4
CISPLATIN + DOXORUBICIN + IFOSFAMIDE	4
BORTEZOMIB + POMALIDOMIDE	4
EW Rel rEECur Ifosfamide	4
ALEMTUZUMAB + FLUDARABINE + THIOTEPA + TREOSULFAN	4
ALEMTUZUMAB + FLUDARABINE + TREOSULFAN	4
BLEOMYCIN + CISPLATIN + ETOPOSIDE + PACLITAXEL	4
CARBOPLATIN + CYCLO + DOXORUBICIN	4
CNS Ependymoma 99 vinc+cyclo+etop	4
CNS Medbl HR	4
CYCLOPHOSPHAMIDE + MITOXANTRONE + RITUXIMAB + VINCRISTINI	4
EPIRUBICIN + OXALIPLATIN + RALTITREXED	4
FLUOROURACIL + LOMUSTINE	4
PHITT C Carboplatin/Doxorubicin	4
PHITT E2 PLADO	4
WT2001	4
ASPARAGINASE + CYTARABINE + ETOPOSIDE + METHOTREXATE	4
BLEOMYCIN + CYCLOPHOSPHAMIDE + GEMCITABINE + RITUXIMAB +	4
CLADRIBINE + CYCLOPHOSPHAMIDE + RITUXIMAB	4
ESTRAMUSTINE	4
PACE-BOM	4
CARMUSTINE + CYCLOPHOSPHAMIDE + ETOPOSIDE	4
CHLORAMBUCIL + ETOPOSIDE + RITUXIMAB	4
CISPLATIN + CYCLOPHOSPHAMIDE	4
CYTARABINE + IDARUBICIN + METHOTREXATE + RITUXIMAB	4
DACTINOMYCIN + IFOSFAMIDE + VINCRISTINE	4
FMD	4
MAP + MIFAMURTIDE	4
MUK 11 TRIAL	4
CLOFARABINE + CYCLOPHOSPHAMIDE + ETOPOSIDE	4
CLOFARABINE	4
CYCLOPHOSPHAMIDE + DOXORUBICIN + METHOTREXATE + RITUXIM	4
ROVALPITUZUMAB TESIRINE	4
ALEMTUZUMAB + FLUDARABINE	4
CLADRIBINE + CYTARABINE + IDARUBICIN	4
HLH Consol Etop/Dexamthasone/Ciclosporin	4
CARBOPLATIN + PROCARBAZINE	4
CYTARABINE + FLUDARABINE + RITUXIMAB	4
CYTARABINE + IDARUBICIN	4
CYTARABINE + MERCAPTOPURINE + METHOTREXATE + PEGASPARA	4
HELIOS TRIAL	4
IBRITUMOMAB TIUXETAN + RITUXIMAB + Y90	4
Idarubicin + Tretinoin	4
CYCLOPHOSPHAMIDE + DOCETAXEL + EPIRUBICIN + TRASTUZUMAB	4
DOXORUBICIN + METHOTREXATE + MIFAMURTIDE	4
IDARAM	4
BLEOMYCIN + CYCLOPHOSPHAMIDE + ETOPOSIDE + GEMCITABINE +	4
CARBOPLATIN + TEYSUNO	4
CISPLATIN + DOXORUBICIN + METHOTREXATE + MIFAMURTIDE	4
CVP R + ETOPOSIDE	4
STS Non-Rhabdo EpSSG 2005 Ifos	4
STS Non-Rhabdo EpSSG 2005 VA	4
CARBOPLATIN + CYCLOPHOSPHAMIDE + EPIRUBICIN + FLUOROURA	4
CCEP	4
CYCLOPHOSPHAMIDE + NAB-PACLITAXEL	4
DENOSUMAB + ERIBULIN	4
IFOSFAMIDE + VINCRISTINE	4
CISPLATIN + CYTARABINE	4
CYCLOPHOSPHAMIDE + DACTINOMYCIN	4
CYCLOPHOSPHAMIDE + ETOPOSIDE + THALIDOMIDE	4
MEAD (PEG-ASPARAGINASE)	4
R-PCE2BO (ETOPOPHOS¿½)	4
BEVACIZUMAB + CARBO	4
CARBOPLATIN + CETUXIMAB	4
CARMUSTINE	4
GEMCITABINE + OXALIPLATIN + PEGASPARAGINASE	4
METHOTREXATE + MITOMYCIN + MITOXANTRONE	4
AZACITIDINE	5
BEP	5
UKALL2014	5
CYTARABINE	5
TRIFLURIDINE + TIPIRACIL	5
CYCLOPHOSPHAMIDE	5
DA	5
FLAG + IDARUBICIN	5
FCR	5
AML19 TRIAL	5
FLAIR TRIAL	5
R IVAC	5
BEAM	5
MATRIX TRIAL	5
ARSENIC TRIOXIDE + TRETINOIN	5
ALEMTUZUMAB + FLUDARABINE + MELPHALAN	5
AML18 TRIAL	5
R CODOX-M/ R IVAC	5
R CODOX M	5
CYTARABINE + DAUNORUBICIN + GEMTUZUMAB OZOGAMICIN	5
MYELOMA XII TRIAL	5
RADIUM 223	5
LEAM	5
METHOTREXATE HIGH DOSE	5
ALEMTUZUMAB	5
CHOP R + METHOTREXATE	5
CYTARABINE HIGH DOSE	5
CYCLOPHOSPHAMIDE + FLUDARABINE	5
UKALL60+	5
ATG + BUSULFAN + FLUDARABINE	5
CYCLOPHOSPHAMIDE HIGH DOSE	5
NORDIC	5
AML17 TRIAL	5
UKALLR3	5
FBC	5
ALL INTERFANT 06	5
ALL INTERIM GUIDELINES	5
Yescarta + Fludarabine + Cyclophosphamide	5
CARBOPLATIN + ETOPOSIDE + IFOSFAMIDE + RITUXIMAB	5
STS RHABDO RMS 2005	5
ALEMTUZUMAB + CYCLO + FLUDARABINE	5
EW REL REECUR	5
STS RHABDO VIT0910	5
ARSENIC TRIOXIDE	5
APML	5
UKALL2003	5
ATG + CYCLOPHOSPHAMIDE TBI ALLO	5
ARSENIC TRIOXIDE + ATRA	5
ALL INTENSIFICATION	5
FLAG	5
POMB/ACE	5
CYTARABINE + RITUXIMAB	5
INOTUZUMAB OZOGAMICIN	5
AML16 TRIAL	5
AML 18 PILOT TRIAL	5
SMILE	5
BLEOMYCIN + CARBOPLATIN + ETOPOSIDE	5
MIDOSTAURIN	5
ALL PH+VE	5
ALEMTUZUMAB + BUSULFAN + CYCLOPHOSPHAMIDE	5
BLEOMYCIN + CARBOPLATIN + CISPLATIN + VINCRISTINE	5
LACE	5
CODOX M	5
CYTARABINE HD + METHOTREXATE HD	5
FAB LMB	5
ALL Interim Guide 2019 Induction A	5
LI-1 TRIAL	5
IDARUBICIN	5
AMSACRINE + ATG+ BUSULFAN + CYTARABINE + FLUDARABINE	5
AMSACRINE + ATG + BUSULFAN + CYTARABINE + FLUDARABINE	5
Kymriah + Fludarabine + Cyclophosphamide	5
DOXORUBICIN + HD MTX	5
BEAM + ALEMTUZUMAB	5
DA-EPOCH	5
DAUNORUBICIN + CYTARABINE + MIDOSTAURIN	5
ALL Interim Guide 2019 Augmented BFM Consolidation C	5
FLUDARABINE + MELPHALAN	5
ALEMTUZUMAB + PENTOSTATIN	5
IFOSFAMIDE + LIPOSOMAL DOXORUBICIN	5
ATG	5
CHOP-R + CYTARABINE HIGH DOSE	5
ALL MAINTENANCE	5
CYCLOPHOSPHAMIDE + CYTARABINE + DOXORUBICIN + RITUXIMAB	5
CISPLATIN + DACTINOMYCIN + ETOPOSIDE + FILGRASTIM + METHOT	5
ALEMTUZUMAB + CYCLOPHOSPHAMIDE + FLUDARABINE	5
AML DOWNS	5
ALL Interim Guide 2019 Delayed Intensification A	5
ACE (GERM CELL)	5
IDARUBICIN + TRETINOIN	5
ALL Interim Guide 2019 Maintenance	5
ALL Interim Guide 2019 Standard BFM Consolidation B	5
UKALL 60+ TRIAL	5
ALL Interim Guide 2019 Standard Interim Maintenance A	5
ALL Interim Guide 2019 Induction B	5
DAUNORUBICIN + PEGASPARAGINASE + VINCRISTINE	5
AML INTERIM GUIDELINES	5
CYTARABINE + METHOTREXATE + RITUXIMAB	5
ALL INDUCTION	5
FIGARO TRIAL	5
CYTARABINE + ETOPOSIDE + IFOSFAMIDE + METHOTREXATE + RITU	5
TRANSPLANT BEAM	5
GEMTUZUMAB OZOGAMICIN	5
DAUNORUBICIN + VNCRISTINE	5
TRANSPLANT CYTARABINE + ETOPOSIDE + LOMUSTINE + MELPHALA	5
MACE	5
TRANSPLANT ALEMTUZUMAB + FLUDARABINE + MELPHALAN	5
NELARABINE	5
UKALL XII	5
ALL R T-CELL CONSOLIDATION	5
METHOTREXATE + RITUXIMAB + TEMOZOLOMIDE	5
ADE	5
TRANSPLANT CYCLOPHOSPHAMIDE + FLUDARABINE (+/- TBI)	5
CARBOPLATIN + CYCLOPHOSPHAMIDE + ETOPOSIDE + PACLITAXEL	5
ALL CONSOLIDATION	5
ETOPOSIDE + IFOSFAMIDE + MTX	5
ROMAZA TRIAL	5
ALL Int Guide B BFMCons	5
CLOFARABINE + CYTARABINE	5
CYTARABINE IT + METHOTREXATE	5
FLA	5
ALL Int Guide A Ind	5
CYTARABINE + METHOTREXATE	5
ALL Interim Guide 2019	5
IDARUBICIN + MITOXANTRONE + TRETINOIN	5
CYTARABINE + FLUDARABINE	5
AZACITIDINE + VENETOCLAX	5
ALL Int GdeC D Int PhIV reind PAsp	5
BENDAMUSTINE + CYTARABINE + ETOPOSIDE + MELPHALAN	5
CYTARABINE + ETOPOSIDE + MITOXANTRONE	5
BUSULFAN + FLUDARABINE	5
CODOX M + IVAC	5
ALEMTUZUMAB + LEAM	5
BUSULFAN + CYCLOPHOSPHAMIDE	5
CYTARABINE + MIDOSTAURIN	5
ERWINIA ASPARAGINASE	5
TRANSPLANT ALEMTUZUMAB + CYTARABINE + ETOPOSIDE + LOMUS	5
111 TRIAL	5
AMSACRINE + BUSULFAN + CYTARABINE + FLUDARABINE	5
ALL Interim Guide 2019 Consolidation A	5
FLUDARABINE + THIOTEPA + TREOSULFAN	5
AML Relapsed FLA - Idarubicin	5
TRIFLURIDINE TIPIRACIL	5
AZACITIDINE + RUXOLITINIB	5
BLEOMYCIN + CISPLATIN + ETOPOSIDE + METHOTREXATE + VINCRIS	5
ATG + BUSULFAN + FLUDARABINE + THIOTEPA	5
IMATINIB + MERCAPTOPURINE + VINCRISTINE	5
ATG + CYCLOPHOSPHAMIDE + FLUDARABINE	5
BUSULFAN + MELPHALAN	5
CANTATA TRIAL	5
BLEOMYCIN + CISPLATIN + ETOPOSIDE	5
ATG + CYCLOPHOSPHAMIDE	5
BLEOMYCIN + CYCLOPHOSPHAMIDE + ETOPOSIDE + GEMCITABINE +	5
CNS ATRT Doxorubicin/Ifosfamide/Carboplatin/Etoposide/Methotrexate	5
CYCLOPHOSPHAMIDE + CYTARABINE + DAUNORUBICIN + MERCAPT(	5
DOXORUBICIN + METHOTREXATE	5
ATG + FLUDARABINE	5
CAPECITABINE + LOMUSTINE	5
METHOTREXATE HIGH DOSE + RITUXIMAB	5
ALL Interim Guide 2019 Standard Interim Maintenance B	5
METHOTREXATE HD + VINCRISTINE	5
GILTERITINIB	5
BUSULFAN + CYCLOPHOSPHAMIDE + FLUDARABINE + THIOTEPA	5
CYTARABINE + Gemtuzumab Ozogamicin	5
MA	5
BEAM + FLUDARABINE	5
AMSACRINE + CYTARABINE	5
CYTARABINE + FLUDARABINE + IDARUBICIN	5
DAUNORUBICIN	5
TRANSPLANT ATG + FLUDARABINE + THIOTEPA + TREOSULFAN	5
GCLAC	5
BUSULFAN + FLUDARABINE + METHOTREXATE	5
IMATINIB + MERCAPTOPURINE + METHOTREXATE	5
ALL Int Guide B Int Maint	5
TRANSPLANT ALEMTUZUMAB + CYCLOPHOSPHAMIDE + FLUDARABIN	5
DOXORUBICIN + IFOSFAMIDE + VINCRISTINE	5
BUSULFAN + THIOTEPA	5
CLOFARABINE + CYTSARABINE + IDARUBICIN	5
ALL NOPHO	5
AMSACRINE + ATG + CYCLOPHOSPHAMIDE + CYTARABINE + FLUDAR	5
CISPLATIN + ETOPOSIDE + METHOTREXATE	5
ALL Interfant 06 ADE	5
ALEMTUZUMAB + FLUDARABINE + LEAM	5
ETOPOSIDE + MELPHALAN	5
IDARUBICIN + IMATINIB + VINCRISTINE	5
ALL Interim Guide 2019 Induction C	5
TRANSPLANT BUSULFAN + FLUDARABINE	5
ALL Interim Guide 2019 NOPHO C	5
CYCLOPHOSPHAMIDE + CYTARABINE + ETOPOSIDE + METHOTREXA	5
CLADRIBINE + CYTARABINE + DAUNORUBICIN	5
TRANSPLANT AMSACRINE + ANTITHYMOCYTE IMMUNOGLOBULIN (R	5
TRANSPLANT ATG + BUSULFAN + FLUDARABINE + THIOTEPA	5
CLADRIBINE + CYTARABINE	5
DE ANGELIS	5
FORODESINE	5
ATG + FLUDARABINE + MELPHALAN	5
ATG + FLUDARABINE + THIOTEPA + TREOSULFAN	5
TRANSPLANT FLUDARABINE + THIOTEPA + TREOSULFAN	5
CARBOPLATIN + CYTARABINE + ETOPOSIDE	5
CYCLOPHOSPHAMIDE + ETOPOSIDE + NELARABINE	5
DECITABINE	5
MIDAC	5
BLEOMYCIN + CYCLOPHOSPHAMIDE + ETOPOSIDE + GEMCITABINE +	5
BLEOMYCIN + CYCLOPHOSPHAMIDE + ETOPOSIDE + GEMCITABINE +	5
CYTARABINE + VINCRISTINE	5
IDARUBICIN + METHOTREXATE + VINCRISTINE	5
MTX HD + PROCARBAZINE + VINCRISTINE	5
BEAM R	5
BUSULFAN + CYCLOPHOSPHAMIDE + FLUDARABINE	5
CARBOPLATIN + CYTARABINE + RITUXIMAB	5
CYTARABINE + ETOPOSIDE + IMATINIB	5
CYTARABINE + ETOPOSIDE + METHOTREXATE	5
ALL/LBL T Cell Relapse Intensification	5
BLEOMYCIN + CYCLOPHOSPHAMIDE + ETOPOSIDE + MITOXANTRONI	5
CARBOPLATIN + ETOPOSIDE + IFOSFAMIDE + PACLITAXEL	5
CISPLATIN + CYCLOPHOSPHAMIDE + CYTARABINE + DOXORUBICIN +	5
DAUNORUBICIN + METHOTREXATE + PEGASPARAGINASE + VINCRIS	5
CNS HGG CISPLATIN/ETOPOSIDE	5
CYTARABINE + METHOTREXATE HD	5
ALL UKALL2011 TRIAL + ALL UKALL2011 Intensification MTX	5
TRANSPLANT ATG + ETOPOSIDE (+/- TBI)	5
ALL/LBL T Cell Relapse Consolidation	5
[illegible]	5
ALL INTENSIFICATION MTX	5
AML RELAPSED	5
CAMELLIA TRIAL	5
SPANISH MAINTENANCE	5
ALL/LBL T Cell Relapse NECTAR Cyclophosphamide, etoposide, nelarabine and IT	5
HORMONES	1
CYTARABINE + METHOTREXATE + PEGASPARGINASE	5
CHOP R + LIPOSOMAL DOXORUBICIN	3
CYCLOPHOSPHAMIDE + DACTINOMYCIN + DOXORUBICIN + VINCRISTINE	5
TRIAL	6
CYCLOPHOSPHAMIDE + FLUDARABINE + MITOXANTRONE	3
CYCLOPHOSPHAMIDE + LIPOSOMAL DOX	3
VELIPARIB	1
CARBOPLATIN + IRINOTECAN	3

Kerr S, et al. BMJ Open 2022; 12:e050994. doi: 10.1136/bmjopen-2021-050994
