# Supplementary material for: Common protocol for validation of the QCOVID algorithm across the four UK nations
Source: BMJ Open. 2022 Jun 14;12(6):e050994. doi: 10.1136/bmjopen-2021-050994 (PMC9198386; doi:10.1136/bmjopen-2021-050994)
Supplement: Author's manuscript [file bmjopen-2021-050994.draft_revisions.pdf]

BMJ Open is committed to open peer review. As part of this commitment we make the peer review history of every article we publish publicly available.

When an article is published we post the peer reviewers' comments and the authors' responses online. We also post the versions of the paper that were used during peer review. These are the versions that the peer review comments apply to.

The versions of the paper that follow are the versions that were submitted during the peer review process. They are not the versions of record or the final published versions. They should not be cited or distributed as the published version of this manuscript.

BMJ Open is an open access journal and the full, final, typeset and author-corrected version of record of the manuscript is available on our site with no access controls, subscription charges or pay-per-view fees (http://bmjopen.bmj.com).

If you have any questions on BMJ Open's open peer review process please email info.bmjopen@bmj.com

A common methodology for validation of the Qcovid algorithm across the four UK nations

Journal:	*BMJ Open*
Manuscript ID	bmjopen-2021-050994
Article Type:	Protocol
Date Submitted by the Author:	06-Mar-2021

Complete List of Authors:	Kerr, Steven; The University of Edinburgh Usher Institute of Population Health Sciences and Informatics,<br>Robertson, Chris; University of Strathclyde, Department of Mathematics and Statistics<br>Nafilyan, Vahe; Office for National Statistics<br>Lyons, Ronan; University of Wales Swansea, Swansea Clinical School<br>Kee, Frank; Queen's University Belfast, UKCRC Centre of Excellence for Public Health (NI)<br>Cardwell, Christopher; Queen's University Belfast, School of Medicine, Dentistry and Biomedical Sciences<br>Coupland, Carol; University of Nottingham, Division of Primary Care<br>Lyons, Jane; Swansea University Medical School<br>Humberstone, Ben; Office for National Statistics<br>Hippisley-Cox, Julia; University of Oxford, Nuffield Department of Primary Care Sciences<br>Sheikh, Aziz; University of Edinburgh, Division of Community Health Sciences

Keywords:	COVID-19, Epidemiology < INFECTIOUS DISEASES, Public health < INFECTIOUS DISEASES

SCHOLARONE™
Manuscripts

A common methodology for validation of the QCOVID algorithm across the four UK nations

Steven Kerr, stevenkerr2@gmail.com (corresponding author) [1]

Chris Robertson [2,3]

Vahe Nafilyan [4]

Ronan A Lyons [5]

Frank Kee [6]

Chris Cardwell [6]

Carol Coupland [7]

Jane Lyons [5]

Ben Humberstone [4]

Julie Hippisley-Cox [8]

Aziz Sheikh [1]

[1] Usher Institute, The University of Edinburgh, NINE Edinburgh BioQuarter, Edinburgh, UK, EH16 4UX.

[2] University of Strathclyde, Glasgow, UK.

[3] Public Health Scotland, UK

[4] Office for National Statistics, UK.

[5] Population Data Science, Swansea University Medical School, Swansea, UK.

[6] Queen's University Belfast, Belfast, UK.

[7] University of Nottingham, Nottingham, UK.

[8] University of Oxford, Oxford, UK.

Keywords: Covid-19, Coronavirus, QCOVID, Epidemiology, Public Health.

Word count: 3,094

For peer review only

ABSTRACT

Introduction:

The QCOVID algorithm is a risk prediction tool for infection and subsequent hospitalisation/death due to SARS-Cov-2. At the time of writing, it is being used in important policymaking decisions by the UK and devolved governments for combatting the Covid-19 pandemic, including deliberations on shielding and vaccine prioritisation. There are four statistical validations exercises currently planned for the QCOVID algorithm, using data pertaining to England, Northern Ireland, Scotland and Wales respectively. This paper presents a common procedure for conducting and reporting on validation exercises for the QCOVID algorithm.

Methods and Analysis:

We will use open, retrospective cohort studies to assess the performance of the QCOVID risk prediction tool in each of the four UK nations. Linked datasets comprising of primary and secondary care records, virological testing data and death registrations will be assembled in trusted research environments in England, Scotland, Northern Ireland and Wales. We will seek to have population level coverage as far as possible within each nation. The following performance metrics will be calculated by strata: Harrell's C, Brier score, $R^{2,}$ and Royston's D.

Ethics and dissemination:

Approvals have been obtained from relevant ethics bodies in each UK nation. Findings will be made available to national policymakers, presented at conferences and published in peer reviewed journal

Strengths and limitations of this study:

- We will use national level data within each UK nation
- There are potential issues with missing data and differences in the way data is recorded in each country.
- We will evaluate the performance of the algorithm according to several relevant metrics.

For peer review only

INTRODUCTION:

The QCOVID algorithm [1] has been developed to help identify adults at high risk of being hospitalised or dying following infection with SARS-Cov-2. The algorithm takes as input a total of 40 variables including age, sex, ethnicity, Townsend deprivation score [2] and housing category, as well as clinical information including body mass index (BMI) and 33 variables related to medical conditions and treatments. It outputs the predicted probability that an individual will be infected with SARS-Cov-2 and then hospitalised, and the predicted probability that an individual will be infected with SARS-Cov-2 and then die, over a 90 day period. The algorithm was trained using information from the QResearch database [3], which as of April 2020 contained routinely collected data from 1205 General Practices across England, covering 10.5 million patients. The initial training dataset comprised of a cohort of 6.08 million individuals tracked from the 24 January 2020 to 30 April 2020, and was validated on a subset of 2.17 million individuals tracked from 1 May 2020 to 30 June 2020. The research protocol for the development of the QCOVID algorithm can be found in [4].

The QCOVID algorithm was commissioned by the Chief Medical Officer for England on behalf of the UK government. The algorithm is currently being used to inform UK and devolved government policy on combatting the SARS-Cov-2 pandemic, including guidance on social-distancing and shielding measures, as well vaccine prioritisation. [5] It is therefore of great importance to validate the predictions of the algorithm in sub-populations of the UK that were not in the initial training set, but will potentially be subject to those policies.

Four separate validation exercises for the QCOVID algorithm are planned – one for each of England, Northern Ireland, Scotland and Wales. In order to facilitate useful comparison of the results of the separate validation exercises, it is necessary to establish a consistent set of procedures. The purpose of this paper is to explicate a common methodology for the validation of the QCOVID algorithm across the four nations of the UK.

METHODS AND ANALYSIS:

Study design:

For peer review only

Open, retrospective cohort study designs will be employed, making use of routinely collected data from General Practices as well as linked datasets on hospital admissions, reverse-transcription polymerase chain reaction (RT-PCR) testing for Covid-19, and registered deaths. We will aim to have national coverage as far as is possible within each of the four nations of the UK.

Data Sources:

Box 1 contains a brief summary of the main datasets that will be used in the validation exercise for each nation

Box 1: Main datasets to be used

For peer review only

England: Office for National Statistics (ONS) Public Health Linked Data Asset. This dataset is based on the 2011 Census in England covering 40.1 million people, linked at individual level using the NHS number to mortality records, Hospital Episode Statistics (HES) and the General Practice Extraction Service (GPES) data for pandemic planning and research. The data covers 80% of the population of England aged 19 and over.

Northern Ireland: National Health Application and Infrastructure Services (NHAIS) will be used for demographic information. The Patient Administration System (PAS) will be used for data on hospital admissions. Death data will be drawn from the Registrar General, and identified as Covid-19 related through the official Northern Ireland Statistics and Research Agency (NISRA) dashboard. The General Practice Information Platform (GPIP) will bring together GP records from practices across Northern Ireland into a single dataset for use in the validation. As this is not held in the Honest Broker Service, a separate request to its governance board is being made. The Electronic Prescribing Database (EPD) will be used to access information on prescriptions.

Scotland: EAVE II dataset [6]. Contains primary health care records for 5.4 million people covering 99% of the population of Scotland, linked with secondary care data from Scottish Morbidity Record (SMR), Covid-19 test results from Electronic Communication of Surveilance Scotland (ECOSS), and mortality data from National Records Scotland.

Wales: Secure Anonymised Information Linkage (SAIL system) [7]. This will utilise the Controlling Covid (ConCOV) platform linking records on 3.2 million people from the NHS population spine with hospital (Patient Episode Database for Wales), Welsh Longitudinal GP record (WLGP), Covid-19 test results from the Laboratory Information Management System (LIMS), and mortality and 2011Census data from the Office for National Statistics (ONS) [8]

Selection criteria:

Any individual in the relevant linked dataset between the ages of 19 and 100 will be included. Individuals who had an event (hospitalisation or death) in the first period (24 January 2020 – 30 April 2020) will be excluded from any analysis in the second period (1 May 2020 – 30 June 2020).

Exposure and Outcomes:

Table 1 and 2 list all exposure and outcomes variables respectively for the QCOVID algorithm, along with a description, variable type (e.g. integer, real, categorical) and possible values.

Table 1: Exposure variables in QCOVID algorithm.

Variable:	Description/Question:	Value:
**Demographic:**		
age	Age in years	Integer: 19-100
sex	Biological sex at birth	Categorical: female, male
town	Townsend Deprivation Score	Real number
ethnicity	Ethnicity	Categorical: White, Indian, Pakistani, Bangladeshi, Other Asian, Caribbean, Black African, Chinese, other ethnic group
homecat	What is your housing category - care home or homeless or neither?	Categorical: neither, care home, homeless

**Clinical:**		
bmi	Body Mass Index (kg/m^2)	Positive real number
chemocat	Have you had chemotherapy in the last 12 months?	Categorical: none, group A, group B, group C

For peer review only

learncat	Do you have a learning disability or Down's Syndrome?	Categorical: learning disability, Down syndrome
renalcat	Chronic Kidney Disease (CKD) stage	Categorical: No serious kidney disease, CKD stage 3, CKD stage 4, CKD stage 5 without dialysis or transplant, CKD stage 5 with dialysis in last 12 months, CKD stage 5 with transplant
diabetescat	Do you have diabetes?	Categorical: none, type 1, type 2
b2_82	Have you been prescribed immunosuppressants four or more times in the previous 6 months?	Categorical: yes, no
b2_leukolaba	Have you been prescribed anti-leukotriene or long acting beta2-agonists (LABA) four or more times in the previous 6 months?	Categorical: yes, no
b2_prednisone	Have you been prescribed oral predisolone containing preparations prescribed four or more times in the previous 6 months?	Categorical: yes, no
b_AF	Do you have atrial fibrillation?	Categorical: yes, no
b_CCF	Do you have heart failure?	Categorical: yes, no
b_asthma	Do you have asthma?	Categorical: yes, no
b_bloodcancer	Have you a cancer of the blood or bone marrow such as leukaemia, myelodysplastic syndromes,	Categorical: yes, no

For peer review only

	lymphoma or myeloma and are at any stage of treatment?	
b_cerebralpalsay	Do you have cerebral palsy?	Categorical: yes, no
b_chd	Do you have coronary heart disease?	Categorical: yes, no
b_cirrhosis	Do you have cirrhosis of the liver?	Categorical: yes, no
b_congenheart	Do you have congenital heart disease or have you had surgery for it in the past?	Categorical: yes, no
b_copd	Do you have chronic obstructive pulmonary disease (COPD)?	Categorical: yes, no
b_dementia	Do you have dementia?	Categorical: yes, no
b_epilepsy	Do you have epilepsy?	Categorical: yes, no
b_fracture4	Have you had a prior fracture of hip, wrist, spine or humerus?	Categorical: yes, no
b_neurorare	Do you have motor neurone disease, multiple sclerosis, myaesthenia, or Huntingtons's Chorea?	Categorical: yes, no
b_parkinsons	Do you have Parkinson's disease?	Categorical: yes, no
b_pulmhyper	Do you have pulmonary hypertension or pulmonary fibrosis?	Categorical: yes, no
b_pulmrare	Do you have cystic fibrosis or bronchiectasis or alveolitis?	Categorical: yes, no
b_pvd	Do you have peripheral vascular disease?	Categorical: yes, no
b_ra_sle	Do you have rheumatoid arthritis or SLE?	Categorical: yes, no
b_respcancer	Do you have lung or oral cancer?	Categorical: yes, no
b_semi	Do you have severe mental illness?	Categorical: yes, no

For peer review only

b_sicklecelldisease	Do you have sickle cell disease or severe combined immune deficiency syndromes?	Categorical: yes, no
b_stroke	Have you had a stroke or TIA?	Categorical: yes, no
b_vte	Have you had a thrombosis or pulmonary embolus?	Categorical: yes, no
p_marrow6	Have you had a bone marrow or stem cell transplant in the last 6 months?	Categorical: yes, no
p_radio6	Have you had radiotherapy in the last 6 months?	Categorical: yes, no
p_solidtransplant	Have you had a solid organ transplant (lung, liver, stomach, pancreas, spleen, heart or thymus)?	Categorical: yes, no

Table 2: Outcomes variables in QCOVID algorithm.

**Variable:**	**Description/Question:**	**Value:**
Time to Covid-19 hospitalisation	Time to hospitalisation with RT-PCR confirmed Covid-19 infection in the cohort period in days.	Real number: 0-91
Time to Covid-19 death	Time to death with Covid-19 confirmed or suspected on their death certificate, or confirmed by RT-PCR test, in the cohort period in days.	Real number: 0-91

Whenever available, all variables will be taken as the most recent recorded value in the relevant dataset at the date of entry into the cohort. The Townsend Deprivation Score (TDS) will be determined by matching available residential location information with output area and the

For peer review only

corresponding TDS from the 2011 UK census [9]. Categories for the variable chemocat will be determined using the lookup table in the supplemental materials.

Data cleaning:

The following procedures will be used for data cleaning:

- **diabetes_cat:** If the most recent entry has both type 1 and types 2 recorded, diabetes_cat will be set to type 2.
- **BMI:** The most recently recorded patient BMI within the last 5 years. If the most recently recorded BMI is from more than 5 years ago at the search date, bmi will be set to missing value.
- **learncat:** If a patient is recorded has having both learning disability and Down's syndrome, learncat will be set to Down's syndrome.

Missing data:

For comorbidities and medication use and treatments, missing values will be taken to mean absence of that factor. Missing values for ethnicity will be set to "White". For any other missing values, a single imputation will be considered. The following methods may be considered for use in the imputation: predictive mean matching, least squares, logistic and multinomial models, imputation by chained equations.

Statistical Analysis:

Each validation exercise will report a table of cohort characteristics, following Table 2 in [1]. The main performance metrics that will be calculated are R^2 [9], Harrell's C, Royston's D [10] and the Brier score. Different stratifications for these statistics will be considered, including by age, sex and time period. 95% confidence intervals will be reported for R^2, Harrell's C and Royston's D. Graphs of observed and predicted probability of hospital admission and death by vigintile for stratified subgroups will be reported, following [1]. Other analyses/reporting measures will also be considered.

Sample Size:

A preliminary sample size calculation can be done using figures from the original paper [1]. Using the estimated standard deviation of Harrell's C for females in the first time period and assuming

For peer review only - http://bmjopen.bmj.com/site/about/guidelines.xhtml

Harrell's C is asymptotically normally distributed implies that a sample size of approximately 5,714 would be sufficient to detect a true value for Harrell's C that is greater than or equal to 0.8 with 80% power. Repeating this calculation for other population subgroups and time periods yields results of a similar magnitude. The samples sizes in the planned studies will be on the order of hundreds of thousands or millions.

Ethics, reporting and dissemination

The ethics approval for the development and validation of QCOVID in England was granted by the East Midlands-Derby Research Ethics Committee [reference 18/EM/0400]. For Scotland, approvals have been obtained by the National Research Ethics Service Committee (REC), South East Scotland 02 (REC number: 12/SS/0201) and the Public Benefit and Privacy Panel for Health and Social Care (reference number: 1920-0279). The data to be used in this study for Wales are available in the SAIL Databank at Swansea University, Swansea, UK. All proposals to use SAIL data are subject to review by an independent Information Governance Review Panel (IGRP). Before any data can be accessed, approval must be given by the IGRP. The IGRP gives careful consideration to each project to ensure proper and appropriate use of SAIL data. When access has been approved, it is gained through a privacy-protecting safe haven and remote access system referred to as the SAIL Gateway. SAIL has established an application process to be followed by anyone who would like to access data via SAIL.[7] Findings will be presented at conferences, published in peer-reviewed journals and to the funders and government COVID-19 advisory bodies as appropriate. Strengthening the Reporting of Observational Studies in Epidemiology (STROBE) and Reporting of studies Conducted using Observational Routinely-collected Data (RECORD) (via the COVID-19 extension) checklists will guide our study findings reporting. The Northern Ireland validation study proposal is under review by the NITRE for HSC data accessed via Northern Ireland Honest Broker Service; an Ethics application has been submitted through IRAS.

Author's Contributions:

AS conceived this protocol. CR, VH, FK, TC, JHC, BH, CC, RAL and JL provided country specific information about available data and analysis plans. SK wrote drafts of this protocol. All authors gave final approval of the version to be published.

Acknowledgments

This work will use data provided by patients and collected by a number of organisations. We would like to acknowledge all patients who shared their information as well as all data providers who make anonymised data available for research. In particular, Public Health Scotland, Public Health

For peer review only

Wales, Public Health England, the NHS, the SAIL databank, and the Office for National Statistics.

Funding:

The validation in England will be funded by a grant from the National Institute for Health Research following a commission by the Chief Medical Officer for England. In Scotland, EAVE II is funded by the Medical Research Council [MR/R008345/1] and supported by the Scottish Government. In Wales, ConCOV is supported by the Medical Research Council [MR/V028367/1].

Competing interests:

AS reports grants from NIHR, grants from MRC, and grants from HRR UK, during the conduct of the study. JL and RAL report grants from UKRI Medical Research Council, during the conduct of the study. JHC reports grants from John Fell Oxford University Press Research Fund, grants from Cancer Research UK (CR-UK) grant number C5255/A18085, through the Cancer Research UK Oxford Centre, grants from the Oxford Wellcome Institutional Strategic Support Fund (204826/Z/16/Z), grants from NIHR, during the conduct of the study; personal fees and other from ClinRisk Ltd, outside the submitted work; and JHC is an unpaid director of QResearch, a not-for-profit organisation which is a partnership between the University of Oxford and EMIS Health who supply the QResearch database used for this work. Carol Coupland reports personal fees from ClinRisk ltd, outside the submitted work. JHC, AS, and Carol Coupland were members of the research team involved in the development of the QCOVID risk prediction algorithm. All other authors report no conflict of interest.

Patient and Public Involvement:

There are no plans for Patient and Public Involvement in this research.

References:

1. Clift AK, Coupland CAC, Keogh RH, Diaz-Ordaz K, Williamson E, Harrison EM, Hayward A, Hemingway H, Horby P, Mehta N, Benger J, Khunti K, Spiegelhalter D, Sheikh A, Valabhji J, Lyons RA, Robson J, Semple MG, Kee F, Johnson P, Jebb S, Williams T, Hippisley-Cox J. Living risk prediction algorithm (QCOVID) for risk of hospital admission and mortality from coronavirus 19 in adults: national derivation and validation cohort study. BMJ. 2020 Oct 20;371:m3731. doi: 10.1136/bmj.m3731. PMID: 33082154; PMCID: PMC7574532.

2. Townsend, P., Phillimore, P. and Beattie, A. (1988) Health and Deprivation: Inequality and the North. Routledge, London. doi: 10.7748/ns.2.17.34.s66. PMID: 27415096.

3. Qresearch, https://www.qresearch.org/

For peer review only

4. Hippisley-Cox J, Clift AK, Coupland CAC, et al. Protocol for the development and evaluation of a tool for predicting risk of short-term adverse outcomes due to COVID-19 in the general UK population. *MedRxiv* 2020:2020.06.28.20141986-2020.06.28

5. https://www.gov.uk/government/news/new-technology-to-help-identify-those-at-high-risk-from-covid-19

6. EAVE II, The Usher Institute, https://www.ed.ac.uk/usher/eave-ii

7. The Secure Anonymised Information Linkage (SAIL) databank. Available at: https://saildatabank.com

8. Lyons J, Akbari A, Torabi F, Davies G, North L, Griffiths R, Bailey R, Hollinghurst J, Fry R, Turner S, Thompson D, Rafferty J, Mizen A, Orton C, Ellwood-Thompson S, Au-Yeung L, Cross L, Gravenor M, Brophy S, Lucini B, John A, Szakmany T, Davies J, Davies C, Williams C, Emmerson C, Cottrell S, Connor T, Taylor C, Pugh R, Diggle PJ, John G, Scourfield S, Hunt J, Cunningham AM, Helliwell K, Lyons RA. (2020) Understanding and responding to COVID19 in Wales: protocol for a privacy protecting data platform for enhanced epidemiology and evaluation of interventions. BMJ Open 2020;10:e043010. doi:10.1136/ bmjopen-2020-043010

9. 2011 UK Census, Townsend Deprivation Scores, https://www.statistics.digitalresources.jisc.ac.uk/dataset/2011-uk-townsend-deprivation-scores

10. Royston P, Sauerbrei W. A new measure of prognostic separation in survival data. Stat Med. 2004 Mar 15;23(5):723-48. doi: 10.1002/sim.1621. PMID: 14981672.

For peer review only

Sheet3b - Chemotherapy lookup

final classification	map
Group A	1 & 2
Group B	3 & 4
Group C	5

Bench Mark Group	Group
TRASTUZUMAB	1
ZOLEDRONIC ACID	1
DENOSUMAB	1
PEMBROLIZUMAB	1
PERTUZUMAB + TRASTUZUMAB	1
ENZALUTAMIDE	1
ABIRATERONE	1
NIVOLUMAB	1
CETUXIMAB	1
ATEZOLIZUMAB	1
BCG	1
BEVACIZUMAB	1
AFATINIB	1
FULVESTRANT	1
IPILIMUMAB + NIVOLUMAB	1
GOSERELIN	1
METHOTREXATE INTRATHECAL	1
PANITUMUMAB	1
LANREOTIDE	1
DURVALUMAB	1
PERTUZUMAB	1
PAMIDRONATE	1
MITOMYCIN INTRAVESICULAR	1
OCTREOTIDE	1
IMMUNOGLOBULIN	1
ANAGRELIDE	1
LETROZOLE	1
AVELUMAB	1
TRIPLE INTRATHECAL	1
CETUXIMAB + RT	1
BICALUTAMIDE	1
TRIPTORELIN	1
IPILIMUMAB	1
PRISM TRIAL	1
CEMIPLIMAB	1
NERATINIB	1
ANASTROZOLE	1
VISMODEGIB	1
IBANDRONIC ACID	1
TAMOXIFEN	1
CYTARABINE INTRATHECAL	1
EXEMESTANE	1
CYPROTERONE	1
TALIMOGENE LAHERPAREPVEC	1
LEUPRORELIN	1
DEGARELIX	1
ELOQUENT2 TRIAL	1
SILTUXIMAB	1
ELOQUENT TRIAL	1
BICALUTAMIDE + LEUPRORELIN	1
CONFIRM TRIAL	1
FAKTION TRIAL	1
APALUTAMIDE	1
EPIRUBICIN INTRAVESICULAR	1
STILBOESTROL	1
DARBEPOETIN	1
FLUOROURACIL + PANITUMUMAB	1
AVAPRITINIB	1
FURVA TRIAL	1
PERM TRIAL	1
ENZARAD TRIAL	1
BICALUTAMIDE + GOSERELIN	1
NB 13 CIS-RA (ISOTRETINOIN)	1
VORINOSTAT	1
ABIRATERONE + ENZALUTAMIDE	1
PARAGON TRIAL	1
MEDROXYPROGESTERONE	1
KESTREL TRIAL	1
MEGESTROL	1
XILONIX	1
RAMUCIRUMAB	1
CAM-PLEX TRIAL	1
BICALUTAMIDE + TAMOXIFEN	1
LATITUDE TRIAL	1
IPILIMUMAB + PEMBROLIZUMAB	1
QUADRUPLE INTRATHECAL	1
GOSERELIN + LEUPRORELIN	1
MERCAPTOPURINE + VINCRISTINE	1
PROGESTERONE	1
CLODRONIC ACID	1
CYPROTERONE + GOSERELIN	1
GOSERELIN + LETROZOLE	1
CISPLATIN + GEMCITABINE	2
HYDROXYCARBAMIDE	2
CAPECITABINE	2
CISPLATIN + RT	2
PALBOCICLIB	2
FLUOROURACIL	2
CISPLATIN	2
CAPECITABINE + IRINOTECAN	2
CISPLATIN + PEMETREXED	2
DABRAFENIB + TRAMETINIB	2
CAPECITABINE + GEMCITABINE	2
LETROZOLE + PALBOCICLIB	2
PEMETREXED	2
VINORELBINE	2
CISPLATIN + FLUOROURACIL	2
EVEROLIMUS	2
PAZOPANIB	2
SUNITINIB	2
ABEMACICLIB + FULVESTRANT	2
CABOZANTINIB	2
HCX	2
NIRAPARIB	2
CAPECITABINE + RT	2
PEMBROLIZUMAB + PEMETREXED	2
METHOTREXATE	2
GEFITINIB	2
OSIMERTINIB	2
ERLOTINIB	2
RIBOCICLIB	2
FLUOROURACIL + MITOMYCIN + RT	2
OLAPARIB	2
SORAFENIB	2
VINBLASTINE	2
AXITINIB	2
ALECTINIB	2
CAPECITABINE + CISPLATIN + RT	2
CISPLATIN + FLUOROURACIL + RT	2
TIVOZANIB	2
CRIZOTINIB	2
CAPECITABINE + MITOMYCIN	2
CAPECITABINE + VINORELBINE	2
DABRAFENIB	2
ABEMACICLIB	2
LENVATINIB	2
LETROZOLE + RIBOCICLIB	2
CAPECITABINE + MITOMYCIN + RT	2
BEXAROTENE	2
MITOMYCIN	2
INTERFERON	2
PONATINIB	2
VINCRISTINE	2
BUSULPHAN	2
CERITINIB	2
CAPECITABINE + TRASTUZUMAB	2
FLUOROURACIL + MITOMYCIN	2
NINTEDANIB	2
FLUOROURACIL + RT	2
REGORAFENIB	2
ANASTROZOLE + PALBOCICLIB	2
CETUXIMAB + CISPLATIN + FU	2
EVEROLIMUS + LENVATINIB	2
BRIGATINIB	2
LORLATINIB	2
CETUXIMAB + MDG	2
DICE TRIAL	2
BEVACIZUMAB + CAPECITABINE	2
TRASTUZUMAB + VINORELBINE	2
Fulvestrant + Palbociclib	2
Abemaciclib + Letrozole	2
ACELARATE TRIAL	2
VANDETANIB	2
EXEMESTANE + PALBOCICLIB	2
CISPLATIN + TRASTUZUMAB	2
INTERIM TRIAL	2
TRAMETINIB	2
UNIRAD TRIAL	2
MITOXANTRONE	2
CAPECITABINE + LAPATINIB	2
ICON9 TRIAL	2
ATEZOLIZUMAB + BEVACIZUMAB	2
COSMIC TRIAL	2
STAR TRIAL	2
RE-AKT TRIAL	2
BEACON TRIAL	2
DACOMITINIB	2
COLUMBUS TRIAL	2
PACKER MAINTENANCE	2
CAPECITABINE + NERATINIB	2
ANAGRELIDE + HYDROXYCARBAMIDE	2
INTERFERON BETA	2
CISPLATIN + FLUOROURACIL + TRASTUZUMAB	2
QUIZARTINIB	2
ASCIMINIB	2
PAKT TRIAL	2
METHOTREXATE + PEGASPARGASE	2
POLEM TRIAL	2
CISPLATIN + FLUORO + STREPTOZOCIN	2
CAP + RT	2
COMBI-AD TRIAL	2
OLYMPIA TRIAL	2
SPIRIT TRIAL	2
CEDIRANIB	2
FLUOROURACIL + INTERFERON ALFA	2
MILLENNIUM C16021 TRIAL	2
ENCORAFENIB	2
TALAZOPARIB	2
EVEROLIMUS + EXEMESTANE	2
CALYPSO TRIAL	2
INTERFERON ALFA + ZIDOVUDINE	2
IVOSIDENIB	2
SOLO1 TRIAL	2
LU-177	2
MITOMYCIN + RALTITREXED	2
NIMRAD TRIAL	2
SIROLIMUS	2
METHOTREXATE + TRASTUZUMAB	2
PALLET TRIAL	2
TOPARP TRIAL	2
TIPIFARNIB	2
RITUXIMAB + VINBLASTINE	2
IMATINIB + HYDROXYCARBAMIDE	2
METHOTREXATE + VINCRISTINE	2
BELLE3 TRIAL	2
GOSERELIN + PALBOCICLIB	2
PAZ02 TRIAL	2
PIN TRIAL	2
LAPATINIB	2
CASPS TRIAL	2
CISPLATIN + IMATINIB	2
TEMSIROLIMUS	2
LURBINECTIDIN	2
ERDAFITINIB	2
TIMELY TRIAL	2
COBIMETINIB	2
MASITINIB	2
BINIMETINIB	2
CREATE TRIAL	2
IRINOTECAN + MDG	3
OXALIPLATIN + MDG	3
CAPECITABINE + OXALIPLATIN	3
CETUXIMAB + IRINOTECAN + MDG	3
FLUOROURACIL + IRINOTECAN + OXALIPLATIN	3
CVD	3
RITUXIMAB	3
BORTEZOMIB + THALIDOMIDE	3
DARATUMUMAB	3
LENALIDOMIDE	3
IXAZOMIB + LENALIDOMIDE	3
BORTEZOMIB + DARATUMUMAB	3
IMATINIB	3
IBRUTINIB	3
CVP R	3
BORTEZOMIB	3
CISPLATIN + VINORELBINE	3
CABAZITAXEL	3
GEMCITABINE	3
CISPLATIN + ETOPOSIDE	3
MPV	3
TEMOZOLOMIDE	3
TRASTUZUMAB EMTANSINE	3
CETUXIMAB + OXALIPLATIN + MDG	3
CTD	3
OXALIPLATIN + MDG + PANITUMUMAB	3
ECX	3
RUXOLITINIB	3
CARFILZOMIB	3
IRINOTECAN + MDG + PANITUMUMAB	3
POMALIDOMIDE	3
IRINOTECAN + PANITUMUMAB + MDG	3
NILOTINIB	3
CAPECITABINE + CISPLATIN	3
CARDAMON TRIAL	3
BORTEZOMIB + PANOBINOSTAT	3
CYCLOPHOSPHAMIDE + LENALIDOMIDE	3
CVP + OBINUTUZUMAB	3
DASATINIB	3
TEMOZOLOMIDE + RT	3
MUK 9 TRIAL	3
IRINOTECAN	3
CYCLOPHOSPHAMIDE + RITUXIMAB + VINCRISTINE	3
VENETOCLAX	3
CYCLOPHOSPHAMIDE + RITUXIMAB	3
CARBO + FLUOROURACIL	3
CISPLATIN + GEMCITABINE + RITUXIMAB	3
OXALIPLATIN + RALTITREXED	3
CISPLATIN + PEMBROLIZUMAB + PEMETREXED	3
PEG INTERFERON	3
BOSUTINIB	3
OBINUTUZUMAB	3
CAPECITABINE + CETUXIMAB + IRINOTECAN	3
MPT	3
CISPLATIN + ETOPOSIDE + RT	3
IXAZOMIB	3
THALIDOMIDE	3
ETOPOSIDE	3
CAPECITABINE + TEMOZOLOMIDE	3
IRINOTECAN + TEMOZOLOMIDE	3
MERCAPTOPURINE + METHOTREXATE	3
BINIMETINIB + ENCORAFENIB	3
GEMCITABINE + OXALIPLATIN	3
BLINATUMOMAB	3
CMF	3
BEVACIZUMAB + OXALIPLATIN + MdG	3
BEVACIZUMAB + IRINOTECAN + MdG	3
CONCEPT TRIAL	3
OLARATUMAB	3
RALTITREXED	3
CETUXIMAB + CISPLATIN + FLUOROURACIL	3
GEMCITABINE + RT	3
IDELALISIB	3
BEVACIZUMAB + IRINOTECAN	3
BEVACIZUMAB + IRINOTECAN + MDG	3
IRINOTECAN + TEMOZOLOMIDE + VINCRISTINE	3
BOSTON TRIAL	3
CAPE + OXALIPLATIN + TRASTUZUMAB	3
VEMURAFENIB	3
IPM	3
CYCLOPHOSPHAMIDE + ETOPOSIDE	3
PENTOSTATIN	3
IPO	3
CYCLOPHOSPHAMIDE + MTX	3
PANOBINOSTAT	3
OXALIPLATIN	3
IRINOTECAN + BEVACIZUMAB	3
MITOTANE	3
FOCUS4 TRIAL	3
CYCLOPHOSPHAMIDE + VINCRISTINE	3
CAPECITABINE + CETUXIMAB + OXALIPATIN	3
CETUXIMAB + IRINOTECAN	3
COMPLEMENT TRIAL	3
AFLIBERCEPT + FU + IRINOTECAN	3
BEVACIZUMAB + CAPE + OXALIPLATIN	3
CVD + THALIDOMIDE	3
BEVACIZUMAB + PACLITAXEL	3
IRINOTECAN + RALTITREXED	3
IRINOTECAN + VINCRISTINE	3
CARFILZOMIB + CYCLOPHOSPHAMIDE	3
CLARITY TRIAL	3
BEVACIZUMAB + IRINOTECAN + OXALIPLATIN + MDG	3
MMM	3
LENALIDOMIDE + RITUXIMAB	3
CAPECITABINE + OXALIPLATIN + RT	3
PACLITAXEL + BEVACIZUMAB	3
BEVACIZUMAB + CAPE + IRINOTECAN	3
SHAMASH LBCMVD-56	3
PRIMUS 002 TRIAL	3
CYCLO + RITUXIMAB + VINCRISTINE	3
FLUOROURACIL + IRINOTECAN	3
MERCAPTOPURINE	3
PLATO TRIAL	3
CAPITAL TRIAL	3
BALLAD TRIAL	3
PACLITAXEL + RAMUCIRUMAB	3
DARATUMUMAB + LENALIDOMIDE	3
NEPTUNE TRIAL	3
FLUOROURACIL + OXALIPLATIN + TRASTUZUMAB	3
CAPECITABINE + CISPLATIN + TRASTUZUMAB	3
CYCLOPHOSPHAMIDE + FLUOROURACIL + METHOTREXATE	3
METHOTREXATE + RITUXIMAB	3
PREDNISOLONE + VINBLASTINE + RITUXIMAB	3
COMPLEEMENT-1 TRIAL	3
BEVACIZUMAB + OXALIPLATIN	3
CAPECITABINE + IRINOTECAN + PANITUMUMAB	3
IBRUTINIB + VENETOCLAX	3
CCISV	3
SOPHIA TRIAL	3
BORTEZOMIB + VENETOCLAX	3
TREOSULFAN	3
DEPATUXIZUMAB MAFODOTIN + TEMOZOLOMIDE	3
DARATUMUMAB + POMALIDOMIDE	3
RCD TRIAL	3
OFATUMUMAB	3
CAPECITABINE + CETUXIMAB + OXALIPLATIN	3
CAPECITABINE + OXALIPLATIN + PANITUMUMAB	3
CISPLATIN + TEYSUNO	3
GEMCITABINE + OXALIPLATIN + PACLITAXEL	3
BEVACIZUMAB + TEMOZOLOMIDE	3
BORTEZOMIB + LENALIDOMIDE	3
TEYSUNO	3
ELOTUZUMAB + LENALIDOMIDE	3
BORTEZOMIB + PREDNISOLONE + MELPHALAN	3
ALEMTUZUMAB + CYCLOPHOSPHAMIDE + METHOTREXATE	3
MERCAPTOPURINE + METHOTREXATE + TRETINOIN	3
METHOTREXATE + PROCARBAZINE	3
CNS EPEN2007	3
CNS SIOP 2009	3
IRINOTECAN + MITOMYCIN	3
CYCLOPHOSPHAMIDE + GEMCITABINE + VINCRISTINE	3
LENALIDOMIDE + DARATUMUMAB	3
CAPE + CETUXIMAB + OXALIPLATIN	3
Cisplatin + Irinotecan	3
IXAZOMIB + THALIDOMIDE	3
CARFILZOMIB + POMALIDOMIDE	3
CYCLOPHOSHAMIDE + RITUXIMAB	3
ALEMTUZUMAB + ETOPOSIDE	3
SELINEXOR	3
STREPTOZOCIN	3
MCX	3
BENDAMUSTINE + BORTEZOMIB	3
GEMCITABINE + TEGAFUR	3
PATHOS TRIAL	3
CAPECITABINE + OXALIPLATIN + TRASTUZUMAB	3
CETUXIMAB + IRINOTECAN + RALTITREXED	3
IRINOTECAN + OXALIPLATIN + PACLITAXEL	3
PROMISE-MESO TRIAL	3
SCOPE 1 TRIAL	3
CYCLOPHOSPHAMIDE + RITUXIMAB + VINBLASTINE	3
MERCAPTOPURINE + VINBLASTINE	3
BEVACIZUMAB + IRINOTECAN + TEMOZOLOMIDE	3
CETUXIMAB + OXALIPLATIN	3
ACALABRUTINIB	3
BORTEZOMIB + RITUXIMAB	3
CYCLET	3
IBRUTINIB + RITUXIMAB	3
ABC-08 TRIAL	3
CNS HR MBL Cyclo/Vinc	3
FLUOROURACIL + GEMCITABINE	3
MANTA TRIAL	3
CETUXIMAB + OXALIPLATIN + RALTITREXED	3
VIM TRIAL	3
BEVACIZUMAB + OXALIPLATIN + MDG	3
CYCLOPHOSPHAMIDE + THALIDOMIDE + Dexamethasone	3
IRINOTECAN + OXALIPLATIN + PACLITAXEL	3
IRINOTECAN + PANITUMUMAB + RALTITREXED	3
IRINOTECAN + TALAZOPARIB	3
GELOX	3
PACRITINIB	3
PHITT D3 Vincristine / irinotecan	3
MELPHALAN + THALIDOMIDE	3
MM	3
SHINE TRIAL	3
VE	3
AFLIBERCEPT + CAPECITABINE + IRINOTECAN	3
CLL210 TRIAL	3
CISPLATIN + CYCLOPHOSPHAMIDE + ETOPOSIDE	3
CISPLATIN + RALTITREXED + TRASTUZUMAB	3
AFLIBERCEPT + FLUOROURACIL + IRINOTECAN	3
CISPLATIN + CYCLOPHOSPHAMIDE + VINCRISTINE	3
CYCLOPHOSPHAMIDE + TEMOZOLOMIDE	3
IRINOTECAN + TEMOZOLOMIDE + DINUTUXIMAB BETA	3
CARFILZOMIB + CYCLOPHOSPHAMIDE + LENALIDOMIDE	3
PACIFICO TRIAL	3
CYCLOPHOSPHAMIDE + IXAZOMIB	3
LIPOSOMAL DOXORUBICIN + PACLITAXEL	3
Ofatumumab	3
RITUXIMAB + TEMOZOLOMIDE	3
PACLITAXEL	4
CARBOPLATIN + PACLITAXEL	4
CHOP R	4
DOCETAXEL	4
GEMCARBO	4
CARBOPLATIN + ETOPOSIDE	4
EC	4
UKALL2011	4
DOCETAXEL + PERTUZUMAB + TRASTUZUMAB	4
ABVD	4
FEC + DOCETAXEL	4
BENDAMUSTINE + RITUXIMAB	4
CARBOPLATIN	4
CARBOPLATIN + PEMETREXED	4
DOCETAXEL + FLUOROURACIL + OXALIPLATIN	4
ERIBULIN	4
FEC 100	4
CARBOPLATIN + LIPOSOMAL DOX	4
CARBOPLATIN + DOCETAXEL + PERTUZUMAB + TRASTUZUMAB	4
CARBOPLATIN + VINORELBINE	4
EOX	4
CARBOPLATIN + PEMBROLIZUMAB + PEMETREXED	4
FEC	4
FEC 60 OR 75	4
GEMCITABINE + NAB-PACLITAXEL	4
FEC 100 + DOCETAXEL	4
CYCLOPHOSPHAMIDE + DOCETAXEL	4
CHLORAMBUCIL + OBINUTUZUMAB	4
BEVACIZUMAB + CARBOPLATIN + PACLITAXEL	4
CHOP	4
PACLITAXEL + TRASTUZUMAB	4
CAPECITABINE + EPIRUBICIN + OXALIPLATIN	4
PARTNER TRIAL	4
EC + PACLITAXEL	4
CARBOPLATIN + PACLITAXEL + RT	4
PCV	4
CISPLATIN + VINORELBINE + RT	4
BEVACIZUMAB + CARBO + PACLITAXEL	4
FEC + DOCETAXEL + PERTUZUMAB + TRASTUZUMAB	4
R-EPOCH	4
EPIRUBICIN	4
BENDAMUSTINE + OBINUTUZUMAB	4
STAMPEDE TRIAL	4
CYCLOPHOSPHAMIDE + DOCETAXEL + EPIRUBICIN	4
LIPOSOMAL DOXORUBICIN	4
CAV	4
EC + DOCETAXEL	4
CARBOPLATIN + DOCETAXEL	4
CYCLO + DOXORUBICIN + PACLITAXEL	4
ROSCO TRIAL	4
BEACOPP	4
CAPECITABINE + CARBOPLATIN	4
DOCETAXEL + NINTEDANIB	4
CARBOPLATIN + DOCETAXEL + TRASTUZUMAB	4
CISPLATIN + DOCETAXEL + FLUOROURACIL	4
MELPHALAN	4
CHLORAMBUCIL + RITUXIMAB	4
NAB-PACLITAXEL	4
MYELOMA XI TRIAL	4
DOXORUBICIN + IFOSFAMIDE	4
DTPACE	4
DOXORUBICIN + OLARATUMAB	4
ICON8B TRIAL	4
ICE-R	4
FEC + DOCETAXEL + TRASTUZUMAB	4
ESHAP	4
BRENTUXIMAB	4
ESCALATED BEACOP DAC	4
CHOP + OBINUTUZUMAB	4
TIP	4
DOCETAXEL + GEMCITABINE	4
TRABECTEDIN	4
CYCLOPHOSPHAMIDE + DOCETAXEL + DOXORUBICIN	4
EC + DOCETAXEL + PERTUZUMAB	4
RCEOP	4
ECF	4
DOXORUBICIN	4
VDT-PACE	4
TOPOTECAN	4
CHLVPP	4
BENDAMUSTINE	4
AML MYECHILD	4
CYCLOPHOSPHAMIDE + GEMCITABINE + RITUXIMAB + VINCRISTINE	4
CYCLOPHOSPHAMIDE + DOCETAXEL + TRASTUZUMAB	4
GDP	4
OEPA	4
CARBOPLATIN + CYCLOPHOSPHAMIDE + DOXORUBICIN + PACLITAX	4
ETOPOSIDE + IFOSFAMIDE	4
FEC + TRASTUZUMAB	4
PACLITAXEL + PERTUZUMAB + TRASTUZUMAB	4
GEMCITABINE + OXALIPLATIN + RITUXIMAB	4
DOCETAXEL + TRASTUZUMAB	4
EURO-EWING	4
CHLORAMBUCIL	4
ENRICH TRIAL	4
ATEZOLIZUMAB + BEVACIZUMAB + CARBOPLATIN + PACLITAXEL	4
AC	4
CHOEP	4
CARBOPLATIN + RT	4
CYCLOPHOSPHAMIDE + DOCETAXEL + DOXORUBICIN + PERTUZUM	4
CYCLOPHOSPHAMIDE + TOPOTECAN	4
VIDE	4
LOMUSTINE	4
ICE	4
CYCLOPHOSPHAMIDE + DOXORUBICIN + PERTUZUMAB + TRASTUZU	4
ESHAP R	4
CYCLOPHOSHAMIDE	4
IVE	4
GEMCITABINE + PACLITAXEL	4
RITUXIMAB + VENETOCLAX	4
ECARBOX	4
RMS 2005 TRIAL	4
CARBOPLATIN + VINORELBINE + RT	4
CYCLOPHOSPHAMIDE + ETOPOSIDE + PROCARBAZINE	4
CLADRIBINE	4
BENDAMUSTINE + CYTARABINE + RITUXIMAB	4
PVD	4
IDELALISIB + RITUXIMAB	4
IFOSFAMIDE	4
SCALOP-2 TRIAL	4
FEC + PACLITAXEL	4
CARBOPLATIN + CYCLOPHOSPHAMIDE + EPIRUBICIN + PACLITAXEL	4
CAPECITABINE + CISPLATIN + EPIRUBICIN	4
VIP	4
MIFAMURTIDE	4
ATEZOLIZUMAB + NAB PACLITAXEL	4
DHAP - R	4
IVA	4
ATEZOLIZUMAB + CARBOPLATIN + ETOPOSIDE	4
CISPLATIN + DOXORUBICIN	4
FEC 100 + TRASTUZUMAB	4
MAP	4
VEPEMB	4
IGEV	4
CARBOPLATIN + CYCLOPHOSPHAMIDE + EPIRUBICIN + PACLITAXEL	4
EP	4
BENDAMUSTINE + THALIDOMIDE	4
BORTEZOMIB + PANOBINOSTAT + THALIDOMIDE	4
FEC 100 + DOCETAXEL + TRASTUZUMAB	4
CARBOPLATIN + VINCRISTINE	4
FCARBOST	4
CYTARABINE + METHOTREXATE + RITUXIMAB + THIOTEPA	4
CAP	4
DACARBAZINE + DOXORUBICIN + VINBLASTINE	4
CARBOPLATIN + CETUXIMAB + FLUOROURACIL	4
CYCLO + DOXORUBICIN + VINCRISTINE	4
LIPOSOMAL DAUNORUBICIN + CYTARABINE (Vyxeos)	4
EMA/CO	4
CYCLO + DOCETAXEL + TRASTUZUMAB	4
PMITCEBO-R	4
CARBOPLATIN + EPIRUBICIN + PACLITAXEL	4
GDP R	4
CISPLATIN + PACLITAXEL	4
CYCLOPHOSPHAMIDE + DOCETAXEL + PERTUZUMAB + TRASTUZUM	4
CVD R	4
EOF	4
NHL 2003	4
CAPECITABINE + STREPTOZOCIN	4
CYCLOPHOSPHAMIDE + ETOPOSIDE + RITUXIMAB + VINCRISTINE	4
ACTICCA-1 TRIAL	4
DACTINOMYCIN + ETOPOSIDE	4
CVP	4
INTERLACE TRIAL	4
MVAC	4
ICON TRIAL	4
IVE-R	4
PLATFORM TRIAL	4
MUK8 TRIAL	4
CARBO + DOCETAXEL + TRASTUZUMAB	4
DACARBAZINE	4
LOMUSTINE + PROCARBAZINE + VINCRISTINE	4
EDP	4
VAI	4
CAPECITABINE + CARBOPLATIN + EPIRUBICIN	4
DOXORUBICIN EMBOLISATION	4
LUTETIUM-177	4
DHAP	4
BEACOP DAC	4
CARBOPLATIN + CETUXIMAB + FU	4
CHLORAMBUCIL + ETOPOSIDE + LOMUSTINE	4
R-CHOEP	4
METHOTREXATE + VINBLASTINE	4
CNS EPENDYMOMA II	4
COMPARE TRIAL	4
CYCLOPHOSPHAMIDE + DOXORUBICIN	4
POMB	4
CARBOPLATIN + NAB-PACLITAXEL	4
CYCLOPHOSPHAMIDE + DOCETAXEL + DOXORUBICIN + TRASTUZUM	4
CISPLATIN + FU + TRASTUZUMAB	4
ESPAC TRIAL	4
EDP + MITOTANE	4
PROCAID TRIAL	4
TAC	4
CYCLOPHOSPHAMIDE + ETOPOSIDE + LOMUSTINE	4
SCOPE2 TRIAL	4
IVAC	4
ABVD R	4
CISPLATIN + DOX + ETOPOSIDE	4
ACCEPT TRIAL	4
VEDEX	4
IVADO	4
CISPLATIN + ETOPOSIDE + IFOSFAMIDE	4
CEV	4
CISPLATIN + IFOSFAMIDE + PACLITAXEL	4
INTERAACT TRIAL	4
CVP R + GEMCITABINE	4
CYCLOPHOSPHAMIDE + CYTARABINE + MERCAPTOPURINE	4
CYCLOPHOSPHAMIDE + EPIRUBICIN + FLUOROURACIL + PERTUZUM	4
GEMCITABINE + TIP	4
CISPLATIN + LOMUSTINE + VINCRISTINE	4
CAPECITABINE + CARBOPLATIN + TRASTUZUMAB	4
BORTEZOMIB + MELPHALAN	4
LOMUSTINE + PROCARBAZINE	4
GEMCITABINE + IFOSFAMIDE + VINORELBINE	4
CARMUSTINE + THIOTEPA	4
R-GEM-P	4
DACTINOMYCIN + VINCRISTINE	4
SPIRE TRIAL	4
DACARBAZINE + GEMCITABINE	4
CYCLOPHOSPHAMIDE + ETOPOSIDE + RITUXIMAB	4
HD EuroNet-PHL-C2 DECOPDAC	4
ALEMTUZUMAB + CYCLOPHOSPHAMIDE	4
DINUTUXIMAB BETA	4
PHITT TRIAL	4
CISPLATIN + ETOPOSIDE + PACLITAXEL	4
CISPLATIN + TOPOTECAN	4
PIXANTRONE	4
CYCLOPHOSPHAMIDE + VINORELBINE	4
DACTINOMYCIN	4
ETOPOSIDE + IFOSFAMIDE + RITUXIMAB	4
IGEV R	4
EURAMOS TRIAL	4
PAD	4
FOXFIRE TRIAL	4
CAPECITABINE + CARBOPLATIN + RT	4
CHLORAMBUCIL + OFATUMUMAB	4
GEMCITABINE + TREOSULPHAN	4
ROMICAR TRIAL	4
NEO-AEGIS TRIAL	4
STREPTOZOCIN + MDG	4
CARBOPLATIN + TOPOTECAN	4
CAPECITABINE + CISPLATIN + STREPTOZOCIN	4
COPP + DACARBAZINE	4
FEC 60 OR 75 + DOCETAXEL	4
CISPLATIN + EPIRUBICIN + FLUOROURACIL	4
CMV	4
CYTARABINE + OXALIPLATIN + RITUXIMAB	4
CYCLO + ETOPOSIDE + VINCRISTINE	4
HD EURONET-PHL	4
FCIST	4
MUK7 TRIAL	4
BENDAMUSTINE + GEMCITABINE + VINORELBINE	4
RATHL TRIAL	4
METHOTREXATE + VINORELBINE	4
R-CHOP / R-DHAP	4
HEPATOBLASTOMA	4
CARBO + LIPOSOMAL DOX + PACLITAXEL	4
CLADRIBINE + RITUXIMAB	4
BLEOMYCIN	4
VDC	4
CHLORAMBUCIL + DEXAMETHASONE + ETOPOSIDE	4
CISPLATIN + EPIRUBICIN	4
EC + PACLITAXEL + PERTUZUMAB	4
EP/EMA	4
CARBOPLATIN + DOCETAXEL + FLUOROURACIL	4
GEMCITABINE + VINORELBINE	4
MUK 12 TRIAL	4
BENDAMUSTINE + BRENTUXIMAB	4
DACARBAZINE + DOXORUBICIN + VINCRISTINE	4
CYCLOPHOSPHAMIDE + DOXORUBICIN + RITUXIMAB	4
CYCLOPHOSPHAMIDE + DOXORUBICIN + ETOPOSIDE + VINCRISTINE	4
ECARBOF	4
CHLORAMBUCIL + LOMUSTINE	4
EPIRUBICIN + FLUOROURACIL + OXALIPLATIN	4
CYCLO + ETOPOSIDE + NELARABINE	4
PEI	4
WILMS REL ICE	4
CISPLATIN + IFOSFAMIDE + MESNA + VINBLASTINE	4
CARBOPLATIN + EPIRUBICIN + FLUOROURACIL	4
CYCLOPHOSPHAMIDE + ETOPOSIDE + PROCARBAZINE + RITUXIMAB	4
BORTEZOMIB + CISPLATIN + CYCLOPHOSPHAMIDE + DOXORUBICIN +	4
PENTOSTATIN + RITUXIMAB	4
CISPLATIN + FLUOROURACIL + STREPTOZOCIN	4
BEVACIZUMAB + CISPLATIN + PACLITAXEL	4
CHLVPP/EVA	4
CARBOPLATIN + DOXORUBICIN	4
PMITCEBO	4
HD EuroNet-PHL-C2 COPDAC	4
CHOP + INTERFERON ALFA + ZIDOVUDINE	4
CISPLATIN + DOCETAXEL	4
GEMCITABINE + RITUXIMAB	4
CVD (NEUROENDOCRINE)	4
CYTARABINE + ETOPOSIDE + TRETINOIN	4
DACARBAZINE + DOXORUBICIN	4
BLEOMYCIN + CISPLATIN + VINCRISTINE	4
FEC + PACLITAXEL + TRASTUZUMAB	4
PROCARBAZINE	4
FLUDARABINE	4
BORTEZOMIB + DOXORUBICIN	4
BLEOMYCIN + CISPLATIN + VINBLASTINE	4
CYCLOPHOSPHAMIDE + DACARBAZINE + VINCRISTINE	4
ETOPOSIDE + RITUXIMAB	4
HD EuroNet-PHL-C2 OEPA	4
CARFILZOMIB + LENALIDOMIDE	4
CISPLATIN + LIPOSOMAL DOXORUBICIN	4
EPSSG	4
CARBOPLATIN + PACLITAXEL + TRASTUZUMAB	4
SELPAC TRIAL	4
CHLORAMBUCIL + VINBLASTINE	4
CARBO + ETOPOSIDE + VINCRISTINE	4
NEOBLADE TRIAL	4
BOP	4
CISPLATIN + DACARBAZINE + VINBLASTINE	4
CYCLOPHOSPHAMIDE + DACTINOMYCIN + VINCRISTINE	4
ETOPOSIDE + LOMUSTINE + VINCRISTINE	4
CYCLO + FLUDARABINE + RITUXIMAB	4
INCA TRIAL	4
METAPHER TRIAL	4
CARBOPLATIN + EPIRUBICIN	4
CARBO + GEMCITABINE + RITUXIMAB	4
CAPE + CARBOPLATIN + CETUXIMAB	4
CARBO + THIOTEPA + TOPOTECAN	4
CARBOPLATIN + FLUOROURACIL + STREPTOZOCIN	4
BEVACIZUMAB + CARBO + GEMCITABINE	4
CARBOPLATIN + ETOPOSIDE + IFOSFAMIDE	4
CISPLATIN + DOXORUBICIN + ETOPOSIDE	4
CARBOPLATIN + CYCLOPHOSPHAMIDE + ETOPOSIDE	4
DACTINOMYCIN + ETOPOSIDE + METHOTREXATE	4
DACTINOMYCIN + CYCLO + VINCRISTINE	4
MUK6 TRIAL	4
POUT TRIAL	4
Bortezomib + DA-EPOCH-R	4
CISPLATIN + CYCLO + DOXORUBICIN	4
DA-EPOCH + BORTEZOMIB	4
RPACE-BOM	4
CYTARABINE + ETOPOSIDE	4
CAPECITABINE + DOCETAXEL	4
EPIRUBICIN + OXALIPLATIN	4
CHOP R + BORTEZOMIB	4
CYCLOPHOSPHAMIDE + CYTARABINE + MERCAPTOPURINE + METHO	4
CARBOPLATIN + NAB-PACLITAXEL + TRASTUZUMAB	4
EC + DOCETAXEL + TRASTUZUMAB	4
IFOSFAMIDE + PACLITAXEL	4
NAB-PACLITAXEL + TRASTUZUMAB	4
AC + DOCETAXEL + TRASTUZUMAB	4
GEMCITABINE + RITUXIMAB + VINORELBINE	4
CARBOPLATIN + EPIRUBICIN + ETOPOSIDE + MELPHALAN	4
DACTINOMYCIN + DOXORUBICIN + VINCRISTINE	4
LOMUSTINE + TEMOZOLOMIDE	4
CARBOPLATIN + ETOPOSIDE + VINCRISTINE	4
CYCLOPHOSPHAMIDE + PACLITAXEL	4
mEOC TRIAL	4
CHOP-R + CYTARABINE	4
CISPLATIN + DOXORUBICIN + METHOTREXATE	4
CISPLATIN + DOX + ETOPOSIDE + MITOTANE	4
OPTIMAL TRIAL	4
CARBOPLATIN + IFOSFAMIDE	4
CYCLOPHOSPHAMIDE + DOXORUBICIN + ETOPOSIDE	4
CYCLOPHOSPHAMIDE + FLUOROURACIL + METHOTREXATE + Trastuz	4
PETREA TRIAL	4
G-CVP	4
CISPLATIN + DACTINOMYCIN + ETOPOSIDE + METHOTREXATE	4
CARBOPLATIN + TRASTUZUMAB	4
CVAD	4
CYCLOPHOSPHAMIDE + DOXORUBICIN + METHOTREXATE + VINCRIS	4
CYCLOPHOSPHAMIDE + FLUDARABINE + THIOTEPA	4
LOPP	4
BUSULPHAN + CYCLO	4
CHLORAMBUCIL + DOXORUBICIN + ETOPOSIDE + PROCARBAZINE +	4
ETOPOSIDE + IFOSFAMIDE + RITUXIMAB + THIOTEPA	4
TIDE	4
MIC	4
PANTHER TRIAL	4
MELPHALAN + TOPOTECAN	4
CARBOPLATIN + ETOPOSIDE + PACLITAXEL	4
CYCLOPHOSPHAMIDE + DOXORUBICIN + PROCARBAZINE + VINCRIST	4
MITOXANTRONE + TRETINOIN	4
RETINOBLASTOMA	4
WT Relapse Carbo/Cyclo/Etop	4
NEMO TRIAL	4
BEVACIZUMAB + LOMUSTINE	4
CISPLATIN + NAB-PACLITAXEL	4
CARBOPLATIN + RALTITREXED	4
QUASAR 2 TRIAL	4
CISPLATIN + GEMCITABINE + PACLITAXEL	4
CYCLOPHOSPHAMIDE + METHOTREXATE	4
Z-DEX	4
PACKER INDUCTION VINCRISTINE + RT	4
BEVACIZUMAB + CARBOPLATIN + NAB-PACLITAXEL	4
DOXORUBICIN + GEMCITABINE	4
OCTOPUS TRIAL	4
ARISTOTLE TRIAL	4
CARBOPLATIN + EPIRUBICIN + METHOTREXATE + VINBLASTINE	4
CARBOPLATIN + FLUOROURACIL + TRASTUZUMAB	4
CISPLATIN + CYCLOPHOSPHAMIDE + DOXORUBICIN	4
DOXORUBICIN + STEPTOZOTOCIN	4
CYCLOPHOSPHAMIDE + ETOPOSIDE + LOMUSTINE + RITUXIMAB	4
RIALTO TRIAL	4
STS Non- Rhabdo EpSSG Vinblastine/MTX	4
ICE TRIAL	4
MVCARBO	4
CABAZITAXEL + CARBOPLATIN	4
CHOP + BORTEZOMIB	4
GDCVP	4
EPIRUBICIN + FLUOROURACIL	4
IDARUBICIN + MERCAPTOPURINE + METHOTREXATE + VINCRISTINE	4
PMITCEBO R	4
CYCLOPHOSPHAMIDE + DOXORUBICIN + PACLITAXEL	4
ARIEL 4 TRIAL	4
CISPLATIN + DACARBAZINE	4
BLEOMYCIN + OXALIPLATIN + VINCRISTINE	4
CNS Germ Cell Tumour II - Non Germinomatous - Standard PEI	4
ETOPOSIDE + IFOSFAMIDE + METHOTREXATE	4
METHOTREXATE + PROCARBAZINE + RITUXIMAB	4
CARBOPLATIN + DACARBAZINE	4
CNS Medbl St Risk <3yrs induction	4
CYCLOPHOSPHAMIDE + IDARUBICIN	4
ReBEL TRIAL	4
ACE	4
BLEOMYCIN + VINCRISTINE	4
CHLVPP/PABLOE	4
CYTARABINE + OXALIPLATIN	4
PHITT D Carboplatin/Doxorubicin	4
BLEOMYCIN + CYCLOPHOSPHAMIDE + ETOPOSIDE + GEMCITABINE +	4
BLEOMYCIN + CYCLOPHOSPHAMIDE + ETOPOSIDE + METHOTREXAT	4
DOXORUBICIN + PEGASPARGASE + VINCRISTINE	4
PAC-E	4
SIOP	4
BENDAMUSTINE + GEMCITABINE + RITUXIMAB + VINORELBINE	4
CYCLOPHOSPHAMIDE + CYTARABINE + POMALIDOMIDE	4
PHITT C CDDP-M	4
ADOC	4
CARBOPLATIN + DOXORUBICIN + MITOMYCIN	4
CARBOPLATIN + ETOPOSIDE + METHOTREXATE	4
CARBOPLATIN + THIOTEPA + TOPOTECAN	4
CYCLOPHOSPHAMIDE + VINBLASTINE	4
HERBY TRIAL	4
HLH Induction W3-8 Etoposide/Dexamethasone/Ciclosporin	4
CARBOPLATIN + ETOPOSIDE + THIOTEPA	4
ERIBULIN + TRASTUZUMAB	4
ALEMTUZUMAB + ETOPOSIDE + METHOTREXATE	4
PHITT D2 Carboplatin / etoposide	4
VIOLA TRIAL	4
BEVACIZUMAB + CARBO + DOCETAXEL	4
CISPLATIN + DOXORUBICIN + RITUXIMAB	4
BORTEZOMB + POMALIDOMIDE	4
EW Rel rEECur Ifosfamide	4
ALEMTUZUMAB + FLUDARABINE + THIOTEPA + TREOSULFAN	4
ALEMTUZUMAB + FLUDARABINE + TREOSULFAN	4
BLEOMYCIN + CISPLATIN + ETOPOSIDE + PACLITAXEL	4
CARBOPLATIN + CYCLO + DOXORUBICIN	4
CNS Ependymoma 99 vinc+cyclo+etop	4
CNS Medbl HR	4
CYCLOPHOSPHAMIDE + MITOXANTRONE + RITUXIMAB + VINCRISTINI	4
EPIRUBICIN + OXALIPLATIN + RALTITREXED	4
FLUOROURACIL + LOMUSTINE	4
PHITT C Carboplatin/Doxorubicin	4
PHITT E2 PLADO	4
WT2001	4
ASPARAGINASE + CYTARABINE + ETOPOSIDE + METHOTREXATE	4
BLEOMYCIN + CYCLOPHOSPHAMIDE + GEMCITABINE + RITUXIMAB +	4
CLADRIBINE + CYCLOPHOSPHAMIDE + RITUXIMAB	4
ESTRAMUSTINE	4
PACE-BOM	4
CARMUSTINE + CYCLOPHOSPHAMIDE + ETOPOSIDE	4
CHLORAMBUCIL + ETOPOSIDE + RITUXIMAB	4
CISPLATIN + CYCLOPHOSPHAMIDE	4
CYTARABINE + IDARUBICIN + METHOTREXATE + RITUXIMAB	4
DACTINOMYCIN + IFOSFAMIDE + VINCRISTINE	4
FMD	4
MAP + MIFAMURTIDE	4
MUK 11 TRIAL	4
CLOFARABINE + CYCLOPHOSPHAMIDE + ETOPOSIDE	4
CLOFARABINE	4
CYCLOPHOSPHAMIDE + DOXORUBICIN + METHOTREXATE + RITUXIM	4
ROVALPITUZUMAB TESIRINE	4
ALEMTUZUMAB + FLUDARABINE	4
CLADRIBINE + CYTARABINE + IDARUBICIN	4
HLH Consol Etop/Dexamthasone/Ciclosporin	4
CARBOPLATIN + PROCARBAZINE	4
CYTARABINE + FLUDARABINE + RITUXIMAB	4
CYTARABINE + IDARUBICIN	4
CYCLOPHOSPHAMIDE + MERCAPTOPURINE + METHOTREXATE + PEGASPARA	4
HELIOS TRIAL	4
IBRITUMOMAB TIUXETAN + RITUXIMAB + Y90	4
Idarubicin + Tretinoin	4
CYCLOPHOSPHAMIDE + DOCETAXEL + EPIRUBICIN + TRASTUZUMAB	4
DOXORUBICIN + METHOTREXATE + MIFAMURTIDE	4
IDARAM	4
BLEOMYCIN + CYCLOPHOSPHAMIDE + ETOPOSIDE + GEMCITABINE +	4
CARBOPLATIN + TEYSUNO	4
CISPLATIN + DOXORUBICIN + METHOTREXATE + MIFAMURTIDE	4
CVP R + ETOPOSIDE	4
STS Non-Rhabdo EpSSG 2005 Ifos	4
STS Non-Rhabdo EpSSG 2005 VA	4
CARBOPLATIN + CYCLOPHOSPHAMIDE + EPIRUBICIN + FLUOROURA	4
CCEP	4
CYCLOPHOSPHAMIDE + NAB-PACLITAXEL	4
DENOSUMAB + ERIBULIN	4
IFOSFAMIDE + VINCRISTINE	4
CISPLATIN + CYTARABINE	4
CYCLOPHOSPHAMIDE + DACTINOMYCIN	4
CYCLOPHOSPHAMIDE + ETOPOSIDE + THALIDOMIDE	4
MEAD (PEG-ASPARAGINASE)	4
R-PCE2BO (ETOPOPHOS¿½)	4
BEVACIZUMAB + CARBO	4
CARBOPLATIN + CETUXIMAB	4
CARMUSTINE	4
GEMCITABINE + OXALIPLATIN + PEGASPARAGINASE	4
METHOTREXATE + MITOMYCIN + MITOXANTRONE	4
AZACITIDINE	5
BEP	5
UKALL2014	5
CYTARABINE	5
TRIFLURIDINE + TIPIRACIL	5
CYCLOPHOSPHAMIDE	5
DA	5
FLAG + IDARUBICIN	5
FCR	5
AML19 TRIAL	5
FLAIR TRIAL	5
R IVAC	5
BEAM	5
MATRIX TRIAL	5
ARSENIC TRIOXIDE + TRETINOIN	5
ALEMTUZUMAB + FLUDARABINE + MELPHALAN	5
AML18 TRIAL	5
R CODOX-M/ R IVAC	5
R CODOX M	5
CYTARABINE + DAUNORUBICIN + GEMTUZUMAB OZOGAMICIN	5
MYELOMA XII TRIAL	5
RADIUM 223	5
LEAM	5
METHOTREXATE HIGH DOSE	5
ALEMTUZUMAB	5
CHOP R + METHOTREXATE	5
CYTARABINE HIGH DOSE	5
CYCLOPHOSPHAMIDE + FLUDARABINE	5
UKALL60+	5
ATG + BUSULFAN + FLUDARABINE	5
CYCLOPHOSPHAMIDE HIGH DOSE	5
NORDIC	5
AML17 TRIAL	5
UKALLR3	5
FBC	5
ALL INTERFANT 06	5
ALL INTERIM GUIDELINES	5
Yescarta + Fludarabine + Cyclophosphamide	5
CARBOPLATIN + ETOPOSIDE + IFOSFAMIDE + RITUXIMAB	5
STS RHABDO RMS 2005	5
ALEMTUZUMAB + CYCLO + FLUDARABINE	5
EW REL REECUR	5
STS RHABDO VIT0910	5
ARSENIC TRIOXIDE	5
APML	5
UKALL2003	5
ATG + CYCLOPHOSPHAMIDE TBI ALLO	5
ARSENIC TRIOXIDE + ATRA	5
ALL INTENSIFICATION	5
FLAG	5
POMB/ACE	5
CYTARABINE + RITUXIMAB	5
INOTUZUMAB OZOGAMICIN	5
AML16 TRIAL	5
AML 18 PILOT TRIAL	5
SMILE	5
BLEOMYCIN + CARBOPLATIN + ETOPOSIDE	5
MIDOSTAURIN	5
ALL PH+VE	5
ALEMTUZUMAB + BUSULFAN + CYCLOPHOSPHAMIDE	5
BLEOMYCIN + CARBOPLATIN + CISPLATIN + VINCRISTINE	5
LACE	5
CODOX M	5
CYTARABINE HD + METHOTREXATE HD	5
FAB LMB	5
ALL Interim Guide 2019 Induction A	5
LI-1 TRIAL	5
IDARUBICIN	5
AMSACRINE + ATG+ BUSULFAN + CYTARABINE + FLUDARABINE	5
AMSACRINE + ATG + BUSULFAN + CYTARABINE + FLUDARABINE	5
Kymriah + Fludarabine + Cyclophosphamide	5
DOXORUBICIN + HD MTX	5
BEAM + ALEMTUZUMAB	5
DA-EPOCH	5
DAUNORUBICIN + CYTARABINE + MIDOSTAURIN	5
ALL Interim Guide 2019 Augmented BFM Consolidation C	5
FLUDARABINE + MELPHALAN	5
ALEMTUZUMAB + PENTOSTATIN	5
IFOSFAMIDE + LIPOSOMAL DOXORUBICIN	5
ATG	5
CHOP-R + CYTARABINE HIGH DOSE	5
ALL MAINTENANCE	5
CYCLOPHOSPHAMIDE + CYTARABINE + DOXORUBICIN + RITUXIMAB	5
CISPLATIN + DACTINOMYCIN + ETOPOSIDE + FILGRASTIM + METHOT	5
ALEMTUZUMAB + CYCLOPHOSPHAMIDE + FLUDARABINE	5
AML DOWNS	5
ALL Interim Guide 2019 Delayed Intensification A	5
ACE (GERM CELL)	5
IDARUBICIN + TRETINOIN	5
ALL Interim Guide 2019 Maintenance	5
ALL Interim Guide 2019 Standard BFM Consolidation B	5
UKALL 60+ TRIAL	5
ALL Interim Guide 2019 Standard Interim Maintenance A	5
ALL Interim Guide 2019 Induction B	5
DAUNORUBICIN + PEGASPARAGINASE + VINCRISTINE	5
AML INTERIM GUIDELINES	5
CYTARABINE + METHOTREXATE + RITUXIMAB	5
ALL INDUCTION	5
FIGARO TRIAL	5
CYTARABINE + ETOPOSIDE + IFOSFAMIDE + METHOTREXATE + RITU	5
TRANSPLANT BEAM	5
GEMTUZUMAB OZOGAMICIN	5
DAUNORUBICIN + VINCRISTINE	5
TRANSPLANT CYTARABINE + ETOPOSIDE + LOMUSTINE + MELPHALA	5
MACE	5
TRANSPLANT ALEMTUZUMAB + FLUDARABINE + MELPHALAN	5
NELARABINE	5
UKALL XII	5
ALL R T-CELL CONSOLIDATION	5
METHOTREXATE + RITUXIMAB + TEMOZOLOMIDE	5
ADE	5
TRANSPLANT CYCLOPHOSPHAMIDE + FLUDARABINE (+/- TBI)	5
CARBOPLATIN + CYCLOPHOSPHAMIDE + ETOPOSIDE + PACLITAXEL	5
ALL CONSOLIDATION	5
ETOPOSIDE + IFOSFAMIDE + MTX	5
ROMAZA TRIAL	5
ALL Int Guide B BFMCons	5
CLOFARABINE + CYTARABINE	5
CYTARABINE IT + METHOTREXATE	5
FLA	5
ALL Int Guide A Ind	5
CYTARABINE + METHOTREXATE	5
ALL Interim Guide 2019	5
IDARUBICIN + MITOXANTRONE + TRETINOIN	5
CYTARABINE + FLUDARABINE	5
AZACITIDINE + VENETOCLAX	5
ALL Int GdeC D Int PhIV reind PAsp	5
BENDAMUSTINE + CYTARABINE + ETOPOSIDE + MELPHALAN	5
CYTARABINE + ETOPOSIDE + MITOXANTRONE	5
BUSULFAN + FLUDARABINE	5
CODOX M + IVAC	5
ALEMTUZUMAB + LEAM	5
BUSULFAN + CYCLOPHOSPHAMIDE	5
CYTARABINE + MIDOSTAURIN	5
ERWINIA ASPARAGINASE	5
TRANSPLANT ALEMTUZUMAB + CYTARABINE + ETOPOSIDE + LOMUS	5
111 TRIAL	5
AMSACRINE + BUSULFAN + CYTARABINE + FLUDARABINE	5
ALL Interim Guide 2019 Consolidation A	5
FLUDARABINE + THIOTEPA + TREOSULFAN	5
AML Relapsed FLA - Idarubicin	5
TRIFLURIDINE TIPIRACIL	5
AZACITIDINE + RUXOLITINIB	5
BLEOMYCIN + CISPLATIN + ETOPOSIDE + METHOTREXATE + VINCRIS	5
ATG + BUSULFAN + FLUDARABINE + THIOTEPA	5
IMATINIB + MERCAPTOPURINE + VINCRISTINE	5
ATG + CYCLOPHOSPHAMIDE + FLUDARABINE	5
BUSULFAN + MELPHALAN	5
CANTATA TRIAL	5
BLEOMYCIN + CISPLATIN + ETOPOSIDE	5
ATG + CYCLOPHOSPHAMIDE	5
BLEOMYCIN + CYCLOPHOSPHAMIDE + ETOPOSIDE + GEMCITABINE +	5
CNS ATRT Doxorubicin/Ifosfamide/Carboplatin/Etoposide/Methotrexate	5
CYCLOPHOSPHAMIDE + CYTARABINE + DAUNORUBICIN + MERCAPT	5
DOXORUBICIN + METHOTREXATE	5
ATG + FLUDARABINE	5
CAPECITABINE + LOMUSTINE	5
METHOTREXATE HIGH DOSE + RITUXIMAB	5
ALL Interim Guide 2019 Standard Interim Maintenance B	5
METHOTREXATE HD + VINCRISTINE	5
GILTERITINIB	5
BUSULFAN + CYCLOPHOSPHAMIDE + FLUDARABINE + THIOTEPA	5
CYTARABINE + Gemtuzumab Ozogamicin	5
MA	5
BEAM + FLUDARABINE	5
AMSACRINE + CYTARABINE	5
CYTARABINE + FLUDARABINE + IDARUBICIN	5
DAUNORUBICIN	5
TRANSPLANT ATG + FLUDARABINE + THIOTEPA + TREOSULFAN	5
GCLAC	5
BUSULFAN + FLUDARABINE + METHOTREXATE	5
IMATINIB + MERCAPTOPURINE + METHOTREXATE	5
ALL Int Guide B Int Maint	5
TRANSPLANT ALEMTUZUMAB + CYCLOPHOSPHAMIDE + FLUDARABIN	5
DOXORUBICIN + IFOSFAMIDE + VINCRISTINE	5
BUSULFAN + THIOTEPA	5
CLOFARABINE + CYTSARABINE + IDARUBICIN	5
ALL NOPHO	5
AMSACRINE + ATG + CYCLOPHOSPHAMIDE + CYTARABINE + FLUDAR	5
CISPLATIN + ETOPOSIDE + METHOTREXATE	5
ALL Interfant 06 ADE	5
ALEMTUZUMAB + FLUDARABINE + LEAM	5
ETOPOSIDE + MELPHALAN	5
IDARUBICIN + IMATINIB + VINCRISTINE	5
ALL Interim Guide 2019 Induction C	5
TRANSPLANT BUSULFAN + FLUDARABINE	5
ALL Interim Guide 2019 NOPHO C	5
CYCLOPHOSPHAMIDE + CYTARABINE + ETOPOSIDE + METHOTREXAT	5
CLADRIBINE + CYTARABINE + DAUNORUBICIN	5
TRANSPLANT AMSACRINE + ANTITHYMOCYTE IMMUNOGLOBULIN (R/	5
TRANSPLANT ATG + BUSULFAN + FLUDARABINE + THIOTEPA	5
CLADRIBINE + CYTARABINE	5
DE ANGELIS	5
FORODESINE	5
ATG + FLUDARABINE + MELPHALAN	5
ATG + FLUDARABINE + THIOTEPA + TREOSULFAN	5
TRANSPLANT FLUDARABINE + THIOTEPA + TREOSULFAN	5
CARBOPLATIN + CYTARABINE + ETOPOSIDE	5
CYCLOPHOSPHAMIDE + ETOPOSIDE + NELARABINE	5
DECITABINE	5
MIDAC	5
BLEOMYCIN + CYCLOPHOSPHAMIDE + ETOPOSIDE + GEMCITABINE +	5
BLEOMYCIN + CYCLOPHOSPHAMIDE + ETOPOSIDE + GEMCITABINE +	5
CYTARABINE + VINCRISTINE	5
IDARUBICIN + METHOTREXATE + VINCRISTINE	5
MTX HD + PROCARBAZINE + VINCRISTINE	5
BEAM R	5
BUSULFAN + CYCLOPHOSPHAMIDE + FLUDARABINE	5
CARBOPLATIN + CYTARABINE + RITUXIMAB	5
CYTARABINE + ETOPOSIDE + IMATINIB	5
CYTARABINE + ETOPOSIDE + METHOTREXATE	5
ALL/LBL T Cell Relapse Intensification	5
BLEOMYCIN + CYCLOPHOSPHAMIDE + ETOPOSIDE + MITOXANTRONE	5
CARBOPLATIN + ETOPOSIDE + IFOSFAMIDE + PACLITAXEL	5
CISPLATIN + CYCLOPHOSPHAMIDE + CYTARABINE + DOXORUBICIN +	5
DAUNORUBICIN + METHOTREXATE + PEGASPARAGINASE + VINCRIS	5
CNS HGG CISPLATIN/ETOPOSIDE	5
CYTARABINE + METHOTREXATE HD	5
ALL UKALL2011 TRIAL + ALL UKALL2011 Intensification MTX	5
TRANSPLANT ATG + ETOPOSIDE (+/- TBI)	5
ALL/LBL T Cell Relapse Consolidation	5
YTTRIUM-90	5
ALL INTENSIFICATION MTX	5
AML RELAPSED	5
CAMELLIA TRIAL	5
SPANISH MAINTENANCE	5
ALL/LBL T Cell Relapse NECTAR Cyclophosphamide, etoposide, nelarabine and IT	5
HORMONES	1
CYTARABINE + METHOTREXATE + PEGASPARAGINASE	5
CHOP R + LIPOSOMAL DOXORUBICIN	3
CYCLOPHOSPHAMIDE + DACTINOMYCIN + DOXORUBICIN + VINCRISTINE	5
TRIAL	6
CYCLOPHOSPHAMIDE + FLUDARABINE + MITOXANTRONE	3
CYCLOPHOSPHAMIDE + LIPOSOMAL DOX	3
VELIPARIB	1
CARBOPLATIN + IRINOTECAN	3

For peer review only

BMJ Open

A common methodology for validation of the Qcovid algorithm across the four UK nations

Journal:	*BMJ Open*
Manuscript ID	bmjopen-2021-050994.R1
Article Type:	Protocol
Date Submitted by the Author:	07-Dec-2021
Complete List of Authors:	Kerr, Steven; The University of Edinburgh Usher Institute of Population Health Sciences and Informatics,<br>Robertson, Chris; University of Strathclyde, Department of Mathematics and Statistics<br>Nafilyan, Vahe; Office for National Statistics<br>Lyons, Ronan; University of Wales Swansea, Swansea Clinical School<br>Kee, Frank; Queen's University Belfast, UKCRC Centre of Excellence for Public Health (NI)<br>Cardwell, Christopher; Queen's University Belfast, School of Medicine, Dentistry and Biomedical Sciences<br>Coupland, Carol; University of Nottingham, Division of Primary Care<br>Lyons, Jane; Swansea University Medical School<br>Humberstone, Ben; Office for National Statistics<br>Hippisley-Cox, Julia; University of Oxford, Nuffield Department of Primary Care Sciences<br>Sheikh, Aziz; University of Edinburgh, Division of Community Health Sciences
**Primary Subject Heading**:	Epidemiology
Secondary Subject Heading:	Infectious diseases, Respiratory medicine
Keywords:	COVID-19, Epidemiology < INFECTIOUS DISEASES, Public health < INFECTIOUS DISEASES

SCHOLARONE™
Manuscripts

A common methodology for validation of the QCOVID algorithm across the four UK nations

Steven Kerr, stevenkerr2@gmail.com (corresponding author) [1]
Chris Robertson [2,3]
Vahe Nafilyan [4]
Ronan A Lyons [5]
Frank Kee [6]
Chris Cardwell [6]
Carol Coupland [7]
Jane Lyons [5]
Ben Humberstone [4]
Julie Hippisley-Cox [8]
Aziz Sheikh [1]

[1] Usher Institute, The University of Edinburgh, NINE Edinburgh BioQuarter, Edinburgh, UK, EH16 4UX.
[2] University of Strathclyde, Glasgow, UK.
[3] Public Health Scotland, UK
[4] Office for National Statistics, UK.
[5] Population Data Science, Swansea University Medical School, Swansea, UK.
[6] Queen's University Belfast, Belfast, UK.
[7] University of Nottingham, Nottingham, UK.
[8] University of Oxford, Oxford, UK.

Keywords: Covid-19, Coronavirus, QCOVID, Epidemiology, Public Health.

Word count: 3,148

For peer review only

ABSTRACT

Introduction:

The QCOVID algorithm is a risk prediction tool for infection and subsequent hospitalisation/death due to SARS-Cov-2. At the time of writing, it is being used in important policymaking decisions by the UK and devolved governments for combatting the Covid-19 pandemic, including deliberations on shielding and vaccine prioritisation. There are four statistical validations exercises currently planned for the QCOVID algorithm, using data pertaining to England, Northern Ireland, Scotland and Wales respectively. This paper presents a common procedure for conducting and reporting on validation exercises for the QCOVID algorithm.

Methods and Analysis:

We will use open, retrospective cohort studies to assess the performance of the QCOVID risk prediction tool in each of the four UK nations. Linked datasets comprising of primary and secondary care records, virological testing data and death registrations will be assembled in trusted research environments in England, Scotland, Northern Ireland and Wales. We will seek to have population level coverage as far as possible within each nation. The following performance metrics will be calculated by strata: Harrell's C, Brier score, $R^{2,}$ and Royston's D.

Ethics and dissemination:

Approvals have been obtained from relevant ethics bodies in each UK nation. Findings will be made available to national policymakers, presented at conferences and published in peer reviewed journal

Strengths and limitations of this study:

- We will use national level data within each UK nation
- There are potential issues with missing data and differences in the way data is recorded in each country.
- We will evaluate the performance of the algorithm according to several relevant metrics.

For peer review only

INTRODUCTION:

The QCOVID algorithm [1] has been developed to help identify adults at high risk of being hospitalised or dying following infection with SARS-Cov-2 (Severe acute respiratory syndrome coronavirus 2). The algorithm takes as input a total of 40 variables including age, sex, ethnicity, Townsend deprivation score [2] and housing category, as well as clinical information including body mass index (BMI) and 33 variables related to medical conditions and treatments. It outputs the predicted probability that an individual will be infected with SARS-Cov-2 and then hospitalised, and the predicted probability that an individual will be infected with SARS-Cov-2 and then die, over a 90 day period. The algorithm was trained using information from the QResearch database [3], which as of April 2020 contained routinely collected data from 1205 General Practices across England, covering 10.5 million patients. The initial training dataset comprised of a cohort of 6.08 million individuals tracked from the 24 January 2020 to 30 April 2020, and was validated on a subset of 2.17 million individuals tracked from 1 May 2020 to 30 June 2020. The research protocol for the development of the QCOVID algorithm can be found in [4].

The QCOVID algorithm was commissioned by the Chief Medical Officer for England on behalf of the UK government. The algorithm has been used to inform UK and devolved government policy on combatting the SARS-Cov-2 pandemic, including guidance on social-distancing and shielding measures, as well vaccine prioritisation. [5] It is therefore of great importance to validate the predictions of the algorithm in sub-populations of the UK that were not in the initial training set, but will potentially be subject to those policies.

At the time of writing, there are validation exercises planned in Scotland, Northern Ireland and Wales, and a validation exercise underway in England. Validation work has been expedited in order to support national decision making. In order to facilitate useful comparison of the results of the separate validation exercises, it is necessary to establish a consistent set of procedures. The purpose of this paper is to explicate a common methodology for the validation of the QCOVID algorithm across the four nations of the UK.

METHODS AND ANALYSIS:

Study design:

For peer review only

Open, retrospective cohort study designs will be employed, making use of routinely collected data from General Practices for clinical and demographic information, as well as linked datasets on hospital admissions, reverse-transcription polymerase chain reaction (RT-PCR) testing for Covid-19, and registered deaths. We will aim to have national coverage as far as is possible within each of the four nations of the UK.

Data Sources:

Box 1 contains a brief summary of the main datasets that will be used in the validation exercise for each nation

Box 1: Main datasets to be used

England: Office for National Statistics (ONS) Public Health Linked Data Asset. This dataset is based on the 2011 Census in England covering 40.1 million people, linked at individual level using the NHS number to mortality records, Hospital Episode Statistics (HES) and the General Practice Extraction Service (GPES) data for pandemic planning and research. The data covers 80% of the population of England aged 19 and over.

Northern Ireland: National Health Application and Infrastructure Services (NHAIS) will be used for demographic information. The Patient Administration System (PAS) will be used for data on hospital admissions. Death data will be drawn from the Registrar General, and identified as Covid-19 related through the official Northern Ireland Statistics and Research Agency (NISRA) dashboard. The General Practice Information Platform (GPIP) will bring together GP records from practices across Northern Ireland into a single dataset for use in the validation. As this is not held in the Honest Broker Service, a separate request to its governance board is being made. The Electronic Prescribing Database (EPD) will be used to access information on prescriptions.

Scotland: EAVE II dataset [6]. Contains primary health care records for 5.4 million people covering 99% of the population of Scotland, linked with secondary care data from Scottish Morbidity Record (SMR), Covid-19 test results from Electronic Communication of Surveilance Scotland (ECOSS), and mortality data from National Records Scotland.

Wales: Secure Anonymised Information Linkage (SAIL system) [7]. This will utilise the Controlling Covid (ConCOV) platform linking records on 3.2 million people from the NHS population spine with hospital (Patient Episode Database for Wales), Welsh Longitudinal GP record (WLGP), Covid-19 test results from the Laboratory Information Management System (LIMS), and mortality and 2011Census data from the Office for National Statistics (ONS) [8]

For peer review only

Selection criteria:

Any individual in the relevant linked dataset between the ages of 19 and 100 will be included. Individuals who had an event (hospitalisation or death) in the first period (24 January 2020 – 30 April 2020) will be excluded from any analysis in the second period (1 May 2020 – 30 June 2020).

Exposure and Outcomes:

Table 1 and 2 list all exposure and outcomes variables respectively for the QCOVID algorithm, along with a description, variable type (e.g. integer, real, categorical) and possible values.

Table 1: Exposure variables in QCOVID algorithm.

**Variable:**	**Description/Question:**	**Value:**
**Demographic:**		
age	Age in years	Integer: 19-100
sex	Biological sex at birth	Categorical: female, male
town	Townsend Deprivation Score	Real number
ethnicity	Ethnicity	Categorical: White, Indian, Pakistani, Bangladeshi, Other Asian, Caribbean, Black African, Chinese, other ethnic group
homecat	What is your housing category - care home or homeless or neither?	Categorical: neither, care home, homeless

**Clinical:**		
bmi	Body Mass Index (kg/m^2)	Positive real number
chemocat	Have you had chemotherapy in the last 12 months?	Categorical: none, group A, group B, group C

learncat	Do you have a learning disability or Down's Syndrome?	Categorical: learning disability, Down syndrome
renalcat	Chronic Kidney Disease (CKD) stage	Categorical: No serious kidney disease, CKD stage 3, CKD stage 4, CKD stage 5 without dialysis or transplant, CKD stage 5 with dialysis in last 12 months, CKD stage 5 with transplant
diabetescat	Do you have diabetes?	Categorical: none, type 1, type 2
b2_82	Have you been prescribed immunosuppressants four or more times in the previous 6 months?	Categorical: yes, no
b2_leukolaba	Have you been prescribed anti-leukotriene or long acting beta2-agonists (LABA) four or more times in the previous 6 months?	Categorical: yes, no
b2_prednisone	Have you been prescribed oral predisolone containing preparations prescribed four or more times in the previous 6 months?	Categorical: yes, no
b_AF	Do you have atrial fibrillation?	Categorical: yes, no
b_CCF	Do you have heart failure?	Categorical: yes, no
b_asthma	Do you have asthma?	Categorical: yes, no
b_bloodcancer	Have you a cancer of the blood or bone marrow such as leukaemia, myelodysplastic syndromes,	Categorical: yes, no

For peer review only

	lymphoma or myeloma and are at any stage of treatment?	
b_cerebralpalsay	Do you have cerebral palsy?	Categorical: yes, no
b_chd	Do you have coronary heart disease?	Categorical: yes, no
b_cirrhosis	Do you have cirrhosis of the liver?	Categorical: yes, no
b_congenheart	Do you have congenital heart disease or have you had surgery for it in the past?	Categorical: yes, no
b_copd	Do you have chronic obstructive pulmonary disease (COPD)?	Categorical: yes, no
b_dementia	Do you have dementia?	Categorical: yes, no
b_epilepsy	Do you have epilepsy?	Categorical: yes, no
b_fracture4	Have you had a prior fracture of hip, wrist, spine or humerus?	Categorical: yes, no
b_neurorare	Do you have motor neurone disease, multiple sclerosis, myasthenia, or Huntingtons's Chorea?	Categorical: yes, no
b_parkinsons	Do you have Parkinson's disease?	Categorical: yes, no
b_pulmhyper	Do you have pulmonary hypertension or pulmonary fibrosis?	Categorical: yes, no
b_pulmrare	Do you have cystic fibrosis or bronchiectasis or alveolitis?	Categorical: yes, no
b_pvd	Do you have peripheral vascular disease?	Categorical: yes, no
b_ra_sle	Do you have rheumatoid arthritis or SLE?	Categorical: yes, no
b_respcancer	Do you have lung or oral cancer?	Categorical: yes, no
b_semi	Do you have severe mental illness?	Categorical: yes, no

For peer review only

b_sicklecelldisease	Do you have sickle cell disease or severe combined immune deficiency syndromes?	Categorical: yes, no
b_stroke	Have you had a stroke or TIA?	Categorical: yes, no
b_vte	Have you had a thrombosis or pulmonary embolus?	Categorical: yes, no
p_marrow6	Have you had a bone marrow or stem cell transplant in the last 6 months?	Categorical: yes, no
p_radio6	Have you had radiotherapy in the last 6 months?	Categorical: yes, no
p_solidtransplant	Have you had a solid organ transplant (lung, liver, stomach, pancreas, spleen, heart or thymus)?	Categorical: yes, no

Table 2: Outcomes variables in QCOVID algorithm.

**Variable:**	**Description/Question:**	**Value:**
Time to Covid-19 hospitalisation	Time to hospitalisation with RT-PCR confirmed Covid-19 infection in the cohort period in days.	Real number: 0-91
Time to Covid-19 death	Time to death with Covid-19 confirmed or suspected on their death certificate, or confirmed by RT-PCR test, in the cohort period in days.	Real number: 0-91

Whenever available, all variables will be taken as the most recent recorded value in the relevant dataset at the date of entry into the cohort. The Townsend Deprivation Score (TDS) will be determined by matching available residential location information with output area and the

For peer review only

corresponding TDS from the 2011 UK census [9]. Categories for the variable chemocat will be determined using the lookup table in the supplemental materials.

Data cleaning:

The following procedures will be used for data cleaning:

- **diabetes_cat:** If the most recent entry has both type 1 and types 2 recorded, diabetes_cat will be set to type 2.
- **BMI:** The most recently recorded patient BMI within the last 5 years. If the most recently recorded BMI is from more than 5 years ago at the search date, BMI will be set to missing value. Implausible values for BMI (<12 or >70) will be set to missing value.
- **learncat:** If a patient is recorded has having both learning disability and Down's syndrome, learncat will be set to Down's syndrome.

Missing data:

For comorbidities and medication use and treatments, missing values will be taken to mean absence of that factor. Modal substitution will be considered for missing values for ethnicity. For any other missing values of predictor variables, a single imputation will be considered. Outcome variables will not be imputed. The following methods may be considered for use in the imputation: predictive mean matching, least squares, logistic and multinomial models, imputation by chained equations.

Statistical Analysis:

Each validation exercise will report a table of cohort characteristics, following Table 2 in [1]. The main performance metrics that will be calculated are R^2 [9], Harrell's C, Royston's D [10] and the Brier score. Different stratifications for these statistics will be considered, including by age, sex and time period. 95% confidence intervals will be reported for R^2, Harrell's C and Royston's D. Graphs of observed and predicted probability of hospital admission and death by vigintile for stratified subgroups will be reported, following [1].

Sample Size:

A preliminary sample size calculation can be done using figures from the original paper [1]. Using the estimated standard deviation of Harrell's C for females in the first time period and assuming Harrell's C is asymptotically normally distributed implies that a sample size of approximately 5,714

For peer review only

would be sufficient to detect a true value for Harrell's C that is greater than or equal to 0.8 with 80% power. Repeating this calculation for other population subgroups and time periods yields results of a similar magnitude. The samples sizes in the planned studies will be on the order of hundreds of thousands or millions.

Ethics, reporting and dissemination

The ethics approval for the development and validation of QCOVID in England was granted by the East Midlands-Derby Research Ethics Committee [reference 18/EM/0400]. For Scotland, approvals have been obtained by the National Research Ethics Service Committee (REC), South East Scotland 02 (REC number: 12/SS/0201) and the Public Benefit and Privacy Panel for Health and Social Care (reference number: 1920-0279). The data to be used in this study for Wales are available in the SAIL Databank at Swansea University, Swansea, UK. All proposals to use SAIL data are subject to review by an independent Information Governance Review Panel (IGRP). Before any data can be accessed, approval must be given by the IGRP. The IGRP gives careful consideration to each project to ensure proper and appropriate use of SAIL data. When access has been approved, it is gained through a privacy-protecting safe haven and remote access system referred to as the SAIL Gateway. SAIL has established an application process to be followed by anyone who would like to access data via SAIL.[7] Findings will be presented at conferences, published in peer-reviewed journals and to the funders and government COVID-19 advisory bodies as appropriate. Strengthening the Reporting of Observational Studies in Epidemiology (STROBE) and Reporting of studies Conducted using Observational Routinely-collected Data (RECORD) (via the COVID-19 extension) checklists will guide our study findings reporting. The Northern Ireland validation study proposal is under review by the NITRE for HSC data accessed via Northern Ireland Honest Broker Service; an Ethics application has been submitted through IRAS.

Author's Contributions:

AS conceived this protocol. CR, VH, FK, TC, JHC, BH, CC, RAL and JL provided country specific information about available data and analysis plans. SK wrote drafts of this protocol. All authors gave final approval of the version to be published.

Acknowledgments

This work will use data provided by patients and collected by a number of organisations. We would like to acknowledge all patients who shared their information as well as all data providers who make anonymised data available for research. In particular, Public Health Scotland, Public Health

For peer review only

Wales, Public Health England, the NHS, the SAIL databank, and the Office for National Statistics.

Funding:

The validation in England will be funded by a grant from the National Institute for Health Research following a commission by the Chief Medical Officer for England. In Scotland, EAVE II is funded by the Medical Research Council [MR/R008345/1] and supported by the Scottish Government. In Wales, ConCOV is supported by the Medical Research Council [MR/V028367/1].

Competing interests:

AS reports grants from NIHR, grants from MRC, and grants from HRR UK, during the conduct of the study. JL and RAL report grants from UKRI Medical Research Council, during the conduct of the study. JHC reports grants from John Fell Oxford University Press Research Fund, grants from Cancer Research UK (CR-UK) grant number C5255/A18085, through the Cancer Research UK Oxford Centre, grants from the Oxford Wellcome Institutional Strategic Support Fund (204826/Z/16/Z), grants from NIHR, during the conduct of the study; personal fees and other from ClinRisk Ltd, outside the submitted work; and JHC is an unpaid director of QResearch, a not-for-profit organisation which is a partnership between the University of Oxford and EMIS Health who supply the QResearch database used for this work. Carol Coupland reports personal fees from ClinRisk ltd, outside the submitted work. JHC, AS, and Carol Coupland were members of the research team involved in the development of the QCOVID risk prediction algorithm. All other authors report no conflict of interest.

Patient and Public Involvement:

There are no plans for Patient and Public Involvement in this research.

Data sharing:

All code used in these analyses will be made publicly available online e.g. through GitHub.

References:

1. Clift AK, Coupland CAC, Keogh RH, Diaz-Ordaz K, Williamson E, Harrison EM, Hayward A, Hemingway H, Horby P, Mehta N, Benger J, Khunti K, Spiegelhalter D, Sheikh A, Valabhji J, Lyons RA, Robson J, Semple MG, Kee F, Johnson P, Jebb S, Williams T, Hippisley-Cox J. Living risk prediction algorithm (QCOVID) for risk of hospital admission and mortality from coronavirus 19 in adults: national derivation and validation cohort study. BMJ. 2020 Oct 20;371:m3731. doi: 10.1136/bmj.m3731. PMID: 33082154; PMCID: PMC7574532.

For peer review only

2. Townsend, P., Phillimore, P. and Beattie, A. (1988) Health and Deprivation: Inequality and the North. Routledge, London. doi: 10.7748/ns.2.17.34.s66. PMID: 27415096.

3. Qresearch, https://www.qresearch.org/

4. Hippisley-Cox J, Clift AK, Coupland CAC, et al. Protocol for the development and evaluation of a tool for predicting risk of short-term adverse outcomes due to COVID-19 in the general UK population. *MedRxiv* 2020:2020.06.28.20141986-2020.06.28

5. https://www.gov.uk/government/news/new-technology-to-help-identify-those-at-high-risk-from-covid-19

6. EAVE II, The Usher Institute, https://www.ed.ac.uk/usher/eave-ii

7. The Secure Anonymised Information Linkage (SAIL) databank. Available at: https://saildatabank.com

8. Lyons J, Akbari A, Torabi F, Davies G, North L, Griffiths R, Bailey R, Hollinghurst J, Fry R, Turner S, Thompson D, Rafferty J, Mizen A, Orton C, Ellwood-Thompson S, Au-Yeung L, Cross L, Gravenor M, Brophy S, Lucini B, John A, Szakmany T, Davies J, Davies C, Williams C, Emmerson C, Cottrell S, Connor T, Taylor C, Pugh R, Diggle PJ, John G, Scourfield S, Hunt J, Cunningham AM, Helliwell K, Lyons RA. (2020) Understanding and responding to COVID19 in Wales: protocol for a privacy protecting data platform for enhanced epidemiology and evaluation of interventions. BMJ Open 2020;10:e043010. doi:10.1136/ bmjopen-2020-043010

9. 2011 UK Census, Townsend Deprivation Scores, https://www.statistics.digitalresources.jisc.ac.uk/dataset/2011-uk-townsend-deprivation-scores

10. Royston P, Sauerbrei W. A new measure of prognostic separation in survival data. Stat Med. 2004 Mar 15;23(5):723-48. doi: 10.1002/sim.1621. PMID: 14981672.

For peer review only

Sheet3b - Chemotherapy lookup

final classification	map
Group A	1 & 2
Group B	3 & 4
Group C	5

Bench Mark Group	Group
TRASTUZUMAB	1
ZOLEDRONIC ACID	1
DENOSUMAB	1
PEMBROLIZUMAB	1
PERTUZUMAB + TRASTUZUMAB	1
ENZALUTAMIDE	1
ABIRATERONE	1
NIVOLUMAB	1
CETUXIMAB	1
ATEZOLIZUMAB	1
BCG	1
BEVACIZUMAB	1
AFATINIB	1
FULVESTRANT	1
IPILIMUMAB + NIVOLUMAB	1
GOSERELIN	1
METHOTREXATE INTRATHECAL	1
PANITUMUMAB	1
LANREOTIDE	1
DURVALUMAB	1
PERTUZUMAB	1
PAMIDRONATE	1
MITOMYCIN INTRAVESICULAR	1
OCTREOTIDE	1
IMMUNOGLOBULIN	1
ANAGRELIDE	1
LETROZOLE	1
AVELUMAB	1
TRIPLE INTRATHECAL	1
CETUXIMAB + RT	1
BICALUTAMIDE	1
TRIPTORELIN	1
IPILIMUMAB	1
PRISM TRIAL	1
CEMIPLIMAB	1
NERATINIB	1
ANASTROZOLE	1
VISMODEGIB	1
IBANDRONIC ACID	1
TAMOXIFEN	1
CYTARABINE INTRATHECAL	1
EXEMESTANE	1
CYPROTERONE	1
TALIMOGENE LAHERPAREPVEC	1
LEUPRORELIN	1
DEGARELIX	1
ELOQUENT2 TRIAL	1
SILTUXIMAB	1
ELOQUENT TRIAL	1
BICALUTAMIDE + LEUPRORELIN	1
CONFIRM TRIAL	1
FAKTION TRIAL	1
APALUTAMIDE	1
EPIRUBICIN INTRAVESICULAR	1
STILBOESTROL	1
DARBEPOETIN	1
FLUOROURACIL + PANITUMUMAB	1
AVAPRITINIB	1
FURVA TRIAL	1
PERM TRIAL	1
ENZARAD TRIAL	1
BICALUTAMIDE + GOSERELIN	1
NB 13 CIS-RA (ISOTRETINOIN)	1
VORINOSTAT	1
ABIRATERONE + ENZALUTAMIDE	1
PARAGON TRIAL	1
MEDROXYPROGESTERONE	1
KESTREL TRIAL	1
MEGESTROL	1
XILONIX	1
RAMUCIRUMAB	1
CAM-PLEX TRIAL	1
BICALUTAMIDE + TAMOXIFEN	1
LATITUDE TRIAL	1
IPILIMUMAB + PEMBROLIZUMAB	1
QUADRUPLE INTRATHECAL	1
GOSERELIN + LEUPRORELIN	1
MERCAPTOPURINE + VINCRISTINE	1
PROGESTERONE	1
CLODRONIC ACID	1
CYPROTERONE + GOSERELIN	1
GOSERELIN + LETROZOLE	1
CISPLATIN + GEMCITABINE	2
HYDROXYCARBAMIDE	2
CAPECITABINE	2
CISPLATIN + RT	2
PALBOCICLIB	2
FLUOROURACIL	2
CISPLATIN	2
CAPECITABINE + IRINOTECAN	2
CISPLATIN + PEMETREXED	2
DABRAFENIB + TRAMETINIB	2
CAPECITABINE + GEMCITABINE	2
LETROZOLE + PALBOCICLIB	2
PEMETREXED	2
VINORELBINE	2
CISPLATIN + FLUOROURACIL	2
EVEROLIMUS	2
PAZOPANIB	2
SUNITINIB	2
ABEMACICLIB + FULVESTRANT	2
CABOZANTINIB	2
HCX	2
NIRAPARIB	2
CAPECITABINE + RT	2
PEMBROLIZUMAB + PEMETREXED	2
METHOTREXATE	2
GEFITINIB	2
OSIMERTINIB	2
ERLOTINIB	2
RIBOCICLIB	2
FLUOROURACIL + MITOMYCIN + RT	2
OLAPARIB	2
SORAFENIB	2
VINBLASTINE	2
AXITINIB	2
ALECTINIB	2
CAPECITABINE + CISPLATIN + RT	2
CISPLATIN + FLUOROURACIL + RT	2
TIVOZANIB	2
CRIZOTINIB	2
CAPECITABINE + MITOMYCIN	2
CAPECITABINE + VINORELBINE	2
DABRAFENIB	2
ABEMACICLIB	2
LENVATINIB	2
LETROZOLE + RIBOCICLIB	2
CAPECITABINE + MITOMYCIN + RT	2
BEXAROTENE	2
MITOMYCIN	2
INTERFERON	2
PONATINIB	2
VINCRISTINE	2
BUSULPHAN	2
CERITINIB	2
CAPECITABINE + TRASTUZUMAB	2
FLUOROURACIL + MITOMYCIN	2
NINTEDANIB	2
FLUOROURACIL + RT	2
REGORAFENIB	2
ANASTROZOLE + PALBOCICLIB	2
CETUXIMAB + CISPLATIN + FU	2
EVEROLIMUS + LENVATINIB	2
BRIGATINIB	2
LORLATINIB	2
CETUXIMAB + MDG	2
DICE TRIAL	2
BEVACIZUMAB + CAPECITABINE	2
TRASTUZUMAB + VINORELBINE	2
Fulvestrant + Palbociclib	2
Abemaciclib + Letrozole	2
ACELARATE TRIAL	2
VANDETANIB	2
EXEMESTANE + PALBOCICLIB	2
CISPLATIN + TRASTUZUMAB	2
INTERIM TRIAL	2
TRAMETINIB	2
UNIRAD TRIAL	2
MITOXANTRONE	2
CAPECITABINE + LAPATINIB	2
ICON9 TRIAL	2
ATEZOLIZUMAB + BEVACIZUMAB	2
COSMIC TRIAL	2
STAR TRIAL	2
RE-AKT TRIAL	2
BEACON TRIAL	2
DACOMITINIB	2
COLUMBUS TRIAL	2
PACKER MAINTENANCE	2
CAPECITABINE + NERATINIB	2
ANAGRELIDE + HYDROXYCARBAMIDE	2
INTERFERON BETA	2
CISPLATIN + FLUOROURACIL + TRASTUZUMAB	2
QUIZARTINIB	2
ASCIMINIB	2
PAKT TRIAL	2
METHOTREXATE + PEGASPARGASE	2
POLEM TRIAL	2
CISPLATIN + FLUORO + STREPTOZOCIN	2
CAP + RT	2
COMBI-AD TRIAL	2
OLYMPIA TRIAL	2
SPIRIT TRIAL	2
CEDIRANIB	2
FLUOROURACIL + INTERFERON ALFA	2
MILLENNIUM C16021 TRIAL	2
ENCORAFENIB	2
TALAZOPARIB	2
EVEROLIMUS + EXEMESTANE	2
CALYPSO TRIAL	2
INTERFERON ALFA + ZIDOVUDINE	2
IVOSIDENIB	2
SOLO1 TRIAL	2
LU-177	2
MITOMYCIN + RALTITREXED	2
NIMRAD TRIAL	2
SIROLIMUS	2
METHOTREXATE + TRASTUZUMAB	2
PALLET TRIAL	2
TOPARP TRIAL	2
TIPIFARNIB	2
RITUXIMAB + VINBLASTINE	2
IMATINIB + HYDROXYCARBAMIDE	2
METHOTREXATE + VINCRISTINE	2
BELLE3 TRIAL	2
GOSERELIN + PALBOCICLIB	2
PAZ02 TRIAL	2
PIN TRIAL	2
LAPATINIB	2
CASPS TRIAL	2
CISPLATIN + IMATINIB	2
TEMSIROLIMUS	2
LURBINECTIDIN	2
ERDAFITINIB	2
TIMELY TRIAL	2
COBIMETINIB	2
MASITINIB	2
BINIMETINIB	2
CREATE TRIAL	2
IRINOTECAN + MDG	3
OXALIPLATIN + MDG	3
CAPECITABINE + OXALIPLATIN	3
CETUXIMAB + IRINOTECAN + MDG	3
FLUOROURACIL + IRINOTECAN + OXALIPLATIN	3
CVD	3
RITUXIMAB	3
BORTEZOMIB + THALIDOMIDE	3
DARATUMUMAB	3
LENALIDOMIDE	3
IXAZOMIB + LENALIDOMIDE	3
BORTEZOMIB + DARATUMUMAB	3
IMATINIB	3
IBRUTINIB	3
CVP R	3
BORTEZOMIB	3
CISPLATIN + VINORELBINE	3
CABAZITAXEL	3
GEMCITABINE	3
CISPLATIN + ETOPOSIDE	3
MPV	3
TEMOZOLOMIDE	3
TRASTUZUMAB EMTANSINE	3
CETUXIMAB + OXALIPLATIN + MDG	3
CTD	3
OXALIPLATIN + MDG + PANITUMUMAB	3
ECX	3
RUXOLITINIB	3
CARFILZOMIB	3
IRINOTECAN + MDG + PANITUMUMAB	3
POMALIDOMIDE	3
IRINOTECAN + PANITUMUMAB + MDG	3
NILOTINIB	3
CAPECITABINE + CISPLATIN	3
CARDAMON TRIAL	3
BORTEZOMIB + PANOBINOSTAT	3
CYCLOPHOSPHAMIDE + LENALIDOMIDE	3
CVP + OBINUTUZUMAB	3
DASATINIB	3
TEMOZOLOMIDE + RT	3
MUK 9 TRIAL	3
IRINOTECAN	3
CYCLOPHOSPHAMIDE + RITUXIMAB + VINCRISTINE	3
VENETOCLAX	3
CYCLOPHOSPHAMIDE + RITUXIMAB	3
CARBO + FLUOROURACIL	3
CISPLATIN + GEMCITABINE + RITUXIMAB	3
OXALIPLATIN + RALTITREXED	3
CISPLATIN + PEMBROLIZUMAB + PEMETREXED	3
PEG INTERFERON	3
BOSUTINIB	3
OBINUTUZUMAB	3
CAPECITABINE + CETUXIMAB + IRINOTECAN	3
MPT	3
CISPLATIN + ETOPOSIDE + RT	3
IXAZOMIB	3
THALIDOMIDE	3
ETOPOSIDE	3
CAPECITABINE + TEMOZOLOMIDE	3
IRINOTECAN + TEMOZOLOMIDE	3
MERCAPTOPURINE + METHOTREXATE	3
BINIMETINIB + ENCORAFENIB	3
GEMCITABINE + OXALIPLATIN	3
BLINATUMOMAB	3
CMF	3
BEVACIZUMAB + OXALIPLATIN + MdG	3
BEVACIZUMAB + IRINOTECAN + MdG	3
CONCEPT TRIAL	3
OLARATUMAB	3
RALTITREXED	3
CETUXIMAB + CISPLATIN + FLUOROURACIL	3
GEMCITABINE + RT	3
IDELALISIB	3
BEVACIZUMAB + IRINOTECAN	3
BEVACIZUMAB + IRINOTECAN + MDG	3
IRINOTECAN + TEMOZOLOMIDE + VINCRISTINE	3
BOSTON TRIAL	3
CAPE + OXALIPLATIN + TRASTUZUMAB	3
VEMURAFENIB	3
IPM	3
CYCLOPHOSPHAMIDE + ETOPOSIDE	3
PENTOSTATIN	3
IPO	3
CYCLOPHOSPHAMIDE + MTX	3
PANOBINOSTAT	3
OXALIPLATIN	3
IRINOTECAN + BEVACIZUMAB	3
MITOTANE	3
FOCUS4 TRIAL	3
CYCLOPHOSPHAMIDE + VINCRISTINE	3
CAPECITABINE + CETUXIMAB + OXALIPATIN	3
CETUXIMAB + IRINOTECAN	3
COMPLEMENT TRIAL	3
AFLIBERCEPT + FU + IRINOTECAN	3
BEVACIZUMAB + CAPE + OXALIPLATIN	3
CVD + THALIDOMIDE	3
BEVACIZUMAB + PACLITAXEL	3
IRINOTECAN + RALTITREXED	3
IRINOTECAN + VINCRISTINE	3
CARFILZOMIB + CYCLOPHOSPHAMIDE	3
CLARITY TRIAL	3
BEVACIZUMAB + IRINOTECAN + OXALIPLATIN + MDG	3
MMM	3
LENALIDOMIDE + RITUXIMAB	3
CAPECITABINE + OXALIPLATIN + RT	3
PACLITAXEL + BEVACIZUMAB	3
BEVACIZUMAB + CAPE + IRINOTECAN	3
SHAMASH LBCMVD-56	3
PRIMUS 002 TRIAL	3
CYCLO + RITUXIMAB + VINCRISTINE	3
FLUOROURACIL + IRINOTECAN	3
MERCAPTOPURINE	3
PLATO TRIAL	3
CAPITAL TRIAL	3
BALLAD TRIAL	3
PACLITAXEL + RAMUCIRUMAB	3
DARATUMUMAB + LENALIDOMIDE	3
NEPTUNE TRIAL	3
FLUOROURACIL + OXALIPLATIN + TRASTUZUMAB	3
CAPECITABINE + CISPLATIN + TRASTUZUMAB	3
CYCLOPHOSPHAMIDE + FLUOROURACIL + METHOTREXATE	3
METHOTREXATE + RITUXIMAB	3
PREDNISOLONE + VINBLASTINE + RITUXIMAB	3
COMPLEEMENT-1 TRIAL	3
BEVACIZUMAB + OXALIPLATIN	3
CAPECITABINE + IRINOTECAN + PANITUMUMAB	3
IBRUTINIB + VENETOCLAX	3
CCISV	3
SOPHIA TRIAL	3
BORTEZOMIB + VENETOCLAX	3
TREOSULFAN	3
DEPATUXIZUMAB MAFODOTIN + TEMOZOLOMIDE	3
DARATUMUMAB + POMALIDOMIDE	3
RCD TRIAL	3
OFATUMUMAB	3
CAPECITABINE + CETUXIMAB + OXALIPLATIN	3
CAPECITABINE + OXALIPLATIN + PANITUMUMAB	3
CISPLATIN + TEYSUNO	3
GEMCITABINE + OXALIPLATIN + PACLITAXEL	3
BEVACIZUMAB + TEMOZOLOMIDE	3
BORTEZOMIB + LENALIDOMIDE	3
TEYSUNO	3
ELOTUZUMAB + LENALIDOMIDE	3
BORTEZOMIB + PREDNISOLONE + MELPHALAN	3
ALEMTUZUMAB + CYCLOPHOSPHAMIDE + METHOTREXATE	3
MERCAPTOPURINE + METHOTREXATE + TRETINOIN	3
METHOTREXATE + PROCARBAZINE	3
CNS EPEN2007	3
CNS SIOP 2009	3
IRINOTECAN + MITOMYCIN	3
CYCLOPHOSPHAMIDE + GEMCITABINE + VINCRISTINE	3
LENALIDOMIDE + DARATUMUMAB	3
CAPE + CETUXIMAB + OXALIPLATIN	3
Cisplatin + Irinotecan	3
IXAZOMIB + THALIDOMIDE	3
CARFILZOMIB + POMALIDOMIDE	3
CYCLOPHOSHAMIDE + RITUXIMAB	3
ALEMTUZUMAB + ETOPOSIDE	3
SELINEXOR	3
STREPTOZOCIN	3
MCX	3
BENDAMUSTINE + BORTEZOMIB	3
GEMCITABINE + TEGAFUR	3
PATHOS TRIAL	3
CAPECITABINE + OXALIPLATIN + TRASTUZUMAB	3
CETUXIMAB + IRINOTECAN + RALTITREXED	3
IRINOTECAN + OXALIPLATIN + PACLITAXEL	3
PROMISE-MESO TRIAL	3
SCOPE 1 TRIAL	3
CYCLOPHOSPHAMIDE + RITUXIMAB + VINBLASTINE	3
MERCAPTOPURINE + VINBLASTINE	3
BEVACIZUMAB + IRINOTECAN + TEMOZOLOMIDE	3
CETUXIMAB + OXALIPLATIN	3
ACALABRUTINIB	3
BORTEZOMIB + RITUXIMAB	3
CYCLET	3
IBRUTINIB + RITUXIMAB	3
ABC-08 TRIAL	3
CNS HR MBL Cyclo/Vinc	3
FLUOROURACIL + GEMCITABINE	3
MANTA TRIAL	3
CETUXIMAB + OXALIPLATIN + RALTITREXED	3
VIM TRIAL	3
BEVACIZUMAB + OXALIPLATIN + MDG	3
CYCLOPHOSPHAMIDE + THALIDOMIDE + Dexamethasone	3
IRINOTECAN + OXALIPLATIN + PACLITAXEL	3
IRINOTECAN + PANITUMUMAB + RALTITREXED	3
IRINOTECAN + TALAZOPARIB	3
GELOX	3
PACRITINIB	3
PHITT D3 Vincristine / irinotecan	3
MELPHALAN + THALIDOMIDE	3
MM	3
SHINE TRIAL	3
VE	3
AFLIBERCEPT + CAPECITABINE + IRINOTECAN	3
CLL210 TRIAL	3
CISPLATIN + CYCLOPHOSPHAMIDE + ETOPOSIDE	3
CISPLATIN + RALTITREXED + TRASTUZUMAB	3
AFLIBERCEPT + FLUOROURACIL + IRINOTECAN	3
CISPLATIN + CYCLOPHOSPHAMIDE + VINCRISTINE	3
CYCLOPHOSPHAMIDE + TEMOZOLOMIDE	3
IRINOTECAN + TEMOZOLOMIDE + DINUTUXIMAB BETA	3
CARFILZOMIB + CYCLOPHOSPHAMIDE + LENALIDOMIDE	3
PACIFICO TRIAL	3
CYCLOPHOSPHAMIDE + IXAZOMIB	3
LIPOSOMAL DOXORUBICIN + PACLITAXEL	3
Ofatumumab	3
RITUXIMAB + TEMOZOLOMIDE	3
PACLITAXEL	4
CARBOPLATIN + PACLITAXEL	4
CHOP R	4
DOCETAXEL	4
GEMCARBO	4
CARBOPLATIN + ETOPOSIDE	4
EC	4
UKALL2011	4
DOCETAXEL + PERTUZUMAB + TRASTUZUMAB	4
ABVD	4
FEC + DOCETAXEL	4
BENDAMUSTINE + RITUXIMAB	4
CARBOPLATIN	4
CARBOPLATIN + PEMETREXED	4
DOCETAXEL + FLUOROURACIL + OXALIPLATIN	4
ERIBULIN	4
FEC 100	4
CARBOPLATIN + LIPOSOMAL DOX	4
CARBOPLATIN + DOCETAXEL + PERTUZUMAB + TRASTUZUMAB	4
CARBOPLATIN + VINORELBINE	4
EOX	4
CARBOPLATIN + PEMBROLIZUMAB + PEMETREXED	4
FEC	4
FEC 60 OR 75	4
GEMCITABINE + NAB-PACLITAXEL	4
FEC 100 + DOCETAXEL	4
CYCLOPHOSPHAMIDE + DOCETAXEL	4
CHLORAMBUCIL + OBINUTUZUMAB	4
BEVACIZUMAB + CARBOPLATIN + PACLITAXEL	4
CHOP	4
PACLITAXEL + TRASTUZUMAB	4
CAPECITABINE + EPIRUBICIN + OXALIPLATIN	4
PARTNER TRIAL	4
EC + PACLITAXEL	4
CARBOPLATIN + PACLITAXEL + RT	4
PCV	4
CISPLATIN + VINORELBINE + RT	4
BEVACIZUMAB + CARBO + PACLITAXEL	4
FEC + DOCETAXEL + PERTUZUMAB + TRASTUZUMAB	4
R-EPOCH	4
EPIRUBICIN	4
BENDAMUSTINE + OBINUTUZUMAB	4
STAMPEDE TRIAL	4
CYCLOPHOSPHAMIDE + DOCETAXEL + EPIRUBICIN	4
LIPOSOMAL DOXORUBICIN	4
CAV	4
EC + DOCETAXEL	4
CARBOPLATIN + DOCETAXEL	4
CYCLO + DOXORUBICIN + PACLITAXEL	4
ROSCO TRIAL	4
BEACOPP	4
CAPECITABINE + CARBOPLATIN	4
DOCETAXEL + NINTEDANIB	4
CARBOPLATIN + DOCETAXEL + TRASTUZUMAB	4
CISPLATIN + DOCETAXEL + FLUOROURACIL	4
MELPHALAN	4
CHLORAMBUCIL + RITUXIMAB	4
NAB-PACLITAXEL	4
MYELOMA XI TRIAL	4
DOXORUBICIN + IFOSFAMIDE	4
DTPACE	4
DOXORUBICIN + OLARATUMAB	4
ICON8B TRIAL	4
ICE-R	4
FEC + DOCETAXEL + TRASTUZUMAB	4
ESHAP	4
BRENTUXIMAB	4
ESCALATED BEACOP DAC	4
CHOP + OBINUTUZUMAB	4
TIP	4
DOCETAXEL + GEMCITABINE	4
TRABECTEDIN	4
CYCLOPHOSPHAMIDE + DOCETAXEL + DOXORUBICIN	4
EC + DOCETAXEL + PERTUZUMAB	4
RCEOP	4
ECF	4
DOXORUBICIN	4
VDT-PACE	4
TOPOTECAN	4
CHLVPP	4
BENDAMUSTINE	4
AML MYECHILD	4
CYCLOPHOSPHAMIDE + GEMCITABINE + RITUXIMAB + VINCRISTINE	4
CYCLOPHOSPHAMIDE + DOCETAXEL + TRASTUZUMAB	4
GDP	4
OEPA	4
CARBOPLATIN + CYCLOPHOSPHAMIDE + DOXORUBICIN + PACLITAX	4
ETOPOSIDE + IFOSFAMIDE	4
FEC + TRASTUZUMAB	4
PACLITAXEL + PERTUZUMAB + TRASTUZUMAB	4
GEMCITABINE + OXALIPLATIN + RITUXIMAB	4
DOCETAXEL + TRASTUZUMAB	4
EURO-EWING	4
CHLORAMBUCIL	4
ENRICH TRIAL	4
ATEZOLIZUMAB + BEVACIZUMAB + CARBOPLATIN + PACLITAXEL	4
AC	4
CHOEP	4
CARBOPLATIN + RT	4
CYCLOPHOSPHAMIDE + DOCETAXEL + DOXORUBICIN + PERTUZUMA	4
CYCLOPHOSPHAMIDE + TOPOTECAN	4
VIDE	4
LOMUSTINE	4
ICE	4
CYCLOPHOSPHAMIDE + DOXORUBICIN + PERTUZUMAB + TRASTUZU	4
ESHAP R	4
CYCLOPHOSHAMIDE	4
IVE	4
GEMCITABINE + PACLITAXEL	4
RITUXIMAB + VENETOCLAX	4
ECARBOX	4
RMS 2005 TRIAL	4
CARBOPLATIN + VINORELBINE + RT	4
CYCLOPHOSPHAMIDE + ETOPOSIDE + PROCARBAZINE	4
CLADRIBINE	4
BENDAMUSTINE + CYTARABINE + RITUXIMAB	4
PVD	4
IDELALISIB + RITUXIMAB	4
IFOSFAMIDE	4
SCALOP-2 TRIAL	4
FEC + PACLITAXEL	4
CARBOPLATIN + CYCLOPHOSPHAMIDE + EPIRUBICIN + PACLITAXEL	4
CAPECITABINE + CISPLATIN + EPIRUBICIN	4
VIP	4
MIFAMURTIDE	4
ATEZOLIZUMAB + NAB PACLITAXEL	4
DHAP - R	4
IVA	4
ATEZOLIZUMAB + CARBOPLATIN + ETOPOSIDE	4
CISPLATIN + DOXORUBICIN	4
FEC 100 + TRASTUZUMAB	4
MAP	4
VEPEMB	4
IGEV	4
CARBOPLATIN + CYCLOPHOSPHAMIDE + EPIRUBICIN + PACLITAXEL	4
EP	4
BENDAMUSTINE + THALIDOMIDE	4
BORTEZOMIB + PANOBINOSTAT + THALIDOMIDE	4
FEC 100 + DOCETAXEL + TRASTUZUMAB	4
CARBOPLATIN + VINCRISTINE	4
FCARBOST	4
CYTARABINE + METHOTREXATE + RITUXIMAB + THIOTEPA	4
CAP	4
DACARBAZINE + DOXORUBICIN + VINBLASTINE	4
CARBOPLATIN + CETUXIMAB + FLUOROURACIL	4
CYCLO + DOXORUBICIN + VINCRISTINE	4
LIPOSOMAL DAUNORUBICIN + CYTARABINE (Vyxeos)	4
EMA/CO	4
CYCLO + DOCETAXEL + TRASTUZUMAB	4
PMITCEBO-R	4
CARBOPLATIN + EPIRUBICIN + PACLITAXEL	4
GDP R	4
CISPLATIN + PACLITAXEL	4
CYCLOPHOSPHAMIDE + DOCETAXEL + PERTUZUMAB + TRASTUZUMA	4
CVD R	4
EOF	4
NHL 2003	4
CAPECITABINE + STREPTOZOCIN	4
CYCLOPHOSPHAMIDE + ETOPOSIDE + RITUXIMAB + VINCRISTINE	4
ACTICCA-1 TRIAL	4
DACTINOMYCIN + ETOPOSIDE	4
CVP	4
INTERLACE TRIAL	4
MVAC	4
ICON TRIAL	4
IVE-R	4
PLATFORM TRIAL	4
MUK8 TRIAL	4
CARBO + DOCETAXEL + TRASTUZUMAB	4
DACARBAZINE	4
LOMUSTINE + PROCARBAZINE + VINCRISTINE	4
EDP	4
VAI	4
CAPECITABINE + CARBOPLATIN + EPIRUBICIN	4
DOXORUBICIN EMBOLISATION	4
LUTETIUM-177	4
DHAP	4
BEACOP DAC	4
CARBOPLATIN + CETUXIMAB + FU	4
CHLORAMBUCIL + ETOPOSIDE + LOMUSTINE	4
R-CHOEP	4
METHOTREXATE + VINBLASTINE	4
CNS EPENDYMOMA II	4
COMPARE TRIAL	4
CYCLOPHOSPHAMIDE + DOXORUBICIN	4
POMB	4
CARBOPLATIN + NAB-PACLITAXEL	4
CYCLOPHOSPHAMIDE + DOCETAXEL + DOXORUBICIN + TRASTUZUM	4
CISPLATIN + FU + TRASTUZUMAB	4
ESPAC TRIAL	4
EDP + MITOTANE	4
PROCAID TRIAL	4
TAC	4
CYCLOPHOSPHAMIDE + ETOPOSIDE + LOMUSTINE	4
SCOPE2 TRIAL	4
IVAC	4
ABVD R	4
CISPLATIN + DOX + ETOPOSIDE	4
ACCEPT TRIAL	4
VEDEX	4
IVADO	4
CISPLATIN + ETOPOSIDE + IFOSFAMIDE	4
CEV	4
CISPLATIN + IFOSFAMIDE + PACLITAXEL	4
INTERAACT TRIAL	4
CVP R + GEMCITABINE	4
CYCLOPHOSPHAMIDE + CYTARABINE + MERCAPTOPURINE	4
CYCLOPHOSPHAMIDE + EPIRUBICIN + FLUOROURACIL + PERTUZUM	4
GEMCITABINE + TIP	4
CISPLATIN + LOMUSTINE + VINCRISTINE	4
CAPECITABINE + CARBOPLATIN + TRASTUZUMAB	4
BORTEZOMIB + MELPHALAN	4
LOMUSTINE + PROCARBAZINE	4
GEMCITABINE + IFOSFAMIDE + VINORELBINE	4
CARMUSTINE + THIOTEPA	4
R-GEM-P	4
DACTINOMYCIN + VINCRISTINE	4
SPIRE TRIAL	4
DACARBAZINE + GEMCITABINE	4
CYCLOPHOSPHAMIDE + ETOPOSIDE + RITUXIMAB	4
HD EuroNet-PHL-C2 DECOPDAC	4
ALEMTUZUMAB + CYCLOPHOSPHAMIDE	4
DINUTUXIMAB BETA	4
PHITT TRIAL	4
CISPLATIN + ETOPOSIDE + PACLITAXEL	4
CISPLATIN + TOPOTECAN	4
PIXANTRONE	4
CYCLOPHOSPHAMIDE + VINORELBINE	4
DACTINOMYCIN	4
ETOPOSIDE + IFOSFAMIDE + RITUXIMAB	4
IGEV R	4
EURAMOS TRIAL	4
PAD	4
FOXFIRE TRIAL	4
CAPECITABINE + CARBOPLATIN + RT	4
CHLORAMBUCIL + OFATUMUMAB	4
GEMCITABINE + TREOSULPHAN	4
ROMICAR TRIAL	4
NEO-AEGIS TRIAL	4
STREPTOZOCIN + MDG	4
CARBOPLATIN + TOPOTECAN	4
CAPECITABINE + CISPLATIN + STREPTOZOCIN	4
COPP + DACARBAZINE	4
FEC 60 OR 75 + DOCETAXEL	4
CISPLATIN + EPIRUBICIN + FLUOROURACIL	4
CMV	4
CYTARABINE + OXALIPLATIN + RITUXIMAB	4
CYCLO + ETOPOSIDE + VINCRISTINE	4
HD EURONET-PHL	4
FCIST	4
MUK7 TRIAL	4
BENDAMUSTINE + GEMCITABINE + VINORELBINE	4
RATHL TRIAL	4
METHOTREXATE + VINORELBINE	4
R-CHOP / R-DHAP	4
HEPATOBLASTOMA	4
CARBO + LIPOSOMAL DOX + PACLITAXEL	4
CLADRIBINE + RITUXIMAB	4
BLEOMYCIN	4
VDC	4
CHLORAMBUCIL + DEXAMETHASONE + ETOPOSIDE	4
CISPLATIN + EPIRUBICIN	4
EC + PACLITAXEL + PERTUZUMAB	4
EP/EMA	4
CARBOPLATIN + DOCETAXEL + FLUOROURACIL	4
GEMCITABINE + VINORELBINE	4
MUK 12 TRIAL	4
BENDAMUSTINE + BRENTUXIMAB	4
DACARBAZINE + DOXORUBICIN + VINCRISTINE	4
CYCLOPHOSPHAMIDE + DOXORUBICIN + RITUXIMAB	4
CYCLOPHOSPHAMIDE + DOXORUBICIN + ETOPOSIDE + VINCRISTINE	4
ECARBOF	4
CHLORAMBUCIL + LOMUSTINE	4
EPIRUBICIN + FLUOROURACIL + OXALIPLATIN	4
CYCLO + ETOPOSIDE + NELARABINE	4
PEI	4
WILMS REL ICE	4
CISPLATIN + IFOSFAMIDE + MESNA + VINBLASTINE	4
CARBOPLATIN + EPIRUBICIN + FLUOROURACIL	4
CYCLOPHOSPHAMIDE + ETOPOSIDE + PROCARBAZINE + RITUXIMAB	4
BORTEZOMIB + CISPLATIN + CYCLOPHOSPHAMIDE + DOXORUBICIN -	4
PENTOSTATIN + RITUXIMAB	4
CISPLATIN + FLUOROURACIL + STREPTOZOCIN	4
BEVACIZUMAB + CISPLATIN + PACLITAXEL	4
CHLVPP/EVA	4
CARBOPLATIN + DOXORUBICIN	4
PMITCEBO	4
HD EuroNet-PHL-C2 COPDAC	4
CHOP + INTERFERON ALFA + ZIDOVUDINE	4
CISPLATIN + DOCETAXEL	4
GEMCITABINE + RITUXIMAB	4
CVD (NEUROENDOCRINE)	4
CYTARABINE + ETOPOSIDE + TRETINOIN	4
DACARBAZINE + DOXORUBICIN	4
BLEOMYCIN + CISPLATIN + VINCRISTINE	4
FEC + PACLITAXEL + TRASTUZUMAB	4
PROCARBAZINE	4
FLUDARABINE	4
BORTEZOMIB + DOXORUBICIN	4
BLEOMYCIN + CISPLATIN + VINBLASTINE	4
CYCLOPHOSPHAMIDE + DACARBAZINE + VINCRISTINE	4
ETOPOSIDE + RITUXIMAB	4
HD EuroNet-PHL-C2 OEPA	4
CARFILZOMIB + LENALIDOMIDE	4
CISPLATIN + LIPOSOMAL DOXORUBICIN	4
EPSSG	4
CARBOPLATIN + PACLITAXEL + TRASTUZUMAB	4
SELPAC TRIAL	4
CHLORAMBUCIL + VINBLASTINE	4
CARBO + ETOPOSIDE + VINCRISTINE	4
NEOBLADE TRIAL	4
BOP	4
CISPLATIN + DACARBAZINE + VINBLASTINE	4
CYCLOPHOSPHAMIDE + DACTINOMYCIN + VINCRISTINE	4
ETOPOSIDE + LOMUSTINE + VINCRISTINE	4
CYCLO + FLUDARABINE + RITUXIMAB	4
INCA TRIAL	4
METAPHER TRIAL	4
CARBOPLATIN + EPIRUBICIN	4
CARBO + GEMCITABINE + RITUXIMAB	4
CAPE + CARBOPLATIN + CETUXIMAB	4
CARBO + THIOTEPA + TOPOTECAN	4
CARBOPLATIN + FLUOROURACIL + STREPTOZOCIN	4
BEVACIZUMAB + CARBO + GEMCITABINE	4
CARBOPLATIN + ETOPOSIDE + IFOSFAMIDE	4
CISPLATIN + DOXORUBICIN + ETOPOSIDE	4
CARBOPLATIN + CYCLOPHOSPHAMIDE + ETOPOSIDE	4
DACTINOMYCIN + ETOPOSIDE + METHOTREXATE	4
DACTINOMYCIN + CYCLO + VINCRISTINE	4
MUK6 TRIAL	4
POUT TRIAL	4
Bortezomib + DA-EPOCH-R	4
CISPLATIN + CYCLO + DOXORUBICIN	4
DA-EPOCH + BORTEZOMIB	4
RPACE-BOM	4
CYTARABINE + ETOPOSIDE	4
CAPECITABINE + DOCETAXEL	4
EPIRUBICIN + OXALIPLATIN	4
CHOP R + BORTEZOMIB	4
CYCLOPHOSPHAMIDE + CYTARABINE + MERCAPTOPURINE + METHO	4
CARBOPLATIN + NAB-PACLITAXEL + TRASTUZUMAB	4
EC + DOCETAXEL + TRASTUZUMAB	4
IFOSFAMIDE + PACLITAXEL	4
NAB-PACLITAXEL + TRASTUZUMAB	4
AC + DOCETAXEL + TRASTUZUMAB	4
GEMCITABINE + RITUXIMAB + VINORELBINE	4
CARBOPLATIN + EPIRUBICIN + ETOPOSIDE + MELPHALAN	4
DACTINOMYCIN + DOXORUBICIN + VINCRISTINE	4
LOMUSTINE + TEMOZOLOMIDE	4
CARBOPLATIN + ETOPOSIDE + VINCRISTINE	4
CYCLOPHOSPHAMIDE + PACLITAXEL	4
mEOC TRIAL	4
CHOP-R + CYTARABINE	4
CISPLATIN + DOXORUBICIN + METHOTREXATE	4
CISPLATIN + DOX + ETOPOSIDE + MITOTANE	4
OPTIMAL TRIAL	4
CARBOPLATIN + IFOSFAMIDE	4
CYCLOPHOSPHAMIDE + DOXORUBICIN + ETOPOSIDE	4
CYCLOPHOSPHAMIDE + FLUOROURACIL + METHOTREXATE + Trastuz	4
PETREA TRIAL	4
G-CVP	4
CISPLATIN + DACTINOMYCIN + ETOPOSIDE + METHOTREXATE	4
CARBOPLATIN + TRASTUZUMAB	4
CVAD	4
CYCLOPHOSPHAMIDE + DOXORUBICIN + METHOTREXATE + VINCRIS	4
CYCLOPHOSPHAMIDE + FLUDARABINE + THIOTEPA	4
LOPP	4
BUSULPHAN + CYCLO	4
CHLORAMBUCIL + DOXORUBICIN + ETOPOSIDE + PROCARBAZINE +	4
ETOPOSIDE + IFOSFAMIDE + RITUXIMAB + THIOTEPA	4
TIDE	4
MIC	4
PANTHER TRIAL	4
MELPHALAN + TOPOTECAN	4
CARBOPLATIN + ETOPOSIDE + PACLITAXEL	4
CYCLOPHOSPHAMIDE + DOXORUBICIN + PROCARBAZINE + VINCRIST	4
MITOXANTRONE + TRETINOIN	4
RETINOBLASTOMA	4
WT Relapse Carbo/Cyclo/Etop	4
NEMO TRIAL	4
BEVACIZUMAB + LOMUSTINE	4
CISPLATIN + NAB-PACLITAXEL	4
CARBOPLATIN + RALTITREXED	4
QUASAR 2 TRIAL	4
CISPLATIN + GEMCITABINE + PACLITAXEL	4
CYCLOPHOSPHAMIDE + METHOTREXATE	4
Z-DEX	4
PACKER INDUCTION VINCRISTINE + RT	4
BEVACIZUMAB + CARBOPLATIN + NAB-PACLITAXEL	4
DOXORUBICIN + GEMCITABINE	4
OCTOPUS TRIAL	4
ARISTOTLE TRIAL	4
CARBOPLATIN + EPIRUBICIN + METHOTREXATE + VINBLASTINE	4
CARBOPLATIN + FLUOROURACIL + TRASTUZUMAB	4
CISPLATIN + CYCLOPHOSPHAMIDE + DOXORUBICIN	4
DOXORUBICIN + STEPTOZOTOCIN	4
CYCLOPHOSPHAMIDE + ETOPOSIDE + LOMUSTINE + RITUXIMAB	4
RIALTO TRIAL	4
STS Non- Rhabdo EpSSG Vinblastine/MTX	4
ICE TRIAL	4
MVCARBO	4
CABAZITAXEL + CARBOPLATIN	4
CHOP + BORTEZOMIB	4
GDCVP	4
EPIRUBICIN + FLUOROURACIL	4
IDARUBICIN + MERCAPTOPURINE + METHOTREXATE + VINCRISTINE	4
PMITCEBO R	4
CYCLOPHOSPHAMIDE + DOXORUBICIN + PACLITAXEL	4
ARIEL 4 TRIAL	4
CISPLATIN + DACARBAZINE	4
BLEOMYCIN + OXALIPLATIN + VINCRISTINE	4
CNS Germ Cell Tumour II - Non Germinomatous - Standard PEI	4
ETOPOSIDE + IFOSFAMIDE + METHOTREXATE	4
METHOTREXATE + PROCARBAZINE + RITUXIMAB	4
CARBOPLATIN + DACARBAZINE	4
CNS Medbl St Risk <3yrs induction	4
CYCLOPHOSPHAMIDE + IDARUBICIN	4
ReBEL TRIAL	4
ACE	4
BLEOMYCIN + VINCRISTINE	4
CHLVPP/PABLOE	4
CYTARABINE + OXALIPLATIN	4
PHITT D Carboplatin/Doxorubicin	4
BLEOMYCIN + CYCLOPHOSPHAMIDE + ETOPOSIDE + GEMCITABINE +	4
BLEOMYCIN + CYCLOPHOSPHAMIDE + ETOPOSIDE + METHOTREXAT	4
DOXORUBICIN + PEGASPARGASE + VINCRISTINE	4
PAC-E	4
SIOP	4
BENDAMUSTINE + GEMCITABINE + RITUXIMAB + VINORELBINE	4
CYCLOPHOSPHAMIDE + CYTARABINE + POMALIDOMIDE	4
PHITT C CDDP-M	4
ADOC	4
CARBOPLATIN + DOXORUBICIN + MITOMYCIN	4
CARBOPLATIN + ETOPOSIDE + METHOTREXATE	4
CARBOPLATIN + THIOTEPA + TOPOTECAN	4
CYCLOPHOSPHAMIDE + VINBLASTINE	4
HERBY TRIAL	4
HLH Induction W3-8 Etoposide/Dexamethasone/Ciclosporin	4
CARBOPLATIN + ETOPOSIDE + THIOTEPA	4
ERIBULIN + TRASTUZUMAB	4
ALEMTUZUMAB + ETOPOSIDE + METHOTREXATE	4
PHITT D2 Carboplatin / etoposide	4
VIOLA TRIAL	4
BEVACIZUMAB + CARBO + DOCETAXEL	4
CISPLATIN + DOXORUBICIN + IFOSFAMIDE	4
BORTEZOMIB + POMALIDOMIDE	4
EW Rel rEECur Ifosfamide	4
ALEMTUZUMAB + FLUDARABINE + THIOTEPA + TREOSULFAN	4
ALEMTUZUMAB + FLUDARABINE + TREOSULFAN	4
BLEOMYCIN + CISPLATIN + ETOPOSIDE + PACLITAXEL	4
CARBOPLATIN + CYCLO + DOXORUBICIN	4
CNS Ependymoma 99 vinc+cyclo+etop	4
CNS Medbl HR	4
CYCLOPHOSPHAMIDE + MITOXANTRONE + RITUXIMAB + VINCRISTINI	4
EPIRUBICIN + OXALIPLATIN + RALTITREXED	4
FLUOROURACIL + LOMUSTINE	4
PHITT C Carboplatin/Doxorubicin	4
PHITT E2 PLADO	4
WT2001	4
ASPARAGINASE + CYTARABINE + ETOPOSIDE + METHOTREXATE	4
BLEOMYCIN + CYCLOPHOSPHAMIDE + GEMCITABINE + RITUXIMAB +	4
CLADRIBINE + CYCLOPHOSPHAMIDE + RITUXIMAB	4
ESTRAMUSTINE	4
PACE-BOM	4
CARMUSTINE + CYCLOPHOSPHAMIDE + ETOPOSIDE	4
CHLORAMBUCIL + ETOPOSIDE + RITUXIMAB	4
CISPLATIN + CYCLOPHOSPHAMIDE	4
CYTARABINE + IDARUBICIN + METHOTREXATE + RITUXIMAB	4
DACTINOMYCIN + IFOSFAMIDE + VINCRISTINE	4
FMD	4
MAP + MIFAMURTIDE	4
MUK 11 TRIAL	4
CLOFARABINE + CYCLOPHOSPHAMIDE + ETOPOSIDE	4
CLOFARABINE	4
CYCLOPHOSPHAMIDE + DOXORUBICIN + METHOTREXATE + RITUXIM	4
ROVALPITUZUMAB TESIRINE	4
ALEMTUZUMAB + FLUDARABINE	4
CLADRIBINE + CYTARABINE + IDARUBICIN	4
HLH Consol Etop/Dexamthasone/Ciclosporin	4
CARBOPLATIN + PROCARBAZINE	4
CYTARABINE + FLUDARABINE + RITUXIMAB	4
CYTARABINE + IDARUBICIN	4
CYTARABINE + MERCAPTOPURINE + METHOTREXATE + PEGASPARA	4
HELIOS TRIAL	4
IBRITUMOMAB TIUXETAN + RITUXIMAB + Y90	4
Idarubicin + Tretinoin	4
CYCLOPHOSPHAMIDE + DOCETAXEL + EPIRUBICIN + TRASTUZUMAB	4
DOXORUBICIN + METHOTREXATE + MIFAMURTIDE	4
IDARAM	4
BLEOMYCIN + CYCLOPHOSPHAMIDE + ETOPOSIDE + GEMCITABINE +	4
CARBOPLATIN + TEYSUNO	4
CISPLATIN + DOXORUBICIN + METHOTREXATE + MIFAMURTIDE	4
CVP R + ETOPOSIDE	4
STS Non-Rhabdo EpSSG 2005 Ifos	4
STS Non-Rhabdo EpSSG 2005 VA	4
CARBOPLATIN + CYCLOPHOSPHAMIDE + EPIRUBICIN + FLUOROURA	4
CCEP	4
CYCLOPHOSPHAMIDE + NAB-PACLITAXEL	4
DENOSUMAB + ERIBULIN	4
IFOSFAMIDE + VINCRISTINE	4
CISPLATIN + CYTARABINE	4
CYCLOPHOSPHAMIDE + DACTINOMYCIN	4
CYCLOPHOSPHAMIDE + ETOPOSIDE + THALIDOMIDE	4
MEAD (PEG-ASPARAGINASE)	4
R-PCE2BO (ETOPOPHOSï¿½)	4
BEVACIZUMAB + CARBO	4
CARBOPLATIN + CETUXIMAB	4
CARMUSTINE	4
GEMCITABINE + OXALIPLATIN + PEGASPARAGINASE	4
METHOTREXATE + MITOMYCIN + MITOXANTRONE	4
AZACITIDINE	5
BEP	5
UKALL2014	5
CYTARABINE	5
TRIFLURIDINE + TIPIRACIL	5
CYCLOPHOSPHAMIDE	5
DA	5
FLAG + IDARUBICIN	5
FCR	5
AML19 TRIAL	5
FLAIR TRIAL	5
R IVAC	5
BEAM	5
MATRIX TRIAL	5
ARSENIC TRIOXIDE + TRETINOIN	5
ALEMTUZUMAB + FLUDARABINE + MELPHALAN	5
AML18 TRIAL	5
R CODOX-M/ R IVAC	5
R CODOX M	5
CYTARABINE + DAUNORUBICIN + GEMTUZUMAB OZOGAMICIN	5
MYELOMA XII TRIAL	5
RADIUM 223	5
LEAM	5
METHOTREXATE HIGH DOSE	5
ALEMTUZUMAB	5
CHOP R + METHOTREXATE	5
CYTARABINE HIGH DOSE	5
CYCLOPHOSPHAMIDE + FLUDARABINE	5
UKALL60+	5
ATG + BUSULFAN + FLUDARABINE	5
CYCLOPHOSPHAMIDE HIGH DOSE	5
NORDIC	5
AML17 TRIAL	5
UKALLR3	5
FBC	5
ALL INTERFANT 06	5
ALL INTERIM GUIDELINES	5
Yescarta + Fludarabine + Cyclophosphamide	5
CARBOPLATIN + ETOPOSIDE + IFOSFAMIDE + RITUXIMAB	5
STS RHABDO RMS 2005	5
ALEMTUZUMAB + CYCLO + FLUDARABINE	5
EW REL REECUR	5
STS RHABDO VIT0910	5
ARSENIC TRIOXIDE	5
APML	5
UKALL2003	5
ATG + CYCLOPHOSPHAMIDE TBI ALLO	5
ARSENIC TRIOXIDE + ATRA	5
ALL INTENSIFICATION	5
FLAG	5
POMB/ACE	5
CYTARABINE + RITUXIMAB	5
INOTUZUMAB OZOGAMICIN	5
AML16 TRIAL	5
AML 18 PILOT TRIAL	5
SMILE	5
BLEOMYCIN + CARBOPLATIN + ETOPOSIDE	5
MIDOSTAURIN	5
ALL PH+VE	5
ALEMTUZUMAB + BUSULFAN + CYCLOPHOSPHAMIDE	5
BLEOMYCIN + CARBOPLATIN + CISPLATIN + VINCRISTINE	5
LACE	5
CODOX M	5
CYTARABINE HD + METHOTREXATE HD	5
FAB LMB	5
ALL Interim Guide 2019 Induction A	5
LI-1 TRIAL	5
IDARUBICIN	5
AMSACRINE + ATG+ BUSULFAN + CYTARABINE + FLUDARABINE	5
AMSACRINE + ATG + BUSULFAN + CYTARABINE + FLUDARABINE	5
Kymriah + Fludarabine + Cyclophosphamide	5
DOXORUBICIN + HD MTX	5
BEAM + ALEMTUZUMAB	5
DA-EPOCH	5
DAUNORUBICIN + CYTARABINE + MIDOSTAURIN	5
ALL Interim Guide 2019 Augmented BFM Consolidation C	5
FLUDARABINE + MELPHALAN	5
ALEMTUZUMAB + PENTOSTATIN	5
IFOSFAMIDE + LIPOSOMAL DOXORUBICIN	5
ATG	5
CHOP-R + CYTARABINE HIGH DOSE	5
ALL MAINTENANCE	5
CYCLOPHOSPHAMIDE + CYTARABINE + DOXORUBICIN + RITUXIMAB -	5
CISPLATIN + DACTINOMYCIN + ETOPOSIDE + FILGRASTIM + METHOT	5
ALEMTUZUMAB + CYCLOPHOSPHAMIDE + FLUDARABINE	5
AML DOWNS	5
ALL Interim Guide 2019 Delayed Intensification A	5
ACE (GERM CELL)	5
IDARUBICIN + TRETINOIN	5
ALL Interim Guide 2019 Maintenance	5
ALL Interim Guide 2019 Standard BFM Consolidation B	5
UKALL 60+ TRIAL	5
ALL Interim Guide 2019 Standard Interim Maintenance A	5
ALL Interim Guide 2019 Induction B	5
DAUNORUBICIN + PEGASPARAGINASE + VINCRISTINE	5
AML INTERIM GUIDELINES	5
CYTARABINE + METHOTREXATE + RITUXIMAB	5
ALL INDUCTION	5
FIGARO TRIAL	5
CYTARABINE + ETOPOSIDE + IFOSFAMIDE + METHOTREXATE + RITU:	5
TRANSPLANT BEAM	5
GEMTUZUMAB OZOGAMICIN	5
DAUNORUBICIN + VNCRISTINE	5
TRANSPLANT CYTARABINE + ETOPOSIDE + LOMUSTINE + MELPHALA	5
MACE	5
TRANSPLANT ALEMTUZUMAB + FLUDARABINE + MELPHALAN	5
NELARABINE	5
UKALL XII	5
ALL R T-CELL CONSOLIDATION	5
METHOTREXATE + RITUXIMAB + TEMOZOLOMIDE	5
ADE	5
TRANSPLANT CYCLOPHOSPHAMIDE + FLUDARABINE (+/- TBI)	5
CARBOPLATIN + CYCLOPHOSPHAMIDE + ETOPOSIDE + PACLITAXEL	5
ALL CONSOLIDATION	5
ETOPOSIDE + IFOSFAMIDE + MTX	5
ROMAZA TRIAL	5
ALL Int Guide B BFMCons	5
CLOFARABINE + CYTARABINE	5
CYTARABINE IT + METHOTREXATE	5
FLA	5
ALL Int Guide A Ind	5
CYTARABINE + METHOTREXATE	5
ALL Interim Guide 2019	5
IDARUBICIN + MITOXANTRONE + TRETINOIN	5
CYTARABINE + FLUDARABINE	5
AZACITIDINE + VENETOCLAX	5
ALL Int GdeC D Int PhIV reind PAsp	5
BENDAMUSTINE + CYTARABINE + ETOPOSIDE + MELPHALAN	5
CYTARABINE + ETOPOSIDE + MITOXANTRONE	5
BUSULFAN + FLUDARABINE	5
CODOX M + IVAC	5
ALEMTUZUMAB + LEAM	5
BUSULFAN + CYCLOPHOSPHAMIDE	5
CYTARABINE + MIDOSTAURIN	5
ERWINIA ASPARAGINASE	5
TRANSPLANT ALEMTUZUMAB + CYTARABINE + ETOPOSIDE + LOMUS	5
111 TRIAL	5
AMSACRINE + BUSULFAN + CYTARABINE + FLUDARABINE	5
ALL Interim Guide 2019 Consolidation A	5
FLUDARABINE + THIOTEPA + TREOSULFAN	5
AML Relapsed FLA - Idarubicin	5
TRIFLURIDINE TIPIRACIL	5
AZACITIDINE + RUXOLITINIB	5
BLEOMYCIN + CISPLATIN + ETOPOSIDE + METHOTREXATE + VINCRIS	5
ATG + BUSULFAN + FLUDARABINE + THIOTEPA	5
IMATINIB + MERCAPTOPURINE + VINCRISTINE	5
ATG + CYCLOPHOSPHAMIDE + FLUDARABINE	5
BUSULFAN + MELPHALAN	5
CANTATA TRIAL	5
BLEOMYCIN + CISPLATIN + ETOPOSIDE	5
ATG + CYCLOPHOSPHAMIDE	5
BLEOMYCIN + CYCLOPHOSPHAMIDE + ETOPOSIDE + GEMCITABINE +	5
CNS ATRT Doxorubicin/Ifosfamide/Carboplatin/Etoposide/Methotrexate	5
CYCLOPHOSPHAMIDE + CYTARABINE + DAUNORUBICIN + MERCAPTC	5
DOXORUBICIN + METHOTREXATE	5
ATG + FLUDARABINE	5
CAPECITABINE + LOMUSTINE	5
METHOTREXATE HIGH DOSE + RITUXIMAB	5
ALL Interim Guide 2019 Standard Interim Maintenance B	5
METHOTREXATE HD + VINCRISTINE	5
GILTERITINIB	5
BUSULFAN + CYCLOPHOSPHAMIDE + FLUDARABINE + THIOTEPA	5
CYTARABINE + Gemtuzumab Ozogamicin	5
MA	5
BEAM + FLUDARABINE	5
AMSACRINE + CYTARABINE	5
CYTARABINE + FLUDARABINE + IDARUBICIN	5
DAUNORUBICIN	5
TRANSPLANT ATG + FLUDARABINE + THIOTEPA + TREOSULFAN	5
GCLAC	5
BUSULFAN + FLUDARABINE + METHOTREXATE	5
IMATINIB + MERCAPTOPURINE + METHOTREXATE	5
ALL Int Guide B Int Maint	5
TRANSPLANT ALEMTUZUMAB + CYCLOPHOSPHAMIDE + FLUDARABIN	5
DOXORUBICIN + IFOSFAMIDE + VINCRISTINE	5
BUSULFAN + THIOTEPA	5
CLOFARABINE + CYTSARABINE + IDARUBICIN	5
ALL NOPHO	5
AMSACRINE + ATG + CYCLOPHOSPHAMIDE + CYTARABINE + FLUDAR	5
CISPLATIN + ETOPOSIDE + METHOTREXATE	5
ALL Interfant 06 ADE	5
ALEMTUZUMAB + FLUDARABINE + LEAM	5
ETOPOSIDE + MELPHALAN	5
IDARUBICIN + IMATINIB + VINCRISTINE	5
ALL Interim Guide 2019 Induction C	5
TRANSPLANT BUSULFAN + FLUDARABINE	5
ALL Interim Guide 2019 NOPHO C	5
CYCLOPHOSPHAMIDE + CYTARABINE + ETOPOSIDE + METHOTREXA'	5
CLADRIBINE + CYTARABINE + DAUNORUBICIN	5
TRANSPLANT AMSACRINE + ANTITHYMOCYTE IMMUNOGLOBULIN (R/	5
TRANSPLANT ATG + BUSULFAN + FLUDARABINE + THIOTEPA	5
CLADRIBINE + CYTARABINE	5
DE ANGELIS	5
FORODESINE	5
ATG + FLUDARABINE + MELPHALAN	5
ATG + FLUDARABINE + THIOTEPA + TREOSULFAN	5
TRANSPLANT FLUDARABINE + THIOTEPA + TREOSULFAN	5
CARBOPLATIN + CYTARABINE + ETOPOSIDE	5
CYCLOPHOSPHAMIDE + ETOPOSIDE + NELARABINE	5
DECITABINE	5
MIDAC	5
BLEOMYCIN + CYCLOPHOSPHAMIDE + ETOPOSIDE + GEMCITABINE +	5
BLEOMYCIN + CYCLOPHOSPHAMIDE + ETOPOSIDE + GEMCITABINE +	5
CYTARABINE + VINCRISTINE	5
IDARUBICIN + METHOTREXATE + VINCRISTINE	5
MTX HD + PROCARBAZINE + VINCRISTINE	5
BEAM R	5
BUSULFAN + CYCLOPHOSPHAMIDE + FLUDARABINE	5
CARBOPLATIN + CYTARABINE + RITUXIMAB	5
CYTARABINE + ETOPOSIDE + IMATINIB	5
CYTARABINE + ETOPOSIDE + METHOTREXATE	5
ALL/LBL T Cell Relapse Intensification	5
BLEOMYCIN + CYCLOPHOSPHAMIDE + ETOPOSIDE + MITOXANTRONI	5
CARBOPLATIN + ETOPOSIDE + IFOSFAMIDE + PACLITAXEL	5
CISPLATIN + CYCLOPHOSPHAMIDE + CYTARABINE + DOXORUBICIN +	5
DAUNORUBICIN + METHOTREXATE + PEGASPARAGINASE + VINCRIS'	5
CNS HGG CISPLATIN/ETOPOSIDE	5
CYTARABINE + METHOTREXATE HD	5
ALL UKALL2011 TRIAL + ALL UKALL2011 Intensfication MTX	5
TRANSPLANT ATG + ETOPOSIDE (+/- TBI)	5
ALL/LBL T Cell Relapse Consolidation	5
YTTRIUM-90	5
ALL INTENSIFICATION MTX	5
AML RELAPSED	5
CAMELLIA TRIAL	5
SPANISH MAINTENANCE	5
ALL/LBL T Cell Relapse NECTAR Cyclophosphamide, etoposide, nelarabine and IT	5
HORMONES	1
CYTARABINE + METHOTREXATE + PEGASPARAGINASE	5
CHOP R + LIPOSOMAL DOXORUBICIN	3
CYCLOPHOSPHAMIDE + DACTINOMYCIN + DOXORUBICIN + VINCRISTINE	5
TRIAL	6
CYCLOPHOSPHAMIDE + FLUDARABINE + MITOXANTRONE	3
CYCLOPHOSPHAMIDE + LIPOSOMAL DOX	3
VELIPARIB	1
CARBOPLATIN + IRINOTECAN	3

For peer review only

For peer review only - http://bmjopen.bmj.com/site/about/guidelines.xhtml

BMJ Open

A common protocol for validation of the QCOVID algorithm across the four UK nations

Journal:	*BMJ Open*
Manuscript ID	bmjopen-2021-050994.R2
Article Type:	Protocol
Date Submitted by the Author:	14-May-2022
Complete List of Authors:	Kerr, Steven; The University of Edinburgh Usher Institute of Population Health Sciences and Informatics,<br>Robertson, Chris; University of Strathclyde, Department of Mathematics and Statistics<br>Nafilyan, Vahe; Office for National Statistics<br>Lyons, Ronan; University of Wales Swansea, Swansea Clinical School<br>Kee, Frank; Queen's University Belfast, UKCRC Centre of Excellence for Public Health (NI)<br>Cardwell, Christopher; Queen's University Belfast, School of Medicine, Dentistry and Biomedical Sciences<br>Coupland, Carol; University of Nottingham, Division of Primary Care<br>Lyons, Jane; Swansea University Medical School<br>Humberstone, Ben; Office for National Statistics<br>Hippisley-Cox, Julia; University of Oxford, Nuffield Department of Primary Care Sciences<br>Sheikh, Aziz; University of Edinburgh, Division of Community Health Sciences
**Primary Subject Heading**:	Epidemiology
Secondary Subject Heading:	Infectious diseases, Respiratory medicine
Keywords:	COVID-19, Epidemiology < INFECTIOUS DISEASES, Public health < INFECTIOUS DISEASES

SCHOLARONE™ Manuscripts

A common protocol for validation of the QCOVID algorithm across the four UK nations

Steven Kerr, stevenkerr2@gmail.com (corresponding author) [1]

Chris Robertson [2,3]

Vahe Nafilyan [4]

Ronan A Lyons [5]

Frank Kee [6]

Chris Cardwell [6]

Carol Coupland [7]

Jane Lyons [5]

Ben Humberstone [4]

Julie Hippisley-Cox [8]

Aziz Sheikh [1]

[1] Usher Institute, The University of Edinburgh, NINE Edinburgh BioQuarter, Edinburgh, UK, EH16 4UX.

[2] University of Strathclyde, Glasgow, UK.

[3] Public Health Scotland, UK

[4] Office for National Statistics, UK.

[5] Population Data Science, Swansea University Medical School, Swansea, UK.

[6] Queen's University Belfast, Belfast, UK.

[7] University of Nottingham, Nottingham, UK.

[8] University of Oxford, Oxford, UK.

Keywords: Covid-19, Coronavirus, QCOVID, Epidemiology, Public Health.

Word count: 1,653

For peer review only

ABSTRACT

Introduction:

The QCOVID algorithm is a risk prediction tool for infection and subsequent hospitalisation/death due to SARS-Cov-2. At the time of writing, it is being used in important policymaking decisions by the UK and devolved governments for combatting the Covid-19 pandemic, including deliberations on shielding and vaccine prioritisation. There are four statistical validations exercises currently planned for the QCOVID algorithm, using data pertaining to England, Northern Ireland, Scotland and Wales respectively. This paper presents a common procedure for conducting and reporting on validation exercises for the QCOVID algorithm.

Methods and Analysis:

We will use open, retrospective cohort studies to assess the performance of the QCOVID risk prediction tool in each of the four UK nations. Linked datasets comprising of primary and secondary care records, virological testing data and death registrations will be assembled in trusted research environments in England, Scotland, Northern Ireland and Wales. We will seek to have population level coverage as far as possible within each nation. The following performance metrics will be calculated by strata: Harrell's C, Brier score, $R^{2,}$ and Royston's D.

Ethics and dissemination:

Approvals have been obtained from relevant ethics bodies in each UK nation. Findings will be made available to national policymakers, presented at conferences and published in peer reviewed journal

Strengths and limitations of this study:

- We will use national level data within each UK nation
- There are potential issues with missing data and differences in the way data is recorded in each country.
- We will evaluate the performance of the algorithm according to several relevant metrics.

For peer review only

INTRODUCTION:

The QCOVID algorithm [1] has been developed to help identify adults at high risk of being hospitalised or dying following infection with SARS-Cov-2 (Severe acute respiratory syndrome coronavirus 2). The algorithm takes as input a total of 40 variables including age, sex, ethnicity, Townsend deprivation score [2] and housing category, as well as clinical information including body mass index (BMI) and 33 variables related to medical conditions and treatments. It outputs the predicted probability that an individual will be infected with SARS-Cov-2 and then hospitalised, and the predicted probability that an individual will be infected with SARS-Cov-2 and then die, over a 90 day period. The algorithm was trained using information from the QResearch database [3], which as of April 2020 contained routinely collected data from 1205 General Practices across England, covering 10.5 million patients. The initial training dataset comprised of a cohort of 6.08 million individuals tracked from the 24 January 2020 to 30 April 2020, and was validated on a subset of 2.17 million individuals tracked from 1 May 2020 to 30 June 2020. The research protocol for the development of the QCOVID algorithm can be found in [4].

The QCOVID algorithm was commissioned by the Chief Medical Officer for England on behalf of the UK government. The algorithm has been used to inform UK and devolved government policy on combatting the SARS-Cov-2 pandemic, including guidance on social-distancing and shielding measures, as well vaccine prioritisation. [5] It is therefore of great importance to validate the predictions of the algorithm in sub-populations of the UK that were not in the initial training set, but will potentially be subject to those policies.

At the time of writing, there are validation exercises planned in Scotland, Northern Ireland and Wales, and a validation exercise underway in England. Validation work was considered urgent and has been expedited in order to support national decision making. In order to facilitate useful comparison of the results of the separate validation exercises, it is necessary to establish a consistent set of procedures. The purpose of this paper is to explicate a common methodology for the validation of the QCOVID algorithm across the four nations of the UK.

METHODS AND ANALYSIS:

Study design:

For peer review only

Open, retrospective cohort study designs will be employed, making use of routinely collected data from General Practices for clinical and demographic information, as well as linked datasets on hospital admissions, reverse-transcription polymerase chain reaction (RT-PCR) testing for Covid-19, and registered deaths. We will aim to have national coverage as far as is possible within each of the four nations of the UK.

Data Sources:

Box 1 contains a brief summary of the main datasets that will be used in the validation exercise for each nation

Box 1: Main datasets to be used

England: Office for National Statistics (ONS) Public Health Linked Data Asset. This dataset is based on the 2011 Census in England covering 40.1 million people, linked at individual level using the NHS number to mortality records, Hospital Episode Statistics (HES) and the General Practice Extraction Service (GPES) data for pandemic planning and research. The data covers 80% of the population of England aged 19 and over.

Northern Ireland: National Health Application and Infrastructure Services (NHAIS) will be used for demographic information. The Patient Administration System (PAS) will be used for data on hospital admissions. Death data will be drawn from the Registrar General, and identified as Covid-19 related through the official Northern Ireland Statistics and Research Agency (NISRA) dashboard. The General Practice Information Platform (GPIP) will bring together GP records from practices across Northern Ireland into a single dataset for use in the validation. As this is not held in the Honest Broker Service, a separate request to its governance board is being made. The Electronic Prescribing Database (EPD) will be used to access information on prescriptions.

Scotland: EAVE II dataset [6]. Contains primary health care records for 5.4 million people covering 99% of the population of Scotland, linked with secondary care data from Scottish Morbidity Record (SMR), Covid-19 test results from Electronic Communication of Surveilance Scotland (ECOSS), and mortality data from National Records Scotland.

Wales: Secure Anonymised Information Linkage (SAIL system) [7]. This will utilise the Controlling Covid (ConCOV) platform linking records on 3.2 million people from the NHS population spine with hospital (Patient Episode Database for Wales), Welsh Longitudinal GP record (WLGP), Covid-19 test results from the Laboratory Information Management System (LIMS), and mortality and 2011Census data from the Office for National Statistics (ONS) [8]

For peer review only

Selection criteria:

Any individual in the relevant linked dataset between the ages of 19 and 100 will be included. Individuals who had an event (hospitalisation or death) in the first period (24 January 2020 – 30 April 2020) will be excluded from any analysis in the second period (1 May 2020 – 30 June 2020). These time periods were chosen to mirror the time periods in the original QCOVID paper. After the vaccination programme started in the UK on 8 December 2020, work had already begun on QCOVID 2&3, which will take into account vaccination status. Future validation work will focus on QCOVID 2&3 for more recent time periods.

Exposure and Outcomes:

Table 1 and 2 list all exposure and outcomes variables respectively for the QCOVID algorithm, along with a description, variable type (e.g. integer, real, categorical) and possible values.

Table 1: Exposure variables in QCOVID algorithm.

**Variable:**	**Description/Question:**	**Value:**
**Demographic:**		
age	Age in years	Integer: 19-100
sex	Biological sex at birth	Categorical: female, male
town	Townsend Deprivation Score	Real number
ethnicity	Ethnicity	Categorical: White, Indian, Pakistani, Bangladeshi, Other Asian, Caribbean, Black African, Chinese, other ethnic group
homecat	What is your housing category - care home or homeless or neither?	Categorical: neither, care home, homeless
**Clinical:**		
bmi	Body Mass Index (kg/m^2)	Positive real number

For peer review only

chemocat	Have you had chemotherapy in the last 12 months?	Categorical: none, group A, group B, group C
learncat	Do you have a learning disability or Down's Syndrome?	Categorical: learning disability, Down syndrome
renalcat	Chronic Kidney Disease (CKD) stage	Categorical: No serious kidney disease, CKD stage 3, CKD stage 4, CKD stage 5 without dialysis or transplant, CKD stage 5 with dialysis in last 12 months, CKD stage 5 with transplant
diabetescat	Do you have diabetes?	Categorical: none, type 1, type 2
b2_82	Have you been prescribed immunosuppressants four or more times in the previous 6 months?	Categorical: yes, no
b2_leukolaba	Have you been prescribed anti-leukotriene or long acting beta2-agonists (LABA) four or more times in the previous 6 months?	Categorical: yes, no
b2_prednisone	Have you been prescribed oral predisolone containing preparations prescribed four or more times in the previous 6 months?	Categorical: yes, no
b_AF	Do you have atrial fibrillation?	Categorical: yes, no
b_CCF	Do you have heart failure?	Categorical: yes, no
b_asthma	Do you have asthma?	Categorical: yes, no

For peer review only

b_bloodcancer	Have you a cancer of the blood or bone marrow such as leukaemia, myelodysplastic syndromes, lymphoma or myeloma and are at any stage of treatment?	Categorical: yes, no
b_cerebralpalsay	Do you have cerebral palsy?	Categorical: yes, no
b_chd	Do you have coronary heart disease?	Categorical: yes, no
b_cirrhosis	Do you have cirrhosis of the liver?	Categorical: yes, no
b_congenheart	Do you have congenital heart disease or have you had surgery for it in the past?	Categorical: yes, no
b_copd	Do you have chronic obstructive pulmonary disease (COPD)?	Categorical: yes, no
b_dementia	Do you have dementia?	Categorical: yes, no
b_epilepsy	Do you have epilepsy?	Categorical: yes, no
b_fracture4	Have you had a prior fracture of hip, wrist, spine or humerus?	Categorical: yes, no
b_neurorare	Do you have motor neurone disease, multiple sclerosis, myasthenia, or Huntingtons's Chorea?	Categorical: yes, no
b_parkinsons	Do you have Parkinson's disease?	Categorical: yes, no
b_pulmhyper	Do you have pulmonary hypertension or pulmonary fibrosis?	Categorical: yes, no
b_pulmrare	Do you have cystic fibrosis or bronchiectasis or alveolitis?	Categorical: yes, no
b_pvd	Do you have peripheral vascular disease?	Categorical: yes, no
b_ra_sle	Do you have rheumatoid arthritis or SLE?	Categorical: yes, no
b_respcancer	Do you have lung or oral cancer?	Categorical: yes, no

For peer review only

b_semi	Do you have severe mental illness?	Categorical: yes, no
b_sicklecelldisease	Do you have sickle cell disease or severe combined immune deficiency syndromes?	Categorical: yes, no
b_stroke	Have you had a stroke or TIA?	Categorical: yes, no
b_vte	Have you had a thrombosis or pulmonary embolus?	Categorical: yes, no
p_marrow6	Have you had a bone marrow or stem cell transplant in the last 6 months?	Categorical: yes, no
p_radio6	Have you had radiotherapy in the last 6 months?	Categorical: yes, no
p_solidtransplant	Have you had a solid organ transplant (lung, liver, stomach, pancreas, spleen, heart or thymus)?	Categorical: yes, no

Table 2: Outcomes variables in QCOVID algorithm.

**Variable:**	**Description/Question:**	**Value:**
Time to Covid-19 hospitalisation	Time to hospitalisation with RT-PCR confirmed Covid-19 infection in the cohort period in days.	Real number: 0-91
Time to Covid-19 death	Time to death with Covid-19 confirmed or suspected on their death certificate, or confirmed by RT-PCR test, in the cohort period in days.	Real number: 0-91

Whenever available, all variables will be taken as the most recent recorded value in the relevant dataset at the date of entry into the cohort. The Townsend Deprivation Score (TDS) will be

For peer review only

determined by matching available residential location information with output area and the corresponding TDS from the 2011 UK census [9]. Categories for the variable chemocat will be determined using the lookup table in the supplemental materials.

Data cleaning:

The following procedures will be used for data cleaning:

- **diabetes_cat:** If the most recent entry has both type 1 and types 2 recorded, diabetes_cat will be set to type 2.
- **BMI:** The most recently recorded patient BMI within the last 5 years. If the most recently recorded BMI is from more than 5 years ago at the search date, BMI will be set to missing value. Implausible values for BMI (<12 or >70) will be set to missing value.
- **learncat:** If a patient is recorded has having both learning disability and Down's syndrome, learncat will be set to Down's syndrome.

Missing data:

For comorbidities and medication use and treatments, missing values will be taken to mean absence of that factor. Modal substitution will be considered for missing values for ethnicity. For any other missing values of predictor variables, a single imputation will be considered. Outcome variables will not be imputed, and nor will they be included as predictors in the imputation. The following methods may be considered for use in the imputation: predictive mean matching, least squares, logistic and multinomial models, imputation by chained equations.

Statistical Analysis:

Each validation exercise will report a table of cohort characteristics, following Table 2 in [1]. The main performance metrics that will be calculated are R^2 [9], Harrell's C, Royston's D [10] and the Brier score. Different stratifications for these statistics will be considered, including by age, sex and time period. 95% confidence intervals will be reported for R^2, Harrell's C and Royston's D. Graphs of observed and predicted probability of hospital admission and death by vigintile for stratified subgroups will be reported, following [1].

Sample Size:

For peer review only

A preliminary sample size calculation can be done using figures from the original paper [1]. Using the estimated standard deviation of Harrell's C for females in the first time period and assuming Harrell's C is asymptotically normally distributed implies that a sample size of approximately 5,714 would be sufficient to correctly reject a null hypothesis of C=0.5 at significance level 0.05 with probability 80% given a true value of C=0.8. Repeating this calculation for other population subgroups and time periods yields results of a similar magnitude. The samples sizes in the planned studies will be on the order of hundreds of thousands or millions.

Ethics, reporting and dissemination

The ethics approval for the development and validation of QCOVID in England was granted by the East Midlands-Derby Research Ethics Committee [reference 18/EM/0400]. For Scotland, approvals have been obtained by the National Research Ethics Service Committee (REC), South East Scotland 02 (REC number: 12/SS/0201) and the Public Benefit and Privacy Panel for Health and Social Care (reference number: 1920-0279). The data to be used in this study for Wales are available in the SAIL Databank at Swansea University, Swansea, UK. All proposals to use SAIL data are subject to review by an independent Information Governance Review Panel (IGRP). Before any data can be accessed, approval must be given by the IGRP. The IGRP gives careful consideration to each project to ensure proper and appropriate use of SAIL data. When access has been approved, it is gained through a privacy-protecting safe haven and remote access system referred to as the SAIL Gateway. SAIL has established an application process to be followed by anyone who would like to access data via SAIL.[7] Findings will be presented at conferences, published in peer-reviewed journals and to the funders and government COVID-19 advisory bodies as appropriate. Strengthening the Reporting of Observational Studies in Epidemiology (STROBE) and Reporting of studies Conducted using Observational Routinely-collected Data (RECORD) (via the COVID-19 extension) checklists will guide our study findings reporting. The Northern Ireland validation study proposal is under review by the NITRE for HSC data accessed via Northern Ireland Honest Broker Service; an Ethics application has been submitted through IRAS.

Author's Contributions:

AS conceived this protocol. CR, VH, FK, TC, JHC, BH, CC, RAL and JL provided country specific information about available data and analysis plans. SK wrote drafts of this protocol. All authors gave final approval of the version to be published.

Acknowledgments

For peer review only

This work will use data provided by patients and collected by a number of organisations. We would like to acknowledge all patients who shared their information as well as all data providers who make anonymised data available for research. In particular, Public Health Scotland, Public Health Wales, Public Health England, the NHS, the SAIL databank, and the Office for National Statistics.

Funding:

The validation in England will be funded by a grant from the National Institute for Health Research following a commission by the Chief Medical Officer for England. In Scotland, EAVE II is funded by the Medical Research Council [MR/R008345/1] and supported by the Scottish Government. In Wales, ConCOV is supported by the Medical Research Council [MR/V028367/1].

Competing interests:

AS reports grants from NIHR, grants from MRC, and grants from HRR UK, during the conduct of the study. JL and RAL report grants from UKRI Medical Research Council, during the conduct of the study. JHC reports grants from John Fell Oxford University Press Research Fund, grants from Cancer Research UK (CR-UK) grant number C5255/A18085, through the Cancer Research UK Oxford Centre, grants from the Oxford Wellcome Institutional Strategic Support Fund (204826/Z/16/Z), grants from NIHR, during the conduct of the study; personal fees and other from ClinRisk Ltd, outside the submitted work; and JHC is an unpaid director of QResearch, a not-for-profit organisation which is a partnership between the University of Oxford and EMIS Health who supply the QResearch database used for this work. Carol Coupland reports personal fees from ClinRisk ltd, outside the submitted work. JHC, AS, and Carol Coupland were members of the research team involved in the development of the QCOVID risk prediction algorithm. All other authors report no conflict of interest.

Patient and Public Involvement:

There are no plans for Patient and Public Involvement in this research.

Data sharing:

All code used in these analyses will be made publicly available online e.g. through GitHub.

References:

1. Clift AK, Coupland CAC, Keogh RH, Diaz-Ordaz K, Williamson E, Harrison EM, Hayward A, Hemingway H, Horby P, Mehta N, Benger J, Khunti K, Spiegelhalter D, Sheikh A, Valabhji J, Lyons RA, Robson J, Semple MG, Kee F, Johnson P, Jebb S, Williams T, Hippisley-Cox J. Living

For peer review only

risk prediction algorithm (QCOVID) for risk of hospital admission and mortality from coronavirus 19 in adults: national derivation and validation cohort study. BMJ. 2020 Oct 20;371:m3731. doi: 10.1136/bmj.m3731. PMID: 33082154; PMCID: PMC7574532.

2. Townsend, P., Phillimore, P. and Beattie, A. (1988) Health and Deprivation: Inequality and the North. Routledge, London. doi: 10.7748/ns.2.17.34.s66. PMID: 27415096.

3. Qresearch, https://www.qresearch.org/

4. Hippisley-Cox J, Clift AK, Coupland CAC, et al. Protocol for the development and evaluation of a tool for predicting risk of short-term adverse outcomes due to COVID-19 in the general UK population. *MedRxiv* 2020:2020.06.28.20141986-2020.06.28

5. https://www.gov.uk/government/news/new-technology-to-help-identify-those-at-high-risk-from-covid-19

6. EAVE II, The Usher Institute, https://www.ed.ac.uk/usher/eave-ii

7. The Secure Anonymised Information Linkage (SAIL) databank. Available at: https://saildatabank.com

8. Lyons J, Akbari A, Torabi F, Davies G, North L, Griffiths R, Bailey R, Hollinghurst J, Fry R, Turner S, Thompson D, Rafferty J, Mizen A, Orton C, Ellwood-Thompson S, Au-Yeung L, Cross L, Gravenor M, Brophy S, Lucini B, John A, Szakmany T, Davies J, Davies C, Williams C, Emmerson C, Cottrell S, Connor T, Taylor C, Pugh R, Diggle PJ, John G, Scourfield S, Hunt J, Cunningham AM, Helliwell K, Lyons RA. (2020) Understanding and responding to COVID19 in Wales: protocol for a privacy protecting data platform for enhanced epidemiology and evaluation of interventions. BMJ Open 2020;10:e043010. doi:10.1136/ bmjopen-2020-043010

9. 2011 UK Census, Townsend Deprivation Scores, https://www.statistics.digitalresources.jisc.ac.uk/dataset/2011-uk-townsend-deprivation-scores

10. Royston P, Sauerbrei W. A new measure of prognostic separation in survival data. Stat Med. 2004 Mar 15;23(5):723-48. doi: 10.1002/sim.1621. PMID: 14981672.

For peer review only

Sheet3b - Chemotherapy lookup

final classification	map
Group A	1 & 2
Group B	3 & 4
Group C	5

Bench Mark Group	Group
TRASTUZUMAB	1
ZOLEDRONIC ACID	1
DENOSUMAB	1
PEMBROLIZUMAB	1
PERTUZUMAB + TRASTUZUMAB	1
ENZALUTAMIDE	1
ABIRATERONE	1
NIVOLUMAB	1
CETUXIMAB	1
ATEZOLIZUMAB	1
BCG	1
BEVACIZUMAB	1
AFATINIB	1
FULVESTRANT	1
IPILIMUMAB + NIVOLUMAB	1
GOSERELIN	1
METHOTREXATE INTRATHECAL	1
PANITUMUMAB	1
LANREOTIDE	1
DURVALUMAB	1
PERTUZUMAB	1
PAMIDRONATE	1
MITOMYCIN INTRAVESICULAR	1
OCTREOTIDE	1
IMMUNOGLOBULIN	1
ANAGRELIDE	1
LETROZOLE	1
AVELUMAB	1
TRIPLE INTRATHECAL	1
CETUXIMAB + RT	1
BICALUTAMIDE	1
TRIPTORELIN	1
IPILIMUMAB	1
PRISM TRIAL	1
CEMIPLIMAB	1
NERATINIB	1
ANASTROZOLE	1
VISMODEGIB	1
IBANDRONIC ACID	1
TAMOXIFEN	1
CYTARABINE INTRATHECAL	1
EXEMESTANE	1
CYPROTERONE	1
TALIMOGENE LAHERPAREPVEC	1
LEUPRORELIN	1
DEGARELIX	1
ELOQUENT2 TRIAL	1
SILTUXIMAB	1
ELOQUENT TRIAL	1
BICALUTAMIDE + LEUPRORELIN	1
CONFIRM TRIAL	1
FAKTION TRIAL	1
APALUTAMIDE	1
EPIRUBICIN INTRAVESICULAR	1
STILBOESTROL	1
DARBEPOETIN	1
FLUOROURACIL + PANITUMUMAB	1
AVAPRITINIB	1
FURVA TRIAL	1
PERM TRIAL	1
ENZARAD TRIAL	1
BICALUTAMIDE + GOSERELIN	1
NB 13 CIS-RA (ISOTRETINOIN)	1
VORINOSTAT	1
ABIRATERONE + ENZALUTAMIDE	1
PARAGON TRIAL	1
MEDROXYPROGESTERONE	1
KESTREL TRIAL	1
MEGESTROL	1
XILONIX	1
RAMUCIRUMAB	1
CAM-PLEX TRIAL	1
BICALUTAMIDE + TAMOXIFEN	1
LATITUDE TRIAL	1
IPILIMUMAB + PEMBROLIZUMAB	1
QUADRUPLE INTRATHECAL	1
GOSERELIN + LEUPRORELIN	1
MERCAPTOPURINE + VINCRISTINE	1
PROGESTERONE	1
CLODRONIC ACID	1
CYPROTERONE + GOSERELIN	1
GOSERELIN + LETROZOLE	1
CISPLATIN + GEMCITABINE	2
HYDROXYCARBAMIDE	2
CAPECITABINE	2
CISPLATIN + RT	2
PALBOCICLIB	2
FLUOROURACIL	2
CISPLATIN	2
CAPECITABINE + IRINOTECAN	2
CISPLATIN + PEMETREXED	2
DABRAFENIB + TRAMETINIB	2
CAPECITABINE + GEMCITABINE	2
LETROZOLE + PALBOCICLIB	2
PEMETREXED	2
VINORELBINE	2
CISPLATIN + FLUOROURACIL	2
EVEROLIMUS	2
PAZOPANIB	2
SUNITINIB	2
ABEMACICLIB + FULVESTRANT	2
CABOZANTINIB	2
HCX	2
NIRAPARIB	2
CAPECITABINE + RT	2
PEMBROLIZUMAB + PEMETREXED	2
METHOTREXATE	2
GEFITINIB	2
OSIMERTINIB	2
ERLOTINIB	2
RIBOCICLIB	2
FLUOROURACIL + MITOMYCIN + RT	2
OLAPARIB	2
SORAFENIB	2
VINBLASTINE	2
AXITINIB	2
ALECTINIB	2
CAPECITABINE + CISPLATIN + RT	2
CISPLATIN + FLUOROURACIL + RT	2
TIVOZANIB	2
CRIZOTINIB	2
CAPECITABINE + MITOMYCIN	2
CAPECITABINE + VINORELBINE	2
DABRAFENIB	2
ABEMACICLIB	2
LENVATINIB	2
LETROZOLE + RIBOCICLIB	2
CAPECITABINE + MITOMYCIN + RT	2
BEXAROTENE	2
MITOMYCIN	2
INTERFERON	2
PONATINIB	2
VINCRISTINE	2
BUSULPHAN	2
CERITINIB	2
CAPECITABINE + TRASTUZUMAB	2
FLUOROURACIL + MITOMYCIN	2
NINTEDANIB	2
FLUOROURACIL + RT	2
REGORAFENIB	2
ANASTROZOLE + PALBOCICLIB	2
CETUXIMAB + CISPLATIN + FU	2
EVEROLIMUS + LENVATINIB	2
BRIGATINIB	2
LORLATINIB	2
CETUXIMAB + MDG	2
DICE TRIAL	2
BEVACIZUMAB + CAPECITABINE	2
TRASTUZUMAB + VINORELBINE	2
Fulvestrant + Palbociclib	2
Abemaciclib + Letrozole	2
ACELARATE TRIAL	2
VANDETANIB	2
EXEMESTANE + PALBOCICLIB	2
CISPLATIN + TRASTUZUMAB	2
INTERIM TRIAL	2
TRAMETINIB	2
UNIRAD TRIAL	2
MITOXANTRONE	2
CAPECITABINE + LAPATINIB	2
ICON9 TRIAL	2
ATEZOLIZUMAB + BEVACIZUMAB	2
COSMIC TRIAL	2
STAR TRIAL	2
RE-AKT TRIAL	2
BEACON TRIAL	2
DACOMITINIB	2
COLUMBUS TRIAL	2
PACKER MAINTENANCE	2
CAPECITABINE + NERATINIB	2
ANAGRELIDE + HYDROXYCARBAMIDE	2
INTERFERON BETA	2
CISPLATIN + FLUOROURACIL + TRASTUZUMAB	2
QUIZARTINIB	2
ASCIMINIB	2
PAKT TRIAL	2
METHOTREXATE + PEGASPARGASE	2
POLEM TRIAL	2
CISPLATIN + FLUORO + STREPTOZOCIN	2
CAP + RT	2
COMBI-AD TRIAL	2
OLYMPIA TRIAL	2
SPIRIT TRIAL	2
CEDIRANIB	2
FLUOROURACIL + INTERFERON ALFA	2
MILLENNIUM C16021 TRIAL	2
ENCORAFENIB	2
TALAZOPARIB	2
EVEROLIMUS + EXEMESTANE	2
CALYPSO TRIAL	2
INTERFERON ALFA + ZIDOVUDINE	2
IVOSIDENIB	2
SOLO1 TRIAL	2
LU-177	2
MITOMYCIN + RALTITREXED	2
NIMRAD TRIAL	2
SIROLIMUS	2
METHOTREXATE + TRASTUZUMAB	2
PALLET TRIAL	2
TOPARP TRIAL	2
TIPIFARNIB	2
RITUXIMAB + VINBLASTINE	2
IMATINIB + HYDROXYCARBAMIDE	2
METHOTREXATE + VINCRISTINE	2
BELLE3 TRIAL	2
GOSERELIN + PALBOCICLIB	2
PAZ02 TRIAL	2
PIN TRIAL	2
LAPATINIB	2
CASPS TRIAL	2
CISPLATIN + IMATINIB	2
TEMSIROLIMUS	2
LURBINECTIDIN	2
ERDAFITINIB	2
TIMELY TRIAL	2
COBIMETINIB	2
MASITINIB	2
BINIMETINIB	2
CREATE TRIAL	2
IRINOTECAN + MDG	3
OXALIPLATIN + MDG	3
CAPECITABINE + OXALIPLATIN	3
CETUXIMAB + IRINOTECAN + MDG	3
FLUOROURACIL + IRINOTECAN + OXALIPLATIN	3
CVD	3
RITUXIMAB	3
BORTEZOMIB + THALIDOMIDE	3
DARATUMUMAB	3
LENALIDOMIDE	3
IXAZOMIB + LENALIDOMIDE	3
BORTEZOMIB + DARATUMUMAB	3
IMATINIB	3
IBRUTINIB	3
CVP R	3
BORTEZOMIB	3
CISPLATIN + VINORELBINE	3
CABAZITAXEL	3
GEMCITABINE	3
CISPLATIN + ETOPOSIDE	3
MPV	3
TEMOZOLOMIDE	3
TRASTUZUMAB EMTANSINE	3
CETUXIMAB + OXALIPLATIN + MDG	3
CTD	3
OXALIPLATIN + MDG + PANITUMUMAB	3
ECX	3
RUXOLITINIB	3
CARFILZOMIB	3
IRINOTECAN + MDG + PANITUMUMAB	3
POMALIDOMIDE	3
IRINOTECAN + PANITUMUMAB + MDG	3
NILOTINIB	3
CAPECITABINE + CISPLATIN	3
CARDAMON TRIAL	3
BORTEZOMIB + PANOBINOSTAT	3
CYCLOPHOSPHAMIDE + LENALIDOMIDE	3
CVP + OBINUTUZUMAB	3
DASATINIB	3
TEMOZOLOMIDE + RT	3
MUK 9 TRIAL	3
IRINOTECAN	3
CYCLOPHOSPHAMIDE + RITUXIMAB + VINCRISTINE	3
VENETOCLAX	3
CYCLOPHOSPHAMIDE + RITUXIMAB	3
CARBO + FLUOROURACIL	3
CISPLATIN + GEMCITABINE + RITUXIMAB	3
OXALIPLATIN + RALTITREXED	3
CISPLATIN + PEMBROLIZUMAB + PEMETREXED	3
PEG INTERFERON	3
BOSUTINIB	3
OBINUTUZUMAB	3
CAPECITABINE + CETUXIMAB + IRINOTECAN	3
MPT	3
CISPLATIN + ETOPOSIDE + RT	3
IXAZOMIB	3
THALIDOMIDE	3
ETOPOSIDE	3
CAPECITABINE + TEMOZOLOMIDE	3
IRINOTECAN + TEMOZOLOMIDE	3
MERCAPTOPURINE + METHOTREXATE	3
BINIMETINIB + ENCORAFENIB	3
GEMCITABINE + OXALIPLATIN	3
BLINATUMOMAB	3
CMF	3
BEVACIZUMAB + OXALIPLATIN + MdG	3
BEVACIZUMAB + IRINOTECAN + MdG	3
CONCEPT TRIAL	3
OLARATUMAB	3
RALTITREXED	3
CETUXIMAB + CISPLATIN + FLUOROURACIL	3
GEMCITABINE + RT	3
IDELALISIB	3
BEVACIZUMAB + IRINOTECAN	3
BEVACIZUMAB + IRINOTECAN + MDG	3
IRINOTECAN + TEMOZOLOMIDE + VINCRISTINE	3
BOSTON TRIAL	3
CAPE + OXALIPLATIN + TRASTUZUMAB	3
VEMURAFENIB	3
IPM	3
CYCLOPHOSPHAMIDE + ETOPOSIDE	3
PENTOSTATIN	3
IPO	3
CYCLOPHOSPHAMIDE + MTX	3
PANOBINOSTAT	3
OXALIPLATIN	3
IRINOTECAN + BEVACIZUMAB	3
MITOTANE	3
FOCUS4 TRIAL	3
CYCLOPHOSPHAMIDE + VINCRISTINE	3
CAPECITABINE + CETUXIMAB + OXALIPATIN	3
CETUXIMAB + IRINOTECAN	3
COMPLEMENT TRIAL	3
AFLIBERCEPT + FU + IRINOTECAN	3
BEVACIZUMAB + CAPE + OXALIPLATIN	3
CVD + THALIDOMIDE	3
BEVACIZUMAB + PACLITAXEL	3
IRINOTECAN + RALTITREXED	3
IRINOTECAN + VINCRISTINE	3
CARFILZOMIB + CYCLOPHOSPHAMIDE	3
CLARITY TRIAL	3
BEVACIZUMAB + IRINOTECAN + OXALIPLATIN + MDG	3
MMM	3
LENALIDOMIDE + RITUXIMAB	3
CAPECITABINE + OXALIPLATIN + RT	3
PACLITAXEL + BEVACIZUMAB	3
BEVACIZUMAB + CAPE + IRINOTECAN	3
SHAMASH LBCMVD-56	3
PRIMUS 002 TRIAL	3
CYCLO + RITUXIMAB + VINCRISTINE	3
FLUOROURACIL + IRINOTECAN	3
MERCAPTOPURINE	3
PLATO TRIAL	3
CAPITAL TRIAL	3
BALLAD TRIAL	3
PACLITAXEL + RAMUCIRUMAB	3
DARATUMUMAB + LENALIDOMIDE	3
NEPTUNE TRIAL	3
FLUOROURACIL + OXALIPLATIN + TRASTUZUMAB	3
CAPECITABINE + CISPLATIN + TRASTUZUMAB	3
CYCLOPHOSPHAMIDE + FLUOROURACIL + METHOTREXATE	3
METHOTREXATE + RITUXIMAB	3
PREDNISOLONE + VINBLASTINE + RITUXIMAB	3
COMPLEEMENT-1 TRIAL	3
BEVACIZUMAB + OXALIPLATIN	3
CAPECITABINE + IRINOTECAN + PANITUMUMAB	3
IBRUTINIB + VENETOCLAX	3
CCISV	3
SOPHIA TRIAL	3
BORTEZOMIB + VENETOCLAX	3
TREOSULFAN	3
DEPATUXIZUMAB MAFODOTIN + TEMOZOLOMIDE	3
DARATUMUMAB + POMALIDOMIDE	3
RCD TRIAL	3
OFATUMUMAB	3
CAPECITABINE + CETUXIMAB + OXALIPLATIN	3
CAPECITABINE + OXALIPLATIN + PANITUMUMAB	3
CISPLATIN + TEYSUNO	3
GEMCITABINE + OXALIPLATIN + PACLITAXEL	3
BEVACIZUMAB + TEMOZOLOMIDE	3
BORTEZOMIB + LENALIDOMIDE	3
TEYSUNO	3
ELOTUZUMAB + LENALIDOMIDE	3
BORTEZOMIB + PREDNISOLONE + MELPHALAN	3
ALEMTUZUMAB + CYCLOPHOSPHAMIDE + METHOTREXATE	3
MERCAPTOPURINE + METHOTREXATE + TRETINOIN	3
METHOTREXATE + PROCARBAZINE	3
CNS EPEN2007	3
CNS SIOP 2009	3
IRINOTECAN + MITOMYCIN	3
CYCLOPHOSPHAMIDE + GEMCITABINE + VINCRISTINE	3
LENALIDOMIDE + DARATUMUMAB	3
CAPE + CETUXIMAB + OXALIPLATIN	3
Cisplatin + Irinotecan	3
IXAZOMIB + THALIDOMIDE	3
CARFILZOMIB + POMALIDOMIDE	3
CYCLOPHOSHAMIDE + RITUXIMAB	3
ALEMTUZUMAB + ETOPOSIDE	3
SELINEXOR	3
STREPTOZOCIN	3
MCX	3
BENDAMUSTINE + BORTEZOMIB	3
GEMCITABINE + TEGAFUR	3
PATHOS TRIAL	3
CAPECITABINE + OXALIPLATIN + TRASTUZUMAB	3
CETUXIMAB + IRINOTECAN + RALTITREXED	3
IRINOTECAN + OXALIPLATIN + PACLITAXEL	3
PROMISE-MESO TRIAL	3
SCOPE 1 TRIAL	3
CYCLOPHOSPHAMIDE + RITUXIMAB + VINBLASTINE	3
MERCAPTOPURINE + VINBLASTINE	3
BEVACIZUMAB + IRINOTECAN + TEMOZOLOMIDE	3
CETUXIMAB + OXALIPLATIN	3
ACALABRUTINIB	3
BORTEZOMIB + RITUXIMAB	3
CYCLET	3
IBRUTINIB + RITUXIMAB	3
ABC-08 TRIAL	3
CNS HR MBL Cyclo/Vinc	3
FLUOROURACIL + GEMCITABINE	3
MANTA TRIAL	3
CETUXIMAB + OXALIPLATIN + RALTITREXED	3
VIM TRIAL	3
BEVACIZUMAB + OXALIPLATIN + MDG	3
CYCLOPHOSPHAMIDE + THALIDOMIDE + Dexamethasone	3
IRINOTECAN + OXALIPLATIN + PACLITAXEL	3
IRINOTECAN + PANITUMUMAB + RALTITREXED	3
IRINOTECAN + TALAZOPARIB	3
GELOX	3
PACRITINIB	3
PHITT D3 Vincristine / irinotecan	3
MELPHALAN + THALIDOMIDE	3
MM	3
SHINE TRIAL	3
VE	3
AFLIBERCEPT + CAPECITABINE + IRINOTECAN	3
CLL210 TRIAL	3
CISPLATIN + CYCLOPHOSPHAMIDE + ETOPOSIDE	3
CISPLATIN + RALTITREXED + TRASTUZUMAB	3
AFLIBERCEPT + FLUOROURACIL + IRINOTECAN	3
CISPLATIN + CYCLOPHOSPHAMIDE + VINCRISTINE	3
CYCLOPHOSPHAMIDE + TEMOZOLOMIDE	3
IRINOTECAN + TEMOZOLOMIDE + DINUTUXIMAB BETA	3
CARFILZOMIB + CYCLOPHOSPHAMIDE + LENALIDOMIDE	3
PACIFICO TRIAL	3
CYCLOPHOSPHAMIDE + IXAZOMIB	3
LIPOSOMAL DOXORUBICIN + PACLITAXEL	3
Ofatumumab	3
RITUXIMAB + TEMOZOLOMIDE	3
PACLITAXEL	4
CARBOPLATIN + PACLITAXEL	4
CHOP R	4
DOCETAXEL	4
GEMCARBO	4
CARBOPLATIN + ETOPOSIDE	4
EC	4
UKALL2011	4
DOCETAXEL + PERTUZUMAB + TRASTUZUMAB	4
ABVD	4
FEC + DOCETAXEL	4
BENDAMUSTINE + RITUXIMAB	4
CARBOPLATIN	4
CARBOPLATIN + PEMETREXED	4
DOCETAXEL + FLUOROURACIL + OXALIPLATIN	4
ERIBULIN	4
FEC 100	4
CARBOPLATIN + LIPOSOMAL DOX	4
CARBOPLATIN + DOCETAXEL + PERTUZUMAB + TRASTUZUMAB	4
CARBOPLATIN + VINORELBINE	4
EOX	4
CARBOPLATIN + PEMBROLIZUMAB + PEMETREXED	4
FEC	4
FEC 60 OR 75	4
GEMCITABINE + NAB-PACLITAXEL	4
FEC 100 + DOCETAXEL	4
CYCLOPHOSPHAMIDE + DOCETAXEL	4
CHLORAMBUCIL + OBINUTUZUMAB	4
BEVACIZUMAB + CARBOPLATIN + PACLITAXEL	4
CHOP	4
PACLITAXEL + TRASTUZUMAB	4
CAPECITABINE + EPIRUBICIN + OXALIPLATIN	4
PARTNER TRIAL	4
EC + PACLITAXEL	4
CARBOPLATIN + PACLITAXEL + RT	4
PCV	4
CISPLATIN + VINORELBINE + RT	4
BEVACIZUMAB + CARBO + PACLITAXEL	4
FEC + DOCETAXEL + PERTUZUMAB + TRASTUZUMAB	4
R-EPOCH	4
EPIRUBICIN	4
BENDAMUSTINE + OBINUTUZUMAB	4
STAMPEDE TRIAL	4
CYCLOPHOSPHAMIDE + DOCETAXEL + EPIRUBICIN	4
LIPOSOMAL DOXORUBICIN	4
CAV	4
EC + DOCETAXEL	4
CARBOPLATIN + DOCETAXEL	4
CYCLO + DOXORUBICIN + PACLITAXEL	4
ROSCO TRIAL	4
BEACOPP	4
CAPECITABINE + CARBOPLATIN	4
DOCETAXEL + NINTEDANIB	4
CARBOPLATIN + DOCETAXEL + TRASTUZUMAB	4
CISPLATIN + DOCETAXEL + FLUOROURACIL	4
MELPHALAN	4
CHLORAMBUCIL + RITUXIMAB	4
NAB-PACLITAXEL	4
MYELOMA XI TRIAL	4
DOXORUBICIN + IFOSFAMIDE	4
DTPACE	4
DOXORUBICIN + OLARATUMAB	4
ICON8B TRIAL	4
ICE-R	4
FEC + DOCETAXEL + TRASTUZUMAB	4
ESHAP	4
BRENTUXIMAB	4
ESCALATED BEACOP DAC	4
CHOP + OBINUTUZUMAB	4
TIP	4
DOCETAXEL + GEMCITABINE	4
TRABECTEDIN	4
CYCLOPHOSPHAMIDE + DOCETAXEL + DOXORUBICIN	4
EC + DOCETAXEL + PERTUZUMAB	4
RCEOP	4
ECF	4
DOXORUBICIN	4
VDT-PACE	4
TOPOTECAN	4
CHLVPP	4
BENDAMUSTINE	4
AML MYECHILD	4
CYCLOPHOSPHAMIDE + GEMCITABINE + RITUXIMAB + VINCRISTINE	4
CYCLOPHOSPHAMIDE + DOCETAXEL + TRASTUZUMAB	4
GDP	4
OEPA	4
CARBOPLATIN + CYCLOPHOSPHAMIDE + DOXORUBICIN + PACLITAX	4
ETOPOSIDE + IFOSFAMIDE	4
FEC + TRASTUZUMAB	4
PACLITAXEL + PERTUZUMAB + TRASTUZUMAB	4
GEMCITABINE + OXALIPLATIN + RITUXIMAB	4
DOCETAXEL + TRASTUZUMAB	4
EURO-EWING	4
CHLORAMBUCIL	4
ENRICH TRIAL	4
ATEZOLIZUMAB + BEVACIZUMAB + CARBOPLATIN + PACLITAXEL	4
AC	4
CHOEP	4
CARBOPLATIN + RT	4
CYCLOPHOSPHAMIDE + DOCETAXEL + DOXORUBICIN + PERTUZUMA	4
CYCLOPHOSPHAMIDE + TOPOTECAN	4
VIDE	4
LOMUSTINE	4
ICE	4
CYCLOPHOSPHAMIDE + DOXORUBICIN + PERTUZUMAB + TRASTUZU	4
ESHAP R	4
CYCLOPHOSHAMIDE	4
IVE	4
GEMCITABINE + PACLITAXEL	4
RITUXIMAB + VENETOCLAX	4
ECARBOX	4
RMS 2005 TRIAL	4
CARBOPLATIN + VINORELBINE + RT	4
CYCLOPHOSPHAMIDE + ETOPOSIDE + PROCARBAZINE	4
CLADRIBINE	4
BENDAMUSTINE + CYTARABINE + RITUXIMAB	4
PVD	4
IDELALISIB + RITUXIMAB	4
IFOSFAMIDE	4
SCALOP-2 TRIAL	4
FEC + PACLITAXEL	4
CARBOPLATIN + CYCLOPHOSPHAMIDE + EPIRUBICIN + PACLITAXEL	4
CAPECITABINE + CISPLATIN + EPIRUBICIN	4
VIP	4
MIFAMURTIDE	4
ATEZOLIZUMAB + NAB PACLITAXEL	4
DHAP - R	4
IVA	4
ATEZOLIZUMAB + CARBOPLATIN + ETOPOSIDE	4
CISPLATIN + DOXORUBICIN	4
FEC 100 + TRASTUZUMAB	4
MAP	4
VEPEMB	4
IGEV	4
CARBOPLATIN + CYCLOPHOSPHAMIDE + EPIRUBICIN + PACLITAXEL	4
EP	4
BENDAMUSTINE + THALIDOMIDE	4
BORTEZOMIB + PANOBINOSTAT + THALIDOMIDE	4
FEC 100 + DOCETAXEL + TRASTUZUMAB	4
CARBOPLATIN + VINCRISTINE	4
FCARBOST	4
CYTARABINE + METHOTREXATE + RITUXIMAB + THIOTEPA	4
CAP	4
DACARBAZINE + DOXORUBICIN + VINBLASTINE	4
CARBOPLATIN + CETUXIMAB + FLUOROURACIL	4
CYCLO + DOXORUBICIN + VINCRISTINE	4
LIPOSOMAL DAUNORUBICIN + CYTARABINE (Vyxeos)	4
EMA/CO	4
CYCLO + DOCETAXEL + TRASTUZUMAB	4
PMITCEBO-R	4
CARBOPLATIN + EPIRUBICIN + PACLITAXEL	4
GDP R	4
CISPLATIN + PACLITAXEL	4
CYCLOPHOSPHAMIDE + DOCETAXEL + PERTUZUMAB + TRASTUZUMA	4
CVD R	4
EOF	4
NHL 2003	4
CAPECITABINE + STREPTOZOCIN	4
CYCLOPHOSPHAMIDE + ETOPOSIDE + RITUXIMAB + VINCRISTINE	4
ACTICCA-1 TRIAL	4
DACTINOMYCIN + ETOPOSIDE	4
CVP	4
INTERLACE TRIAL	4
MVAC	4
ICON TRIAL	4
IVE-R	4
PLATFORM TRIAL	4
MUK8 TRIAL	4
CARBO + DOCETAXEL + TRASTUZUMAB	4
DACARBAZINE	4
LOMUSTINE + PROCARBAZINE + VINCRISTINE	4
EDP	4
VAI	4
CAPECITABINE + CARBOPLATIN + EPIRUBICIN	4
DOXORUBICIN EMBOLISATION	4
LUTETIUM-177	4
DHAP	4
BEACOP DAC	4
CARBOPLATIN + CETUXIMAB + FU	4
CHLORAMBUCIL + ETOPOSIDE + LOMUSTINE	4
R-CHOEP	4
METHOTREXATE + VINBLASTINE	4
CNS EPENDYMOMA II	4
COMPARE TRIAL	4
CYCLOPHOSPHAMIDE + DOXORUBICIN	4
POMB	4
CARBOPLATIN + NAB-PACLITAXEL	4
CYCLOPHOSPHAMIDE + DOCETAXEL + DOXORUBICIN + TRASTUZUM	4
CISPLATIN + FU + TRASTUZUMAB	4
ESPAC TRIAL	4
EDP + MITOTANE	4
PROCAID TRIAL	4
TAC	4
CYCLOPHOSPHAMIDE + ETOPOSIDE + LOMUSTINE	4
SCOPE2 TRIAL	4
IVAC	4
ABVD R	4
CISPLATIN + DOX + ETOPOSIDE	4
ACCEPT TRIAL	4
VEDEX	4
IVADO	4
CISPLATIN + ETOPOSIDE + IFOSFAMIDE	4
CEV	4
CISPLATIN + IFOSFAMIDE + PACLITAXEL	4
INTERAACT TRIAL	4
CVP R + GEMCITABINE	4
CYCLOPHOSPHAMIDE + CYTARABINE + MERCAPTOPURINE	4
CYCLOPHOSPHAMIDE + EPIRUBICIN + FLUOROURACIL + PERTUZUM	4
GEMCITABINE + TIP	4
CISPLATIN + LOMUSTINE + VINCRISTINE	4
CAPECITABINE + CARBOPLATIN + TRASTUZUMAB	4
BORTEZOMIB + MELPHALAN	4
LOMUSTINE + PROCARBAZINE	4
GEMCITABINE + IFOSFAMIDE + VINORELBINE	4
CARMUSTINE + THIOTEPA	4
R-GEM-P	4
DACTINOMYCIN + VINCRISTINE	4
SPIRE TRIAL	4
DACARBAZINE + GEMCITABINE	4
CYCLOPHOSPHAMIDE + ETOPOSIDE + RITUXIMAB	4
HD EuroNet-PHL-C2 DECOPDAC	4
ALEMTUZUMAB + CYCLOPHOSPHAMIDE	4
DINUTUXIMAB BETA	4
PHITT TRIAL	4
CISPLATIN + ETOPOSIDE + PACLITAXEL	4
CISPLATIN + TOPOTECAN	4
PIXANTRONE	4
CYCLOPHOSPHAMIDE + VINORELBINE	4
DACTINOMYCIN	4
ETOPOSIDE + IFOSFAMIDE + RITUXIMAB	4
IGEV R	4
EURAMOS TRIAL	4
PAD	4
FOXFIRE TRIAL	4
CAPECITABINE + CARBOPLATIN + RT	4
CHLORAMBUCIL + OFATUMUMAB	4
GEMCITABINE + TREOSULPHAN	4
ROMICAR TRIAL	4
NEO-AEGIS TRIAL	4
STREPTOZOCIN + MDG	4
CARBOPLATIN + TOPOTECAN	4
CAPECITABINE + CISPLATIN + STREPTOZOCIN	4
COPP + DACARBAZINE	4
FEC 60 OR 75 + DOCETAXEL	4
CISPLATIN + EPIRUBICIN + FLUOROURACIL	4
CMV	4
CYTARABINE + OXALIPLATIN + RITUXIMAB	4
CYCLO + ETOPOSIDE + VINCRISTINE	4
HD EURONET-PHL	4
FCIST	4
MUK7 TRIAL	4
BENDAMUSTINE + GEMCITABINE + VINORELBINE	4
RATHL TRIAL	4
METHOTREXATE + VINORELBINE	4
R-CHOP / R-DHAP	4
HEPATOBLASTOMA	4
CARBO + LIPOSOMAL DOX + PACLITAXEL	4
CLADRIBINE + RITUXIMAB	4
BLEOMYCIN	4
VDC	4
CHLORAMBUCIL + DEXAMETHASONE + ETOPOSIDE	4
CISPLATIN + EPIRUBICIN	4
EC + PACLITAXEL + PERTUZUMAB	4
EP/EMA	4
CARBOPLATIN + DOCETAXEL + FLUOROURACIL	4
GEMCITABINE + VINORELBINE	4
MUK 12 TRIAL	4
BENDAMUSTINE + BRENTUXIMAB	4
DACARBAZINE + DOXORUBICIN + VINCRISTINE	4
CYCLOPHOSPHAMIDE + DOXORUBICIN + RITUXIMAB	4
CYCLOPHOSPHAMIDE + DOXORUBICIN + ETOPOSIDE + VINCRISTINE	4
ECARBOF	4
CHLORAMBUCIL + LOMUSTINE	4
EPIRUBICIN + FLUOROURACIL + OXALIPLATIN	4
CYCLO + ETOPOSIDE + NELARABINE	4
PEI	4
WILMS REL ICE	4
CISPLATIN + IFOSFAMIDE + MESNA + VINBLASTINE	4
CARBOPLATIN + EPIRUBICIN + FLUOROURACIL	4
CYCLOPHOSPHAMIDE + ETOPOSIDE + PROCARBAZINE + RITUXIMAB	4
BORTEZOMIB + CISPLATIN + CYCLOPHOSPHAMIDE + DOXORUBICIN -	4
PENTOSTATIN + RITUXIMAB	4
CISPLATIN + FLUOROURACIL + STREPTOZOCIN	4
BEVACIZUMAB + CISPLATIN + PACLITAXEL	4
CHLVPP/EVA	4
CARBOPLATIN + DOXORUBICIN	4
PMITCEBO	4
HD EuroNet-PHL-C2 COPDAC	4
CHOP + INTERFERON ALFA + ZIDOVUDINE	4
CISPLATIN + DOCETAXEL	4
GEMCITABINE + RITUXIMAB	4
CVD (NEUROENDOCRINE)	4
CYTARABINE + ETOPOSIDE + TRETINOIN	4
DACARBAZINE + DOXORUBICIN	4
BLEOMYCIN + CISPLATIN + VINCRISTINE	4
FEC + PACLITAXEL + TRASTUZUMAB	4
PROCARBAZINE	4
FLUDARABINE	4
BORTEZOMIB + DOXORUBICIN	4
BLEOMYCIN + CISPLATIN + VINBLASTINE	4
CYCLOPHOSPHAMIDE + DACARBAZINE + VINCRISTINE	4
ETOPOSIDE + RITUXIMAB	4
HD EuroNet-PHL-C2 OEPA	4
CARFILZOMIB + LENALIDOMIDE	4
CISPLATIN + LIPOSOMAL DOXORUBICIN	4
EPSSG	4
CARBOPLATIN + PACLITAXEL + TRASTUZUMAB	4
SELPAC TRIAL	4
CHLORAMBUCIL + VINBLASTINE	4
CARBO + ETOPOSIDE + VINCRISTINE	4
NEOBLADE TRIAL	4
BOP	4
CISPLATIN + DACARBAZINE + VINBLASTINE	4
CYCLOPHOSPHAMIDE + DACTINOMYCIN + VINCRISTINE	4
ETOPOSIDE + LOMUSTINE + VINCRISTINE	4
CYCLO + FLUDARABINE + RITUXIMAB	4
INCA TRIAL	4
METAPHER TRIAL	4
CARBOPLATIN + EPIRUBICIN	4
CARBO + GEMCITABINE + RITUXIMAB	4
CAPE + CARBOPLATIN + CETUXIMAB	4
CARBO + THIOTEPA + TOPOTECAN	4
CARBOPLATIN + FLUOROURACIL + STREPTOZOCIN	4
BEVACIZUMAB + CARBO + GEMCITABINE	4
CARBOPLATIN + ETOPOSIDE + IFOSFAMIDE	4
CISPLATIN + DOXORUBICIN + ETOPOSIDE	4
CARBOPLATIN + CYCLOPHOSPHAMIDE + ETOPOSIDE	4
DACTINOMYCIN + ETOPOSIDE + METHOTREXATE	4
DACTINOMYCIN + CYCLO + VINCRISTINE	4
MUK6 TRIAL	4
POUT TRIAL	4
Bortezomib + DA-EPOCH-R	4
CISPLATIN + CYCLO + DOXORUBICIN	4
DA-EPOCH + BORTEZOMIB	4
RPACE-BOM	4
CYTARABINE + ETOPOSIDE	4
CAPECITABINE + DOCETAXEL	4
EPIRUBICIN + OXALIPLATIN	4
CHOP R + BORTEZOMIB	4
CYCLOPHOSPHAMIDE + CYTARABINE + MERCAPTOPURINE + METHC	4
CARBOPLATIN + NAB-PACLITAXEL + TRASTUZUMAB	4
EC + DOCETAXEL + TRASTUZUMAB	4
IFOSFAMIDE + PACLITAXEL	4
NAB-PACLITAXEL + TRASTUZUMAB	4
AC + DOCETAXEL + TRASTUZUMAB	4
GEMCITABINE + RITUXIMAB + VINORELBINE	4
CARBOPLATIN + EPIRUBICIN + ETOPOSIDE + MELPHALAN	4
DACTINOMYCIN + DOXORUBICIN + VINCRISTINE	4
LOMUSTINE + TEMOZOLOMIDE	4
CARBOPLATIN + ETOPOSIDE + VINCRISTINE	4
CYCLOPHOSPHAMIDE + PACLITAXEL	4
mEOC TRIAL	4
CHOP-R + CYTARABINE	4
CISPLATIN + DOXORUBICIN + METHOTREXATE	4
CISPLATIN + DOX + ETOPOSIDE + MITOTANE	4
OPTIMAL TRIAL	4
CARBOPLATIN + IFOSFAMIDE	4
CYCLOPHOSPHAMIDE + DOXORUBICIN + ETOPOSIDE	4
CYCLOPHOSPHAMIDE + FLUOROURACIL + METHOTREXATE + Trastuz	4
PETREA TRIAL	4
G-CVP	4
CISPLATIN + DACTINOMYCIN + ETOPOSIDE + METHOTREXATE	4
CARBOPLATIN + TRASTUZUMAB	4
CVAD	4
CYCLOPHOSPHAMIDE + DOXORUBICIN + METHOTREXATE + VINCRIS	4
CYCLOPHOSPHAMIDE + FLUDARABINE + THIOTEPA	4
LOPP	4
BUSULPHAN + CYCLO	4
CHLORAMBUCIL + DOXORUBICIN + ETOPOSIDE + PROCARBAZINE +	4
ETOPOSIDE + IFOSFAMIDE + RITUXIMAB + THIOTEPA	4
TIDE	4
MIC	4
PANTHER TRIAL	4
MELPHALAN + TOPOTECAN	4
CARBOPLATIN + ETOPOSIDE + PACLITAXEL	4
CYCLOPHOSPHAMIDE + DOXORUBICIN + PROCARBAZINE + VINCRIST	4
MITOXANTRONE + TRETINOIN	4
RETINOBLASTOMA	4
WT Relapse Carbo/Cyclo/Etop	4
NEMO TRIAL	4
BEVACIZUMAB + LOMUSTINE	4
CISPLATIN + NAB-PACLITAXEL	4
CARBOPLATIN + RALTITREXED	4
QUASAR 2 TRIAL	4
CISPLATIN + GEMCITABINE + PACLITAXEL	4
CYCLOPHOSPHAMIDE + METHOTREXATE	4
Z-DEX	4
PACKER INDUCTION VINCRISTINE + RT	4
BEVACIZUMAB + CARBOPLATIN + NAB-PACLITAXEL	4
DOXORUBICIN + GEMCITABINE	4
OCTOPUS TRIAL	4
ARISTOTLE TRIAL	4
CARBOPLATIN + EPIRUBICIN + METHOTREXATE + VINBLASTINE	4
CARBOPLATIN + FLUOROURACIL + TRASTUZUMAB	4
CISPLATIN + CYCLOPHOSPHAMIDE + DOXORUBICIN	4
DOXORUBICIN + STEPTOZOTOCIN	4
CYCLOPHOSPHAMIDE + ETOPOSIDE + LOMUSTINE + RITUXIMAB	4
RIALTO TRIAL	4
STS Non- Rhabdo EpSSG Vinblastine/MTX	4
ICE TRIAL	4
MVCARBO	4
CABAZITAXEL + CARBOPLATIN	4
CHOP + BORTEZOMIB	4
GDCVP	4
EPIRUBICIN + FLUOROURACIL	4
IDARUBICIN + MERCAPTOPURINE + METHOTREXATE + VINCRISTINE	4
PMITCEBO R	4
CYCLOPHOSPHAMIDE + DOXORUBICIN + PACLITAXEL	4
ARIEL 4 TRIAL	4
CISPLATIN + DACARBAZINE	4
BLEOMYCIN + OXALIPLATIN + VINCRISTINE	4
CNS Germ Cell Tumour II - Non Germinomatous - Standard PEI	4
ETOPOSIDE + IFOSFAMIDE + METHOTREXATE	4
METHOTREXATE + PROCARBAZINE + RITUXIMAB	4
CARBOPLATIN + DACARBAZINE	4
CNS Medbl St Risk <3yrs induction	4
CYCLOPHOSPHAMIDE + IDARUBICIN	4
ReBEL TRIAL	4
ACE	4
BLEOMYCIN + VINCRISTINE	4
CHLVPP/PABLOE	4
CYTARABINE + OXALIPLATIN	4
PHITT D Carboplatin/Doxorubicin	4
BLEOMYCIN + CYCLOPHOSPHAMIDE + ETOPOSIDE + GEMCITABINE +	4
BLEOMYCIN + CYCLOPHOSPHAMIDE + ETOPOSIDE + METHOTREXAT	4
DOXORUBICIN + PEGASPARGASE + VINCRISTINE	4
PAC-E	4
SIOP	4
BENDAMUSTINE + GEMCITABINE + RITUXIMAB + VINORELBINE	4
CYCLOPHOSPHAMIDE + CYTARABINE + POMALIDOMIDE	4
PHITT C CDDP-M	4
ADOC	4
CARBOPLATIN + DOXORUBICIN + MITOMYCIN	4
CARBOPLATIN + ETOPOSIDE + METHOTREXATE	4
CARBOPLATIN + THIOTEPA + TOPOTECAN	4
CYCLOPHOSPHAMIDE + VINBLASTINE	4
HERBY TRIAL	4
HLH Induction W3-8 Etoposide/Dexamethasone/Ciclosporin	4
CARBOPLATIN + ETOPOSIDE + THIOTEPA	4
ERIBULIN + TRASTUZUMAB	4
ALEMTUZUMAB + ETOPOSIDE + METHOTREXATE	4
PHITT D2 Carboplatin / etoposide	4
VIOLA TRIAL	4
BEVACIZUMAB + CARBO + DOCETAXEL	4
CISPLATIN + DOXORUBICIN + IFOSFAMIDE	4
BORTEZOMOB + POMALIDOMIDE	4
EW Rel rEECur Ifosfamide	4
ALEMTUZUMAB + FLUDARABINE + THIOTEPA + TREOSULFAN	4
ALEMTUZUMAB + FLUDARABINE + TREOSULFAN	4
BLEOMYCIN + CISPLATIN + ETOPOSIDE + PACLITAXEL	4
CARBOPLATIN + CYCLO + DOXORUBICIN	4
CNS Ependymoma 99 vinc+cyclo+etop	4
CNS Medbl HR	4
CYCLOPHOSPHAMIDE + MITOXANTRONE + RITUXIMAB + VINCRISTINI	4
EPIRUBICIN + OXALIPLATIN + RALTITREXED	4
FLUOROURACIL + LOMUSTINE	4
PHITT C Carboplatin/Doxorubicin	4
PHITT E2 PLADO	4
WT2001	4
ASPARAGINASE + CYTARABINE + ETOPOSIDE + METHOTREXATE	4
BLEOMYCIN + CYCLOPHOSPHAMIDE + GEMCITABINE + RITUXIMAB +	4
CLADRIBINE + CYCLOPHOSPHAMIDE + RITUXIMAB	4
ESTRAMUSTINE	4
PACE-BOM	4
CARMUSTINE + CYCLOPHOSPHAMIDE + ETOPOSIDE	4
CHLORAMBUCIL + ETOPOSIDE + RITUXIMAB	4
CISPLATIN + CYCLOPHOSPHAMIDE	4
CYTARABINE + IDARUBICIN + METHOTREXATE + RITUXIMAB	4
DACTINOMYCIN + IFOSFAMIDE + VINCRISTINE	4
FMD	4
MAP + MIFAMURTIDE	4
MUK 11 TRIAL	4
CLOFARABINE + CYCLOPHOSPHAMIDE + ETOPOSIDE	4
CLOFARABINE	4
CYCLOPHOSPHAMIDE + DOXORUBICIN + METHOTREXATE + RITUXIM	4
ROVALPITUZUMAB TESIRINE	4
ALEMTUZUMAB + FLUDARABINE	4
CLADRIBINE + CYTARABINE + IDARUBICIN	4
HLH Consol Etop/Dexamthasone/Ciclosporin	4
CARBOPLATIN + PROCARBAZINE	4
CYTARABINE + FLUDARABINE + RITUXIMAB	4
CYTARABINE + IDARUBICIN	4
CYTARABINE + MERCAPTOPURINE + METHOTREXATE + PEGASPARA	4
HELIOS TRIAL	4
IBRITUMOMAB TIUXETAN + RITUXIMAB + Y90	4
Idarubicin + Tretinoin	4
CYCLOPHOSPHAMIDE + DOCETAXEL + EPIRUBICIN + TRASTUZUMAB	4
DOXORUBICIN + METHOTREXATE + MIFAMURTIDE	4
IDARAM	4
BLEOMYCIN + CYCLOPHOSPHAMIDE + ETOPOSIDE + GEMCITABINE +	4
CARBOPLATIN + TEYSUNO	4
CISPLATIN + DOXORUBICIN + METHOTREXATE + MIFAMURTIDE	4
CVP R + ETOPOSIDE	4
STS Non-Rhabdo EpSSG 2005 Ifos	4
STS Non-Rhabdo EpSSG 2005 VA	4
CARBOPLATIN + CYCLOPHOSPHAMIDE + EPIRUBICIN + FLUOROURA	4
CCEP	4
CYCLOPHOSPHAMIDE + NAB-PACLITAXEL	4
DENOSUMAB + ERIBULIN	4
IFOSFAMIDE + VINCRISTINE	4
CISPLATIN + CYTARABINE	4
CYCLOPHOSPHAMIDE + DACTINOMYCIN	4
CYCLOPHOSPHAMIDE + ETOPOSIDE + THALIDOMIDE	4
MEAD (PEG-ASPARAGINASE)	4
R-PCE2BO (ETOPOPHOSï¿½)	4
BEVACIZUMAB + CARBO	4
CARBOPLATIN + CETUXIMAB	4
CARMUSTINE	4
GEMCITABINE + OXALIPLATIN + PEGASPARAGINASE	4
METHOTREXATE + MITOMYCIN + MITOXANTRONE	4
AZACITIDINE	5
BEP	5
UKALL2014	5
CYTARABINE	5
TRIFLURIDINE + TIPIRACIL	5
CYCLOPHOSPHAMIDE	5
DA	5
FLAG + IDARUBICIN	5
FCR	5
AML19 TRIAL	5
FLAIR TRIAL	5
R IVAC	5
BEAM	5
MATRIX TRIAL	5
ARSENIC TRIOXIDE + TRETINOIN	5
ALEMTUZUMAB + FLUDARABINE + MELPHALAN	5
AML18 TRIAL	5
R CODOX-M/ R IVAC	5
R CODOX M	5
CYTARABINE + DAUNORUBICIN + GEMTUZUMAB OZOGAMICIN	5
MYELOMA XII TRIAL	5
RADIUM 223	5
LEAM	5
METHOTREXATE HIGH DOSE	5
ALEMTUZUMAB	5
CHOP R + METHOTREXATE	5
CYTARABINE HIGH DOSE	5
CYCLOPHOSPHAMIDE + FLUDARABINE	5
UKALL60+	5
ATG + BUSULFAN + FLUDARABINE	5
CYCLOPHOSPHAMIDE HIGH DOSE	5
NORDIC	5
AML17 TRIAL	5
UKALLR3	5
FBC	5
ALL INTERFANT 06	5
ALL INTERIM GUIDELINES	5
Yescarta + Fludarabine + Cyclophosphamide	5
CARBOPLATIN + ETOPOSIDE + IFOSFAMIDE + RITUXIMAB	5
STS RHABDO RMS 2005	5
ALEMTUZUMAB + CYCLO + FLUDARABINE	5
EW REL REECUR	5
STS RHABDO VIT0910	5
ARSENIC TRIOXIDE	5
APML	5
UKALL2003	5
ATG + CYCLOPHOSPHAMIDE TBI ALLO	5
ARSENIC TRIOXIDE + ATRA	5
ALL INTENSIFICATION	5
FLAG	5
POMB/ACE	5
CYTARABINE + RITUXIMAB	5
INOTUZUMAB OZOGAMICIN	5
AML16 TRIAL	5
AML 18 PILOT TRIAL	5
SMILE	5
BLEOMYCIN + CARBOPLATIN + ETOPOSIDE	5
MIDOSTAURIN	5
ALL PH+VE	5
ALEMTUZUMAB + BUSULFAN + CYCLOPHOSPHAMIDE	5
BLEOMYCIN + CARBOPLATIN + CISPLATIN + VINCRISTINE	5
LACE	5
CODOX M	5
CYTARABINE HD + METHOTREXATE HD	5
FAB LMB	5
ALL Interim Guide 2019 Induction A	5
LI-1 TRIAL	5
IDARUBICIN	5
AMSACRINE + ATG+ BUSULFAN + CYTARABINE + FLUDARABINE	5
AMSACRINE + ATG + BUSULFAN + CYTARABINE + FLUDARABINE	5
Kymriah + Fludarabine + Cyclophosphamide	5
DOXORUBICIN + HD MTX	5
BEAM + ALEMTUZUMAB	5
DA-EPOCH	5
DAUNORUBICIN + CYTARABINE + MIDOSTAURIN	5
ALL Interim Guide 2019 Augmented BFM Consolidation C	5
FLUDARABINE + MELPHALAN	5
ALEMTUZUMAB + PENTOSTATIN	5
IFOSFAMIDE + LIPOSOMAL DOXORUBICIN	5
ATG	5
CHOP-R + CYTARABINE HIGH DOSE	5
ALL MAINTENANCE	5
CYCLOPHOSPHAMIDE + CYTARABINE + DOXORUBICIN + RITUXIMAB -	5
CISPLATIN + DACTINOMYCIN + ETOPOSIDE + FILGRASTIM + METHOT	5
ALEMTUZUMAB + CYCLOPHOSPHAMIDE + FLUDARABINE	5
AML DOWNS	5
ALL Interim Guide 2019 Delayed Intensification A	5
ACE (GERM CELL)	5
IDARUBICIN + TRETINOIN	5
ALL Interim Guide 2019 Maintenance	5
ALL Interim Guide 2019 Standard BFM Consolidation B	5
UKALL 60+ TRIAL	5
ALL Interim Guide 2019 Standard Interim Maintenance A	5
ALL Interim Guide 2019 Induction B	5
DAUNORUBICIN + PEGASPARAGINASE + VINCRISTINE	5
AML INTERIM GUIDELINES	5
CYTARABINE + METHOTREXATE + RITUXIMAB	5
ALL INDUCTION	5
FIGARO TRIAL	5
CYTARABINE + ETOPOSIDE + IFOSFAMIDE + METHOTREXATE + RITU	5
TRANSPLANT BEAM	5
GEMTUZUMAB OZOGAMICIN	5
DAUNORUBICIN + VNCRISTINE	5
TRANSPLANT CYTARABINE + ETOPOSIDE + LOMUSTINE + MELPHALA	5
MACE	5
TRANSPLANT ALEMTUZUMAB + FLUDARABINE + MELPHALAN	5
NELARABINE	5
UKALL XII	5
ALL R T-CELL CONSOLIDATION	5
METHOTREXATE + RITUXIMAB + TEMOZOLOMIDE	5
ADE	5
TRANSPLANT CYCLOPHOSPHAMIDE + FLUDARABINE (+/- TBI)	5
CARBOPLATIN + CYCLOPHOSPHAMIDE + ETOPOSIDE + PACLITAXEL	5
ALL CONSOLIDATION	5
ETOPOSIDE + IFOSFAMIDE + MTX	5
ROMAZA TRIAL	5
ALL Int Guide B BFMCons	5
CLOFARABINE + CYTARABINE	5
CYTARABINE IT + METHOTREXATE	5
FLA	5
ALL Int Guide A Ind	5
CYTARABINE + METHOTREXATE	5
ALL Interim Guide 2019	5
IDARUBICIN + MITOXANTRONE + TRETINOIN	5
CYTARABINE + FLUDARABINE	5
AZACITIDINE + VENETOCLAX	5
ALL Int GdeC D Int PhIV reind PAsp	5
BENDAMUSTINE + CYTARABINE + ETOPOSIDE + MELPHALAN	5
CYTARABINE + ETOPOSIDE + MITOXANTRONE	5
BUSULFAN + FLUDARABINE	5
CODOX M + IVAC	5
ALEMTUZUMAB + LEAM	5
BUSULFAN + CYCLOPHOSPHAMIDE	5
CYTARABINE + MIDOSTAURIN	5
ERWINIA ASPARAGINASE	5
TRANSPLANT ALEMTUZUMAB + CYTARABINE + ETOPOSIDE + LOMUS	5
111 TRIAL	5
AMSACRINE + BUSULFAN + CYTARABINE + FLUDARABINE	5
ALL Interim Guide 2019 Consolidation A	5
FLUDARABINE + THIOTEPA + TREOSULFAN	5
AML Relapsed FLA - Idarubicin	5
TRIFLURIDINE TIPIRACIL	5
AZACITIDINE + RUXOLITINIB	5
BLEOMYCIN + CISPLATIN + ETOPOSIDE + METHOTREXATE + VINCRIS	5
ATG + BUSULFAN + FLUDARABINE + THIOTEPA	5
IMATINIB + MERCAPTOPURINE + VINCRISTINE	5
ATG + CYCLOPHOSPHAMIDE + FLUDARABINE	5
BUSULFAN + MELPHALAN	5
CANTATA TRIAL	5
BLEOMYCIN + CISPLATIN + ETOPOSIDE	5
ATG + CYCLOPHOSPHAMIDE	5
BLEOMYCIN + CYCLOPHOSPHAMIDE + ETOPOSIDE + GEMCITABINE +	5
CNS ATRT Doxorubicin/Ifosfamide/Carboplatin/Etoposide/Methotrexate	5
CYCLOPHOSPHAMIDE + CYTARABINE + DAUNORUBICIN + MERCAPT(	5
DOXORUBICIN + METHOTREXATE	5
ATG + FLUDARABINE	5
CAPECITABINE + LOMUSTINE	5
METHOTREXATE HIGH DOSE + RITUXIMAB	5
ALL Interim Guide 2019 Standard Interim Maintenance B	5
METHOTREXATE HD + VINCRISTINE	5
GILTERITINIB	5
BUSULFAN + CYCLOPHOSPHAMIDE + FLUDARABINE + THIOTEPA	5
CYTARABINE + Gemtuzumab Ozogamicin	5
MA	5
BEAM + FLUDARABINE	5
AMSACRINE + CYTARABINE	5
CYTARABINE + FLUDARABINE + IDARUBICIN	5
DAUNORUBICIN	5
TRANSPLANT ATG + FLUDARABINE + THIOTEPA + TREOSULFAN	5
GCLAC	5
BUSULFAN + FLUDARABINE + METHOTREXATE	5
IMATINIB + MERCAPTOPURINE + METHOTREXATE	5
ALL Int Guide B Int Maint	5
TRANSPLANT ALEMTUZUMAB + CYCLOPHOSPHAMIDE + FLUDARABIN	5
DOXORUBICIN + IFOSFAMIDE + VINCRISTINE	5
BUSULFAN + THIOTEPA	5
CLOFARABINE + CYTSARABINE + IDARUBICIN	5
ALL NOPHO	5
AMSACRINE + ATG + CYCLOPHOSPHAMIDE + CYTARABINE + FLUDAR	5
CISPLATIN + ETOPOSIDE + METHOTREXATE	5
ALL Interfant 06 ADE	5
ALEMTUZUMAB + FLUDARABINE + LEAM	5
ETOPOSIDE + MELPHALAN	5
IDARUBICIN + IMATINIB + VINCRISTINE	5
ALL Interim Guide 2019 Induction C	5
TRANSPLANT BUSULFAN + FLUDARABINE	5
ALL Interim Guide 2019 NOPHO C	5
CYCLOPHOSPHAMIDE + CYTARABINE + ETOPOSIDE + METHOTREXA	5
CLADRIBINE + CYTARABINE + DAUNORUBICIN	5
TRANSPLANT AMSACRINE + ANTITHYMOCYTE IMMUNOGLOBULIN (R/	5
TRANSPLANT ATG + BUSULFAN + FLUDARABINE + THIOTEPA	5
CLADRIBINE + CYTARABINE	5
DE ANGELIS	5
FORODESINE	5
ATG + FLUDARABINE + MELPHALAN	5
ATG + FLUDARABINE + THIOTEPA + TREOSULFAN	5
TRANSPLANT FLUDARABINE + THIOTEPA + TREOSULFAN	5
CARBOPLATIN + CYTARABINE + ETOPOSIDE	5
CYCLOPHOSPHAMIDE + ETOPOSIDE + NELARABINE	5
DECITABINE	5
MIDAC	5
BLEOMYCIN + CYCLOPHOSPHAMIDE + ETOPOSIDE + GEMCITABINE +	5
BLEOMYCIN + CYCLOPHOSPHAMIDE + ETOPOSIDE + GEMCITABINE +	5
CYTARABINE + VINCRISTINE	5
IDARUBICIN + METHOTREXATE + VINCRISTINE	5
MTX HD + PROCARBAZINE + VINCRISTINE	5
BEAM R	5
BUSULFAN + CYCLOPHOSPHAMIDE + FLUDARABINE	5
CARBOPLATIN + CYTARABINE + RITUXIMAB	5
CYTARABINE + ETOPOSIDE + IMATINIB	5
CYTARABINE + ETOPOSIDE + METHOTREXATE	5
ALL/LBL T Cell Relapse Intensification	5
BLEOMYCIN + CYCLOPHOSPHAMIDE + ETOPOSIDE + MITOXANTRONI	5
CARBOPLATIN + ETOPOSIDE + IFOSFAMIDE + PACLITAXEL	5
CISPLATIN + CYCLOPHOSPHAMIDE + CYTARABINE + DOXORUBICIN +	5
DAUNORUBICIN + METHOTREXATE + PEGASPARAGINASE + VINCRIS	5
CNS HGG CISPLATIN/ETOPOSIDE	5
CYTARABINE + METHOTREXATE HD	5
ALL UKALL2011 TRIAL + ALL UKALL2011 Intensfication MTX	5
TRANSPLANT ATG + ETOPOSIDE (+/- TBI)	5
ALL/LBL T Cell Relapse Consolidation	5
YTTRIUM-90	5
ALL INTENSIFICATION MTX	5
AML RELAPSED	5
CAMELLIA TRIAL	5
SPANISH MAINTENANCE	5
ALL/LBL T Cell Relapse NECTAR Cyclophosphamide, etoposide, nelarabine and IT	5
HORMONES	1
CYTARABINE + METHOTREXATE + PEGASPARAGINASE	5
CHOP R + LIPOSOMAL DOXORUBICIN	3
CYCLOPHOSPHAMIDE + DACTINOMYCIN + DOXORUBICIN + VINCRISTINE	5
TRIAL	6
CYCLOPHOSPHAMIDE + FLUDARABINE + MITOXANTRONE	3
CYCLOPHOSPHAMIDE + LIPOSOMAL DOX	3
VELIPARIB	1
CARBOPLATIN + IRINOTECAN	3
